# Multicomponent Domino Reaction in the Asymmetric Synthesis of Cyclopentan[c]pyran Core of Iridoid Natural Products

**DOI:** 10.3390/molecules25061308

**Published:** 2020-03-13

**Authors:** Alejandro Manchado, Victoria Elena Ramos, David Díez, Narciso M. Garrido

**Affiliations:** Dpto. de Química Orgánica, Facultad de Ciencias Químicas, Universidad de Salamanca, Plaza de los Caídos 1-5, 37008 Salamanca, Spain; alex92mc@usal.es (A.M.); velenaramos@yahoo.com (V.E.R.); ddm@usal.es (D.D.)

**Keywords:** chiral lithium amide, asymmetric aza-Michael addition, asymmetric domino reaction, multicomponent reaction, cyclopentan[c]pyran, iridoid, nepetalactone

## Abstract

The asymmetric synthesis of a compound with the cyclopentan[c]pyran core of iridoid natural products in four steps and 40% overall yield is reported. Our methodology includes a one-pot tandem domino reaction which provides a trisubstituted cyclopentane with five new completely determined stereocenters, which were determined through 2D homo and heteronuclear NMR and n.O.e. experiments on different compounds specially designed for this purpose, such as a dioxane obtained from a diol. Due to their pharmaceutical properties, including sedative, analgesic, anti-inflammatory, CNS depressor or anti-conceptive effects, this methodology to produce the abovementioned iridoid derivatives, is an interesting strategy in terms of new drug discovery as well as pharmaceutical development.

## 1. Introduction

Iridoids are a very extensive family of secondary metabolites. They are found in both terrestrial and marine flora and fauna [1,2,3]. Although usually found in their glucoside form, free iridoids as well as secoiridoids are also abundant in Nature. As free iridoids, they are precursors of biologically active alkaloids and several studies have demonstrated their interesting pharmaceutical properties, as hematoprotective or *N*-oxide inhibitor agents [4,5,6]. As glycosides, they are commonly found in plants, such as in the genus *Nepeta*, and, since they are structurally cyclopentane pyran monoterpenoids, they represent a link between terpenes and alkaloids [7]. Recent studies also suggest they are cell proliferation inhibitors, opening an interesting window into cancer treatment [8,9] as well as viral protein P (Vpr) inhibition [10]. Finally, as secoiridoids, they possess lots of biological activities such as antioxidant, anti-inflammatory, or anti-atherogenic properties, among others [11,12,13]. This family of compounds has also been extensively used in folk medicine plant treatments, all around the globe, as a remedy against coughs, wounds or skin disorders, as well as bitter tonics, antipyretics or sedatives [14,15]. 

The genus *Nepeta*, with its bigger diversity located in the Mediterranean area, have demonstrated, in recent studies, that some extracts from different plants possess very interesting therapeutic properties, such as anti-inflammatory or analgesic effects, due to the abundant presence of nepetalactone derivatives [16,17]. This opens a promising researching area, since morphine use, nowadays, is responsible a lot of dependency and deaths. Also, recent studies have demonstrated the high diversity of pharmacological properties of nepetalactone compounds [8,18,19]. They show both sedative and analgesic properties, as well as anti-inflammatory and CNS depressor effects, as new studies suggest this kind of compound is able to pass through the blood brain barrier [16]. Plus, recent studies have demonstrated nepetalactone derivatives present anticonceptive activity as well as insect repellent ability [19,20,21,22]. 

**Figure 1:**
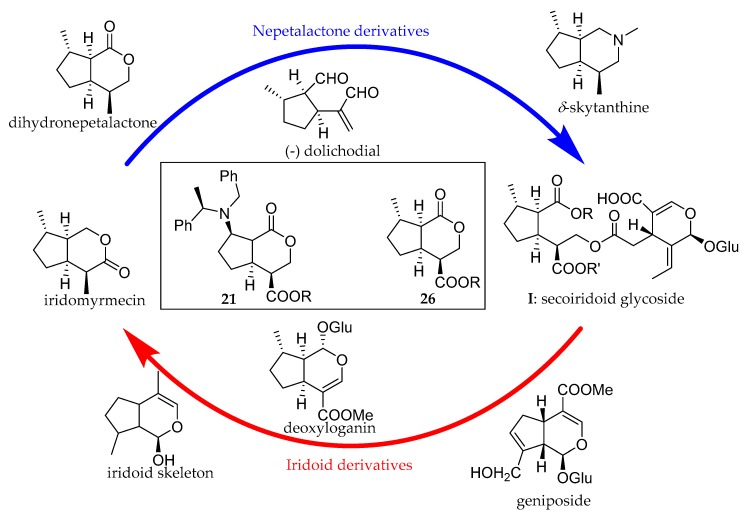
Some main nepetalactone and iridoid skeletons.

We have developed an asymmetric synthesis-based route, which enabled us to obtain the 4*β*,4a*α*,7*α*,7a*α*-dihydronepetalactone analogue **26** (Figure 1,) in very good yield while controlling all chiral centers. This compound is an advanced intermediate towards nepetalactone and iridoid skeletons which have been demonstrated to possess very interesting analgesic properties. Some examples of nepetalactones and iridoids accessible using our methodology are illustrated in Figure 1, where the relevance of the recently isolated secoiridoid **I** from *Fraxinus americana* L.[23] with an identical cyclopentane core can be mentioned, highlighting the importance of **26** for its synthesis [23,24,25]. 

Davies et al. have published a comprehensive review concerning the development, scope and applications of the conjugate additions of enantiomerically pure lithium amides (which act as chiral ammonia equivalents) in 2005, and an update covering 2005–2011 was published in 2012. A further update was recently published in 2017 [26,27,28], dealing with all the characteristics of the asymmetric addition, and recently, we have published a chapter describing methods for the synthesis of lithium amides and their applications in C-N and C-C bond formation reactions, including stereoselective transformations [29].

**Scheme 1:**
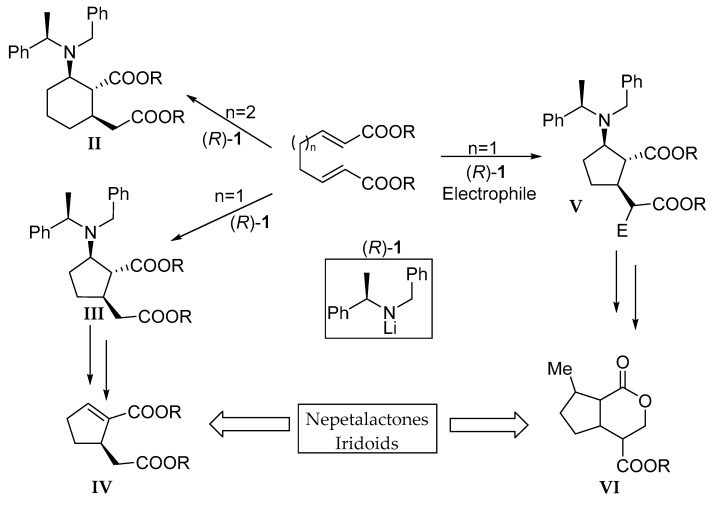
Previous and current studies of domino reactions.

We have demonstrated the use of chiral lithium (*α*-methylbenzyl)benzylamide (*R*)- or (*S*)-**1**) in different domino reactions. We first published that a chiral lithium amide could initiate an asymmetric conjugate addition cyclization of nona-2,7-diendioate to generate chiral cyclohexane derivatives (Scheme 1, **II**) [30,31,32,33], and applied it to the synthesis of (1*R*,5*R*,9*R*)-2-azabicyclo-[3.3.1]nonane-9-carboxylic acid (morphanic acid), with a morphan scaffold [34], which was used in the synthesis of a new class of opioid receptor ligands [32]. In the same way, when octa-2,6-diendioate was used we stereoselectively obtained the 2-amino-5-carboxy-methyl-cyclopentane-1-carboxylate skeleton (Scheme 1, **III**) [35,36,37], and applied it to the synthesis of (*R*) and (*S*)-methyl(-methoxycarbonylcyclopent-2-enyl)acetate **IV** and (*R*)- and (*S*)-2-(2-hydroxymethyl-cyclopent-2-enyl)ethanol, useful homochiral synthons for monoterpenes [35] and to the asymmetric synthesis of all the stereoisomers of 2-amino-5-carboxymethyl-cyclopentane-1-carboxylic acid [36]. We have later shown a novel domino reaction: allylic acetate rearrangement stereoselective Ireland-Claisen rearrangement and asymmetric Michael addition [38,39,40,41]. A protocol starting from Baylis–Hillman adducts using chiral lithium amide (*R*)-**1** to afford *δ*-aminoacids, which can be transformed into piperidines [40] Furthermore we get ready access to phenethylamines from (*N*-*α*-methylbenzyl)-*N*-benzyl *β*-aminoacids obtained by Michael addition of (*R*)-**1** to *α-β*-unsaturated ester, by domino reaction initiated in a Barton decarboxylation followed by a 1,4-phenyl radical rearrangement (1,4-PhRR) [42].

**Scheme 2:**
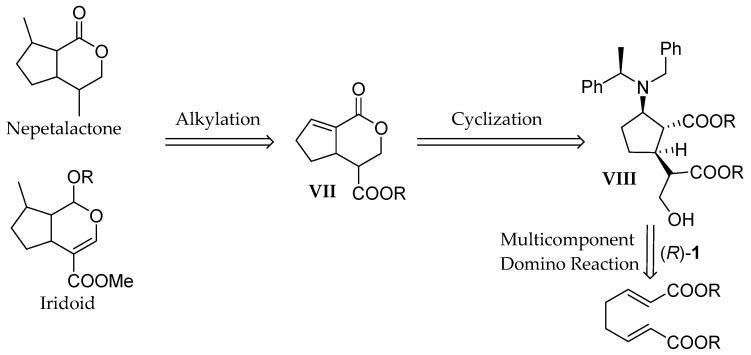
Retrosyntheses of nepetalactones and iridoids from octadiendioate.

Herein, as shown in retrosynthetic Scheme 2, following the aforementioned domino reaction access to cyclopentane derivatives, now in a three component version, we report the synthesis of highly functionalized cyclopentanes VIII with total stereocontrol of the four new stereocenters generated in one-pot and their application via cyclization (VII) to the synthesis of important derivatives from the point of view of their pharmaceutical activities, such as nepetalactones and iridoids.

## 2. Results and Discussion

### 2.1. Domino and Tandem Reaction with Benzaldehyde

Cyclopentane derivative **III** has been synthesized, as already mentioned, by adding (*R*)-**1** to **2** without a subsequent electrophile addition [35,36,37]. We wanted to introduce an additional carbon atom in the α-position of the alkoxycarbonylmethyl group, so an electrophile was necessary after performing the abovementioned domino reaction in a tandem multicomponent reaction protocol. Thus, we decided initially to use benzaldehyde as electrophile so the reaction scope, as well as the stereochemistry of the two new generated stereocenters, could be studied.

**Scheme 3:**
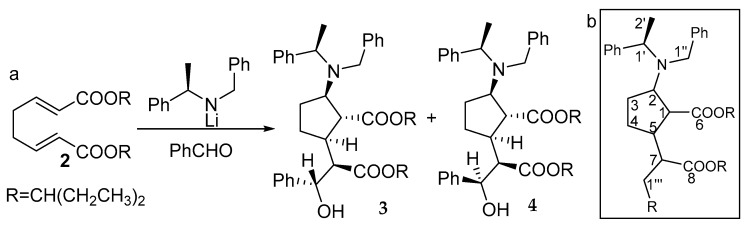
(**a**) Multicomponent domino reaction towards **3** and **4.** (**b**) Numbering used for these compounds.

When the addition of (*R*)-**1** (1.6 eq) to di-3-pentyl octa-2,6-diendioate is performed at −78 °C and then after one hour, benzaldehyde is added and the reaction allowed to reach room temperature, a mixture from which alcohol **3** (23%) and the C-1‴ epimer **4** (45%) (Scheme 3b, showing the numbering of these derivatives) are isolated by column chromatography is obtained. The 3-pentyl ester was chosen as it largely prevents the 1,2 addition reactions of the lithium amide to the ester (methyl) group, leading to the corresponding amide and, at the same time, it is easy to hydrolyze under basic conditions. In addition, with the pentyl ester in the domino reaction to obtain the cyclic compound **III**, we have observed an increase in d.r.: 92:8 vs. 91:9 when using the methyl ester [36].

The results of the ^1^H-^13^C heteronuclear correlation experiments at one and several bonds (normal and long range HMQC and HMBC), shown in Table 1 and in Supplementary Materials, allow to corroborate their structure and the full assignment of the ^1^H- and ^13^C-NMR data.

**Figure 2:**
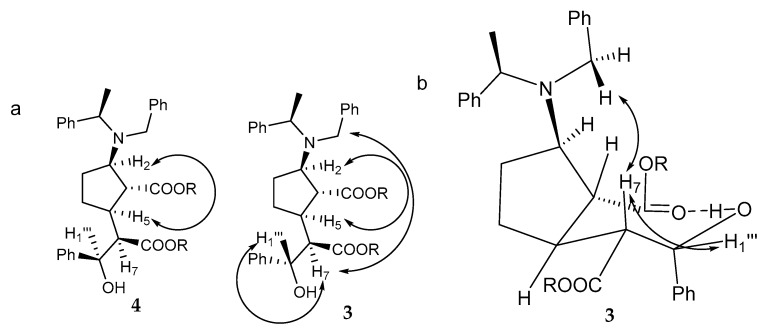
(**a**) ^1^H NMR stereochemical analysis of major and minor diastereoisomers **4** and **3**. (**b**) Proposed intramolecular H-bond for **3**.

The observed n.O.e (Figure 2a) between H2 and H5 for these compounds and the coupling constant in **4** for H1 at 3.23 ppm (dd, *J* = 10.1 and 9.3 Hz) confirm the predicted *trans*, *trans-* trisubstituted cyclopentane ring. Compounds **3** and **4** show very similar ^1^H and ^13^C data according to the C1′’’ different configuration, but full stereochemical characterization was possible by chemical transformations and spectroscopic analysis, as it will be detailed later.

Once the stereochemistry of all sterocenters of **3** and **4** was known (*vide infra*), it was possible to explain the experimental observations, such as the *J* of the hydroxyl hydrogen at 3.92 ppm (d, *J* = 9.1) and H1′’’ at 4.87 ppm (dd, *J* = 9.1 and 3.7 Hz) in **3** and 3.04 ppm (d, *J* = 3.5 Hz); 4.64 ppm (dd, *J* = 10.0 and 3.5 Hz) respectively for **4**.

The n.O.e with H1 by saturation of H7 and a hydrogen of the benzyl C2′ in **3** allowed us to conclude the existence of a hydrogen bridge in a cyclooctane system formed between the hydroxyl group and the C6 carbonyl as shown in Figure 2b. These observations could be used for the stereochemical determination of the centers generated in the aldol condensations of these systems.

**Scheme 4:**
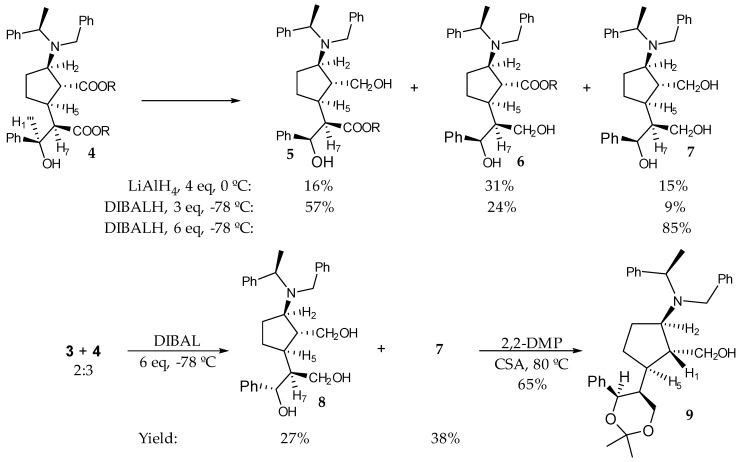
Reduction reactions of **3** and **4** epimers and dioxane **9** formation.

Then, different reductions were performed as shown in Scheme 4. When **4** was treated both with LiAlH_4_ (2 eq.) at 0 °C and with DIBALH (3 eq.) at −78 °C; **5**, **6** and the triol **7** were obtained. HMQC and HMBC studies (Table 1) show correlation between H7 and C-8 ester in diol **5**, and correlations between C-6 and H1 and H2 in **6**. Thus, suggesting that the formation of the proposed hydrogen bond (Figure 2b) is favorable at low temperatures, because of both entropic contributions and the Boltzmann distribution, and, therefore, favors the reduction of the C6 ester and, additionally, because of the C-8 ester within this structure is blocked by the phenyl and cyclopentyl groups, bringing on the formation of **5**. 

When the reduction of the mixture **3**+**4** (2:3 ratio) was performed with excess DIBALH, the triols **7** and **8** were obtained accordingly, and, under these conditions, reduction of **4** afforded **7** in 85% isolated yield that was converted in dioxane derivative **9** (61%) under standard condition when it was treated with dimethoxypropane (Scheme 4).

**Figure 3:**
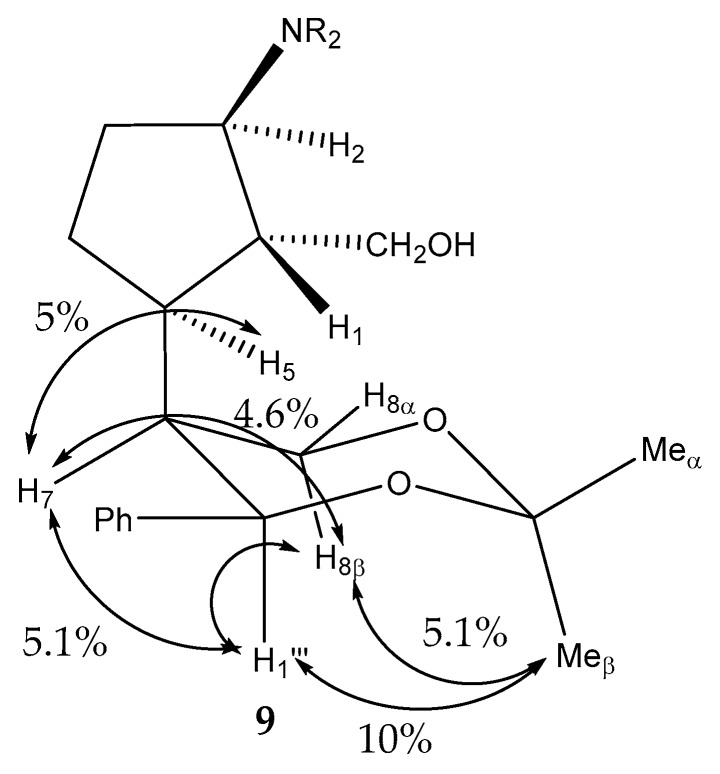
Significant n.O.e correlations observed for **9**.

Homonuclear COSY and n.O.e. (Figure 3) experiments allowed to determine the stereochemistry in the newly generated centers within the dioxane ring (see Supplementary Materials). Coupling constants *J* = 12.1 Hz for H8*β* and H8*α* and 0 and 2.9 Hz, respectively, for H7, therefore indicating an equatorial disposition for these protons (H7 and H8*α*). The most relevant n.O.es: H1-H7, H7-H1′’’, H1′’’-H8*β*, H1′’’-Me*β*, H8*β*-Me*β* and H7-H8*β*, indicate a *cis* arrangement for H7, H1′’’, H8*β* and Me*β* (1.49 ppm), thus, fixing all stereocenters for **9** as: (1*R*,2*R*,5*S*, 7*R*,1′*R*,1′’’*R*) and the same remains for compounds **7** and **4**.

**Scheme 5:**
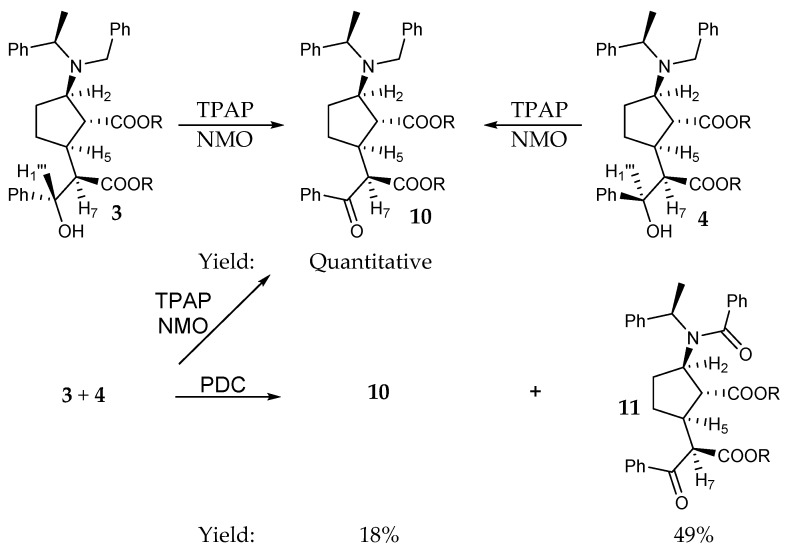
Oxidation reactions of **3** and **4.**

As **3** (minor adduct) was presumed to be C1′’’ epimer of **4**, both the mixture and each compound separately was oxidized with TPAP, always providing the ketone **10** quantitatively, as shown in Scheme 5, so the configuration in **3** was established as (1*R*,2*R*,5*S*,7*R*,1′*R*,1′’’*S*). When a mixture of **3** and **4** was oxidized with PDC the diketone **11** was obtained together with **10**, due to further oxidation of the benzylic carbon.

Thus, in summary, we have corroborated by n.O.e experiments the stereochemistry of the three stereocenters of the cyclopentane (achieved by, and related to, the auxiliar chiral (*R*)-**1**), that we have already described and settled by x-ray crystallography [36]). Likewise, the stereochemistry of the two new stereocenters generated in the subsequent aldol reaction were established by n.O.e experiments on a dioxane derivative of the major diastereoisomer. The other diastereoisomer was the C1′’’ epimer of the previous one, since the same compound was obtained by oxidation of this center in both epimers.

### 2.2. Proposed Mechanism

We have reported an exhaustive mechanistic revision [43] of the originally proposed mechanism [44], developing a quantum mechanics/molecular mechanics protocol for the asymmetric aza-Michael reaction of homochiral lithium benzylamides to *α-β*-unsaturated esters resulting in a *Z*-enolate prior to electrophilic quenching [45].

**Scheme 6:**
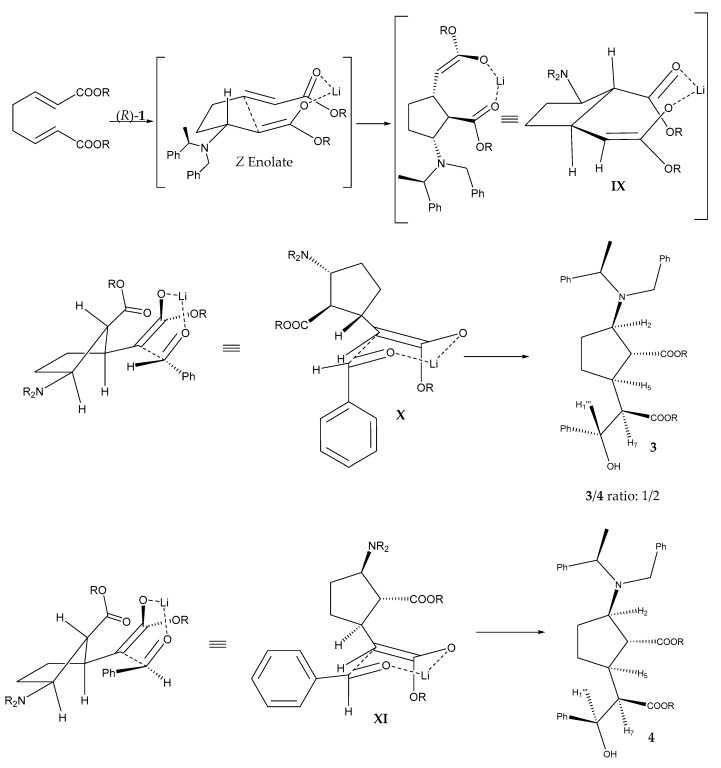
Mechanistic explanation of the different TS toward **3** and **4** (1:2 ratio).

The second Michael addition in the domino reaction gives rise to the living Z-enolate (**IX**, Scheme 6) with the Si face accessible for the electrophile. Two approaches are possible for the incoming benzaldehyde: like, throughout its Si face (**X**) or unlike, Re face of the aldehyde (**XI**), a 1:2 ratio for **3**/**4** is observed, probably, due to the contrast of unfavourable axial position of the phenyl group in **X**, over steric impediment of the cycle substituents in **XI**. This ratio changes with the size of the aldehyde (vide infra)

### 2.3. Reaction Scope

The next step was to react new electrophiles such as different aldehydes, ketones, epoxide and chloroformate to explore the reaction scope. Formaldehyde is an interesting substrate in regards to this methodology due to the possibility of its applications to the synthesis of iridoid natural products.

Results are shown in the following table.

When using cinnamaldehyde as electrophile (Table 2, entry 2), **12** (30%) and **13** (27%) were obtained. The NMR signals, taking into account the additional double bond, are similar to those of **3** and **4**, and, based on the spectroscopic considerations established above, especially the hydrogen bridge bond, allow us to establish the stereochemistry of these compounds, the hydroxyl proton in **12** at 3.33 ppm (d, *J* = 10 Hz) and in **13** at 2.85 ppm (d, *J* = 2.5 Hz) accordingly. In this case the epimer ratio is close to 1:1, in accordance to the proposed mechanism (Scheme 6) due to increased interaction in TS **XI**.

When formaldehyde was used (entry 3), **14** (80%) was the only compound isolated after column chromatography. Now the C6 configuration is *S,* contrary to previous one, as determined in subsequent derivatives (*vide infra*). Due to the small size of the formaldehyde molecule, it approaches the *Re* face probably in a tricoordinate Li TS within **IX**, producing **14**.

To explore the reaction scope acetone and diphenylketone (entry 4 and 5) were used, giving rise to the multicomponent adducts **15** (27%) and **17** (52%) respectively, together with the reported [36] domino adduct **16** in 25% and 15% respectively, due to the lower reactivity of ketones.

Similarly, **18** (43%) and **16** (10%) were obtained when the reaction was performed with 1,2-epoxycyclohexane as electrophile (entry 6).

Finally, when ethyl chloroformate was used as electrophile, the mixture of epimers in C7 **19** (54%) was obtained as the only isolable compounds, in this case ^1^H- and ^13^C-NMR spectra showed signals corresponding to the mixture. Nevertheless, this could be an interesting triorthogonal derivative to achieve the objectives of the project.

The results obtained indicate that this is an effective methodology capable of supporting the addition of different electrophiles, which allows the incorporation of different potential functionalities in the synthesis of interesting organic molecules and natural products.

### 2.4. Application to the Synthesis of the Iridoid Natural Product Core

**Scheme 7:**
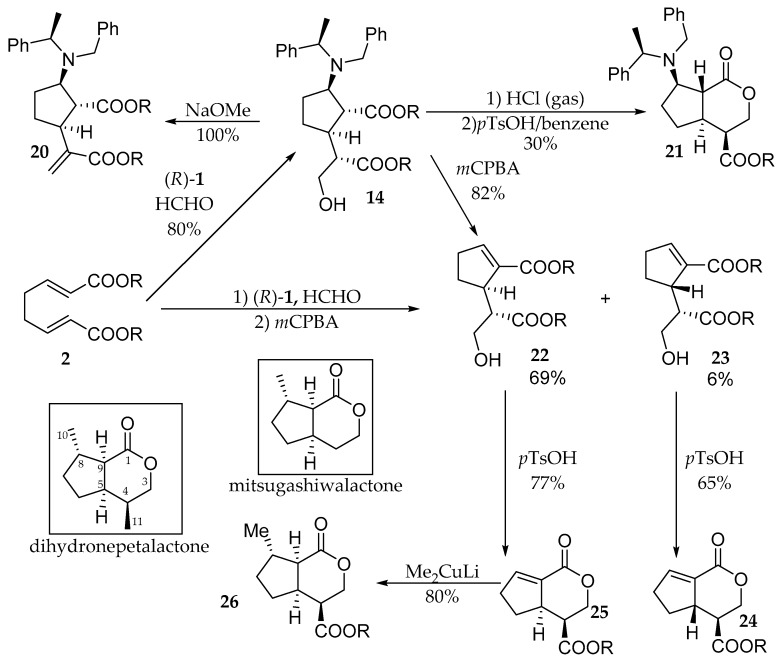
Synthesis of iridoid derivative **26.**

**Figure 4:**
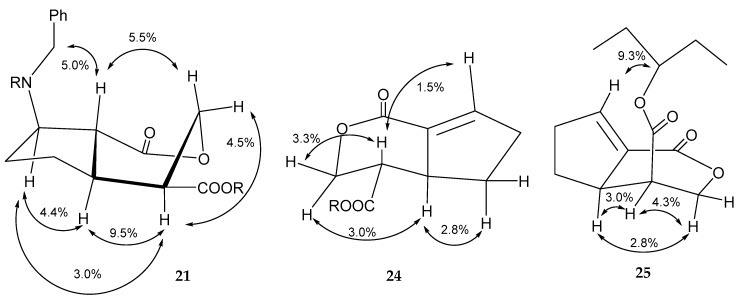
Observed n.O.es for compounds **21**, **24** and **25**.

It was shown in the retrosynthetic Scheme 2 that iridoid natural products are available from intermediate **VII** with cyclopentan[c]pyran skeleton, which is available from intermediate **VIII**, which is the one obtained in the domino reaction and subsequent tandem addition of formaldehyde (compound **14**). Key steps towards achieving the objective are: cyclization reaction and substitution of the amine with a methyl group. The first approach was to try cyclization in an acidic medium (*p*TsOH) but little transformation (5%) was observed, then, when basic conditions were used, either with NaOMe or NaH, the dehydration product **20** was obtained quantitatively (Scheme 7), which is an interesting synthon within this methodology. As from our experience the lone pair electrons in amine group prevents reactivity in acidic media, we treated the solution with HCl (g) prior to the *p*TsOH acid addition to obtain in 30% yield the lactone **21**, the precursor of iridomyrmecin (Figure 1). COSY 2D correlation experiments and significant n.O.es (Figure 4) have allowed us to establish both the absolute stereochemistry and the conformation of the molecule. Relevant n.O.es are H5 with H9 and H4 and this last one with H3*α,* showing that these four hydrogens are *cis*. Also, the H9 with H3*β* n.O.e is due to a boat conformation for the *δ*-lactone with the ester group equatorial and H4 axial (3.08 ppm, ddd, *J* = 9.8, 9.8 and 7.5 Hz), which explains its coupling constants and those of H3*α* and H3*β*. The (4*S*,5*S*,8*R*,9*R*,1′*R*) stereochemistry assigned for **21** matches those from **14**.

Taking into account that the lone pair electrons in the amine complicate the cyclization reaction, the stereospecific *syn* concerted elimination reaction of Cope was tried first, so when **14** was treated with *m*CPBA, compound **22** was obtained in 82% isolated yield. When the reaction was carried out directly from the diunsaturated diester **2** and after the addition of the (*R*)-**1** amide and formaldehyde, and the reaction crude mixture was treated directly with *m*CPBA, **22** and **23** were separated by column chromatography with 69% and 6% yield, respectively. Then, cyclization of each derivative was performed with *p*TsOH, and **24** and **25** were obtained in 77% and 65% yield respectively. The observed n.O.e (Figure 4) sets the stereochemistry for these compounds, which are C4 epimers [36] and therefore corroborates those deduced from adduct **14**.

Finally, the methylation of **25** was performed with Me_2_CuLi, so **26** was obtained stereoselectively in 80% yield [46]. The shift of the methyl group at 1.20 ppm is consistent with the one described for mitsugashiwalactone [47] at 1.18 ppm, which presents this stereochemistry, compared to onikalactone [48] at 0.99 ppm with the opposite and it is the compound synthesized by Tanahashi et al. which proves the stereochemistry of natural secoiridoid glucoside from *Fraxinus americana L.* (Figure 1: **I**) [23].

## 3. Conclusions

In summary, the total asymmetric synthesis of the iridoid **26**, with a methylcyclopentan[c]pyran skeleton, has been carried out in four reaction steps from the affordable octadiendioate **2** and chiral lithium amide (*R*)-**1** and with an overall yield of 40%. This compound is a very advanced analogue of natural products such as: dihydronepetalactone, deoxyloganin or mitsugashiwalactone (by decarboxylation) [42] and it is identical to the cyclopentane system of a new secoiridoid isolated from *Fraxinus americana L*., so a very effective asymmetric synthesis methodology of iridoidal natural product is developed, providing access to a great diversity of these derivatives. The key step of the synthesis is the initial multicomponent domino reaction, where six sterocenters are developed in one pot, the initial four in the domino reaction of Michael addition and intramolecular cyclization and the subsequent two by aldol condensation. Importantly, the analogue series of reactions using the enantiomer of lithium amide (*S*)-**1** in the domino reaction step will allow simple access to the corresponding enantiomers of the aforementioned compounds. Spectroscopic analysis, including homo and heteronuclear two-dimensional correlation experiments have allowed full data assignment and n.O.e and ROESY experiments were performed to determine stereochemistry, especially in derivative products such as dioxolane **9**, or in the case of formaldehyde as electrophile by obtaining the *δ*-lactones: **21**, **24** and **25**. A mechanistic proposal for the reaction course has been postulated and the application to the synthesis of such important natural derivatives as: iridomyrmecin, mitsugashiwalactone and dihydronepetalactone, is underway in our laboratory. 

## 4. Materials and Methods

### 4.1. General Information

Nuclear Magnetic Resonance: Both proton NMR (^1^H-NMR) and carbon NMR (^13^C-NMR), as well as 2D homo and heteronuclear experiments, were recorded in deuterated solvents, on spectrometers working on 200 MHz or 400 MHZ for proton and 50 MHz or 100 MHz for carbon, and are shown in the Supplementary Materials. Data shown below is represented as follows: chemical shift, multiplicity, coupling constant, integral and assignment. -Mass Spectroscopy: Mass spectra was recorded using Atmospheric Pressure Chemical Ionization (APCI).

Rotatory Power: Rotatory power data was recorded using CHCl_3_ as solvent and sodium D line as polarized light ray.

Infrared Spectrometry: IR data was recorded using liquid IR spectrometer and NaCl crystal as supporting material.

Column chromatography: Silica column chromatography was performed using silica gel 60A (0.060-0.200 mm).

### 4.2. General Procedure for the Synthesis of Compounds ***3***–***11***

*n*-Buli in THF (1.6 M, 1.0 mL,1.60 mmol) was added under Ar atmosphere and at –78 °C to a solution of (*R*)-**1**, (357 mg, 1.69 mmol) in THF (7.0 mL). After 50 min of reaction, a solution of **2** (238 mg, 0.77 mmol) in THF (2.5 mL) was added. After 1 h, PhCHO (2.7 mL) was added and the reaction mixture was stirred until it reached room temperature. Then, a saturated solution of NH_4_Cl (6 mL) was added. The reaction mixture was dissolved in EtOAc and washed with H_2_O, brine and 10% aqueous Na_2_S_2_O_3_ solution. Then, the mixture was dried with Na_2_SO_4_ and, after being filtered, the solvent was evaporated. The resulting mixture (4.41 g) was chromatographed and the desired compounds eluted with hexane/EtOAc 95:5. Yield: 141 mg (23% yield) of **3** and 217 mg (45% yield) of **4**.

Pentan-3-yl (1R,2R,5S)-2-(benzyl((R)-1-phenylethyl)amino)-5-((1S,2R)-1-hydroxy-3-oxo-3-(pentan-3-yloxy) -1-phenylpropan-2-yl)cyclopentane-1-carboxylate (**3**): [α]_D_^26^= +5.11(CHCl_3_, c= 0.89). IR (cm^−1^): 3495, 2969, 1723, 1460, 748, 698. H.R.M.S.: calcd for C_40_H_53_NO_5_: 627.3924; found: 627.3895. ^1^H-NMR δ (ppm) (400 MHz, CDCl_3_): 0.40 (3H, t, *J* = 7.3 Hz, CH(CH_2_CH_3_)_2_), 0.78 (3H, t, *J* = 7.3 Hz, CH(CH_2_CH_3_)_2_), 0.80 (3H, t, *J* = 7.3 Hz, CH(CH_2_CH_3_)_2_), 0.95 (3H, t, *J* = 7.3 Hz, CH(CH_2_CH_3_)_2_), 1.32 (3H, d, *J* = 7.0 Hz, H-2′), 1.55 (2H, m, H-4), 1.71 (2H, m, H-3), 2.62 (1H, m, H-1), 2.64 (1H, m, H-5), 2.71 (1H, m, H-7), 3.59 (1H, q, *J* = 7.6 Hz, H-2), 3.68 (1H, d, *J* = 14.6 Hz, H-1′’A), 3.72 (1H, d, *J* = 14.6 Hz, H-1′’B), 3.86 (1H, q, *J* = 7.0 Hz, H-1′), 3.92 (1H, d, *J* = 9.1 Hz, OH), 4.57 (2H, quintet, *J* = 6.1 Hz, CH(CH_2_CH_3_)_2 ×_ 2), 4.87 (1H, dd, *J* = 9.1 and 3.7 Hz, H-1′’’), 7.23-7.29 (15H, Ar). ^13^C-NMR δ (ppm) (CDCl_3_): 8.6 (CH_3_, CH(CH_2_CH_3_)_2_), 9.2 (CH_3_, CH(CH_2_CH_3_)_2_x2), 9.5 (CH_3_, CH(CH_2_CH_3_)_2_), 15.2, (CH_3_, C-2′), 25.3 (CH_2_, CH(CH_2_CH_3_)_2_x3), 25.6 (CH_2_, CH(CH_2_CH_3_)_2_), 27.3 (CH_2_, C-4), 28.8 (CH_2_, C-3), 41.5 (CH, C-5), 50.0 (CH_2_, C-1′’), 52.4 (CH, C-1), 55.1 (CH, C-7), 57.3 (CH, C-1′), 64.8 (CH, C-2), 72.2 (CH, C-1′’’), 76.5 (CH, CH(CH_2_CH_3_)_2_), 77.6 (CH, CH(CH_2_CH_3_)_2_), 125.6 - 128.6 (CHx10, Ar), 141.4 (C, C_ipso_), 142.4 (C, C_ipso_), 143.9 (C, C_ipso_), 174.5 (COOR, C-6), 182,4 (COOR, C-8).

Pentan-3-yl (1R,2R,5S)-2-(benzyl((R)-1-phenylethyl)amino)-5-((1R,2R)-1-hydroxy-3-oxo-3-(pentan-3-yl- oxy)-1-phenylpropan-2-yl)cyclopentane-1-carboxylate (**4**): [α]_D_^26^= +0.90 (CHCl_3_, c= 1.29). H.R.M.S.: calcd for C_40_H_53_NO_5_: 627.3924; found: 627.3864. IR (cm^−1^): 3495, 2969, 1723, 1495, 1169, 912, 748, 698. ^1^H-NMR δ (ppm) (400 MHz, CDCl_3_): 0.32 (3H, t, *J* = 7.5 Hz, CH(CH_2_CH_3_)_2_), 0.73 (3H, t, *J* = 7.5 Hz, CH(CH_2_CH_3_)_2_), 0.98 (3H, t, *J* = 7.5 Hz, CH(CH_2_CH_3_)_2_), 1.00 (3H, t, *J* = 7.5 Hz, CH(CH_2_CH_3_)_2_), 1.08 (2H, m, CH(CH_2_CH_3_)_2_), 1.28 (3H, d, *J* = 6.8 Hz, H-2′), 1.35 (2H, m, CH(CH_2_CH_3_)_2_), 1.55 (1H, m, H-4A), 1.64 (2H, m, CH(CH_2_CH_3_)_2_), 1.65 (2H, m, CH(CH_2_CH_3_)_2_), 1.75 (2H, m, H-3), 1.85 (1H, m, H-4B), 2.74 (2H, m, H-5), 2.87 (1H, dd, *J* = 10.0 and 3.8 Hz, H-7), 3.04 (1H, d, *J* = 10.0 Hz, OH), 3.23 (1H, dd, *J* = 10.1 and 9.9 Hz, H-1), 3.56 (1H, ddd, *J* = 9.9, 8.2 and 8.2 Hz, H-2), 3.67 (1H, d, *J* = 14.4 Hz, H-1′’A), 3.85 (1H, d, *J* = 14.4 Hz, H-1′’B), 3.90 (1H, q, *J* = 6.8 Hz, H-1′), 4.44 (1H, quintet, *J* = 6.0 Hz, CH(CH_2_CH_3_)_2_,), 4.64 (1H, dd, *J* = 10.0 and 3.5 Hz, H-1′’’), 4.67 (1H, quintet, *J* = 6.0 Hz, CH(CH_2_CH_3_)_2_), 7.1 - 7.6 (15H, Ar-H). ^13^C-NMR δ (ppm) (CDCl_3_): 8.6 (CH_3_, CH(CH_2_CH_3_)_2_), 9.0 (CH_3_, CH(CH_2_CH_3_)_2_), 9.2 (CH_3_, CH(CH_2_CH_3_)_2_) 9.4 (CH_3_, CH(CH_2_CH_3_)_2_), 15,1 (CH_3_, C-2′), 24.5 (CH_2_, CH(CH_2_CH_3_)_2_), 25.0 (CH_2_, CH(CH_2_CH_3_)_2_), 25.2 (CH_2_, CH(CH_2_CH_3_)_2_), 25.4 (CH_2_, CH(CH_2_CH_3_)_2_), 26.6 (CH_2_, C-3), 28.8 (CH_2_, C-4), 40.0 (CH, C-5), 49.9 (CH_2_, C-1′’), 50.0 (CH, C-1), 56.5 (CH_2_, C-7), 57.2 (CH, C- 1′), 65.1 (CH, C-2), 73.3 (CH, C-1′’’), 77.1 (CH, CH(CH_2_CH_3_)_2_), 77.4 (CH, CH(CH_2_CH_3_)_2_), 126.5 - 128.8 (CHx15, Ar), 141.3 (C, C_ipso_), 141.6 (C, C_ipso_), 144.1 (C, C_ipso_), 172.0 (COOR, C-8), 177.4 (COOR, C-6).

To a solution of **3** (121 mg, 0.193 mmol) in CH_2_Cl_2_ (3 mL) under an Ar atmosphere and at –78 °C, 1.5 M DIBALH (0.4 mL, 0.58 mmol) was added. After 2 h of reaction, H_2_O (1.5 mL) was added and the resulting mixture was washed in an Erlenmeyer flask with NaHCO_3_ and Na_2_SO_4_ (3 g of each) for 5 h. After being filtered, the solvent was evaporated and 101 mg of crude product were obtained. The desired compounds were subjected to chromatography eluting with hexane/EtOAc 9:1 to afford 59 mg of **5** (yield 57%), 25 mg of **6** (yield 24%) and 8 mg of **7** (yield 9%).

*Pentan-3-yl (2R,3R)-2-((1S,2R,3R)-3-(benzyl((R)-1-phenylethyl)amino)-2-(hydroxymethyl)cyclopentyl)-3-hydroxy-3-phenylpropanoate* (**5**): IR (cm^−1^): 3368, 2969, 1717, 1456, 1169, 1028, 750, 698. H.R.M.S.: calcd for C_35_H_45_NO_4_: 543.3349; found: 543.3398. ^1^H-NMR *δ* (ppm) (400 MHz, CDCl_3_): 0.37 (3H, t, *J* = 7.5 Hz, CH(CH_2_C*H*_3_)_2_), 0.78 (3H, t, *J* = 7.5 Hz, CH(CH_2_C*H*_3_)_2_), 1.13 (3H, m, CH(CH_2_C*H*_3_)_2_), 1.38 (3H, d, *J* = 6.8 Hz, H-2′), 1.35 (3H, m, CH(CH_2_C*H*_3_)_2_), 2.24 (1H, m, H-1), 2.24 (1H, m, H-5), 2.84 (1H, m, H-7), 2.84 (1H, m, H-2), 3.44 (1H, m, H-6A), 3.55 (1H, m, H-6B), 3.58 (1H, d, *J* = 14.4 Hz, H-1′’A), 3.91 (1H, d, *J* = 14.4 Hz, H-1′’B), 3.94 (1H, q, *J* = 6.8 Hz, H-1′), 4.70 (1H, quintet, *J* = 8 Hz, C*H*(CH_2_CH_3_)_2_), 4.96 (1H, d, *J* = 11.6 Hz, H-1′’’), 7.22-7.40 (15 H, Ar-H). ^13^C-NMR *δ* (ppm) (CDCl_3_): 8.6 (CH_3_, CH(CH_2_CH_3_)_2_), 9.7 (CH_3_, CH(CH_2_CH_3_)_2_), 12.4 (CH_3_, C-2′), 25.3 (CH_2_, CH(CH_2_CH_3_)_2_), 25.4 (CH_2_, CH(CH_2_CH_3_)_2_), 25.7 (CH_2_, C-3), 28.5 (CH_2_, C-4), 36.1 (CH, C-5), 45.1 (CH, C-1), 50.1 (CH_2_, C-1′’), 55.4 (CH, C-1′), 57.2 (CH, C-7), 60.7 (CH, C-2), 64.7 (CH, C-6), 73.3 (CH, C-1′’’), 76.8 (CH, CH(CH_2_CH_3_)_2_), 126.8 - 128.9 (CHx15, Ar), 140.8 (C, C*_ipso_*), 142.1 (C, C*_ipso_*), 144.0 (C, C*_ipso_*), 173.0 (COOR, C-8).

Pentan-3-yl (1R,2R,5R)-2-(benzyl((R)-1-phenylethyl)amino)-5-((1R,2S)-1,3-dihydroxy-1-phenyl- propan-2-yl)-cyclopentane-1-carboxylate (**6**): [α]_D_^26^ = -4.5 (CHCl_3_, c = 0.79). IR (cm^−1^): 3422, 2969, 1717, 1486, 1028, 750, 667. H.R.M.S.: calcd for C_35_H_45_NO_4_: 559.3662; found: 543.3293. ^1^H-NMR δ (ppm) (400 MHz, CDCl_3_): 0.83 (3H, t, *J* = 7.6 Hz, CH(CH_2_CH_3_)_2_), 0.89 (3H, t, *J* = 7.6 Hz, CH(CH_2_CH_3_)_2_), 1.26 (1H, m, H-4A), 1.31 (4H, m, CH(CH_2_CH_3_)_2_x2), 1.34 (3H, d, *J* = 6.8 Hz, H-2′), 1.65 (1H, m, H-3A), 1.75 (1H, m, H-3B), 1.80 (1H, m, H-4B), 1.99 (1H, m, H-7), 2.27 (1H, m, H-5), 2.73 (1H, t, *J* = 9.1 Hz, H-1), 3.51 (1H, d, *J* = 4.5 Hz, H-8A), 3.55 (1H, m, H-2), 3.69 (1H, d, *J* = 14.6, H-1′’A), 3.77 (1H, d, *J* = 14.6 Hz, H-1′’B), 3.82 (1H, m, H-8B), 3.90 (1H, q, *J* = 6.8 Hz, H-1′), 4.58 (1H, quintet, *J* = 5.9 Hz, CH(CH_2_CH_3_)_2_), 4.86 (1H, d, *J* = 6.5 Hz, H-1′’B), 7.2-7.6 (15H, Ar-H). ^13^C-NMR δ (ppm) (CDCl_3_): 9.0 (CH_3_, CH(CH_2_CH_3_)_2_), 9.2 (CH_3_, CH(CH_2_CH_3_)_2_), 15.1 (CH_3_, C-2′), 24.8 (CH_2_, CH(CH_2_CH_3_)_2_), 25.1 (CH_2_, CH(CH_2_CH_3_)_2_), 27.1 (CH_2_, C-3), 30.3 (CH_2_, C-4), 39.8 (CH, C-5), 50.0 (CH_2_, C-1′’), 50.6 (CH, C-7), 53.0 (CH, C-1), 58.0 (CH, C-1′), 63.4 (CH_2_, C-8), 65.1 (CH, C-2),75.6 (CH, C-1′’’), 77.0 (CH, CH(CH_2_CH_3_)_2_), 125.3 - 128.7 (CHx15, Ar), 141.5 (C, C_ipso_), 142.7 (C, C_ipso_), 143.9 (C, C_ipso_), 176.6 (COOR, C-6).

*(1R,2S)-2-((1S,2R,3R)-3-(benzyl((R)-1-phenylethyl)amino)-2-(hydroxymethyl)cyclopentyl)-1-phenyl-propane-1,3-diol* (**7**): IR (cm^−1^): 3339, 2940, 1495, 1028, 737, 700. H.R.M.S.: calcd for C_30_H_37_NO_3_: 459.2773; found: 459.2735 ^1^H-NMR δ (ppm) (200 MHz, CDCl_3_): 1.26 (1H, m, H-7), 1.36 (3H, d, *J* = 6.8 Hz, H-2′), 1.46-1.87 (2H, m, H-3; 1H, m, H-4A, 1H, m, H-5) 1.92 (1H, m, H-4B), 1.95 (1H, m, H-1), 2.83 (1H, q, *J* = 8 Hz, H-2), 3.28 (1H, m, H-6A), 3.36 (1H, m, H-6B), 3.60 (2H, m, H-8), 3.62 (1H, d, *J* = 7.0 Hz, H-1′’B), 3.90 (1H, q, *J* = 6.8 Hz, H-1′), 3.94 (1H, d, *J* = 7.0 Hz, H -1′’A), 4.94 (1H, d, *J* = 5.8 Hz, H-1′’’), 7.21-7.38 (15 H, Ar-H). ^13^C-NMR δ (ppm) (CDCl_3_): 12.9 (CH_3_, C-2′), 25.9 (CH_2_, C-3), 29.0 (CH_2_, C-4), 36.1 (CH, C-5), 46.8 (CH, C-1), 50.3 (CH_2_, C-1′’), 56.5 (CH, C-1′), 50.7 (CH, C-7), 62.4 (CH, C-2), 62.8 (CH_2_, C-8), 66.0 (CH2, C-6), 75.3 (CH, C-1′’’), 126.5 – 129.3 (CHx15, Ar), 140.7 (C, C*_ipso_*), 143.4 (C, C*_ipso_*), 143.6 (C, C*_ipso_*).

Also, to a solution of **3** (147 mg, 0.235 mmol) in dry ether (5 mL) at 0 °C, LiAlH_4_ (24 mg) was added and, after 45 min of stirring, dry ether saturated with water (1 mL) was added. The reaction mixture was filtered through Celite-silica and washed with ether and CHCl_3_. The solvent was evaporated and 101 mg of crude product were obtained and chromatographed. 20 mg of **5** (yield 16%), 39 mg of **6** (yield 31%) and 16 mg of **7** (yield 15%). 

To a solution of **3** + **4** (2:3 ratio) (86 mg, 0.137 mmol) in DCM (3 mL), 1.5 M DIBALH (1.12 mL, 1.680 mmol) was added at –78 °C under an Ar atmosphere. After 1 h, the reaction flask was allowed to reach room temperature and H_2_O (1.5 mL) was added. Then, the reaction mixture was placed in an Erlenmeyer flask with ether, NaHCO_3_ (3 g) and Na_2_SO_4_ (3 g) and the resulting mixture left stirring for 5 h. Then, it was chromatographed and the desired products were eluted with hexane/EtOAc 8:2 to give 26 mg of **7** (yield 38%) and 18 mg of **8** (yield 27%).

*(1S,2S)-2-((1S,2R,3R)-3-(benzyl((R)-1-phenylethyl)amino)-2-(hydroxymethyl)cyclopentyl)-1-phenyl- propane-1,3-diol* (**8**): I.R. (cm^−1^): 3341, 2930, 1495, 1059, 750, 700. ^1^H-NMR *δ* (ppm) (400 MHz, CDCl_3_): 1.25 (1H, H-7), 1.39 (3H, d, *J* = 6.9 Hz, H-2′), 1.44 (1H, m, H-5), 1.67 (1H, m, H-4A), 1.74 (2H, m, H-3), 1.77 (1H, m, H-5), 1.86 (1H, m, H-4B), 2.11 (1H, m, H-1), 2.83 (1H, q, *J* = 8 Hz, H-2), 3.33 (2H, m, H-6), 3.63 (1H, d, *J* = 13.8 Hz, H-1′’A), 3.64 (2H, m, H-8), 3.78 (1H, d, *J* = 13.8 Hz, H-1′’B), 3.92 (1H, q, *J* = 6.9 Hz, H-1′), 5.00 (1H, d, *J* = 3.1 Hz, H-1′’’), 7.21-7.40 (15 H, Ar-H). ^13^C-NMR *δ* (ppm) (CDCl_3_): 13.2 (CH_3_, C-2′), 25.9 (CH_2_, C-3), 28.8 (CH_2_, C-4), 36.5 (CH, C-5), 47.3 (CH, C-1), 50.7 (CH_2_, C-1′’), 56.5 (CH, C-1′), 50.1 (CH, C-7), 62.3 (CH, C-2), 60.9 (CH_2_, C-8), 66.0 (CH_2_, C-6), 76.5 (CH, C-1′’’), 126.6 –129.1 (CHx15, Ar), 140.8 (C, C*_ipso_*), 143.9 (C, C*_ipso_*), 144.8 (C, C*_ipso_*).

To a solution of **7** (22 mg, 0.048 mmol) in acetone (5 mL), a catalytic amount of camphorsulfonic acid (CSA) and 2,2-DMP (5 mL) were added. The reaction was heated up to reflux for 7 h. The reaction mixture was solved in ether and washed with NaHCO_3_ saturated solution, brine and H_2_O. The resulting solution was dried with Na_2_SO_4_ and, after being filtered, 42 mg of crude product were obtained and chromatographed. The desired compound was eluted with hexane/EtOAc 95:5, and 12 mg of **9** were obtained (yield 65%).

*((1R,2R,5S)-2-(benzyl((R)-1-phenylethyl)amino)-5-((4R,5S)-2,2-dimethyl-4-phenyl-1,3-dioxan-5-yl)cyclo- pentyl)methanol* (**9**): IR (cm^−1^): 3332, 2938, 1451, 1200, 739, 700. H.R.M.S.: calcd for C_33_H_41_NO_3_: 499.3086; found: 499.3044. ^1^H-NMR *δ* (ppm) (400 MHz, CDCl_3_): 0.22 (1H, m, H-4A), 0.88 (1H, m, H- 5), 1.17 (1H, m, H-4B), 1.27 (3H, d, *J* = 6.8 Hz, H-2′), 1.28-1.40 (1H, m, H-3A), 1.47 (3H, s, (CH_3_)_2_), 1.49 (3H, s, (CH_3_)_2_), 1.57 (1H, m, H-7), 1.68 (1H, m, H-3B), 1.68 (1H, m, H-1), 2.71 (1H, q, *J* = 8, H-2), 3.08 (1H, dd, *J* = 10.5 and 6.5 Hz, H-6A), 3.47 (1H, d, *J* = 13.5 Hz, H-1′’A), 3.62 (1H, dd, *J* = 10.5 and 2.4 Hz, H-6B), 3.69 (1H, d, *J* = 13.5 Hz, H-1′’B), 3.85 (1H, q, *J* = 6.8 Hz, H-1′), 3.93 (1H, d, *J* = 12.1 Hz, H-8A), 4.17 (1H, dd, *J* = 12.1 and 2.9 Hz, H-8B), 5.22 (1H, d, *J* = 2 Hz, H-1′’’), 7.1-7.6 (15H, Ar-H). ^13^C-NMR *δ* (ppm) (CDCl_3_): 12.6 (CH_3_, C-2′), 18.9 (CH, (CH_3_)_2_), 25.9 (CH_2_, C-3), 29.5 (CH, (CH_3_)_2_), 30.1 (CH_2_, C-4), 35.2 (CH, C-5), 43.2 (CH, C-1), 46.6 (CH, C-7), 49.6 (CH, C-1′’), 56.0 (CH, C-1′), 62.5 (CH, C-2), 64.2 (CH_2_, C-8), 66.0 (CH_2_, C-6), 73.4 (CH, C-1′’’), 99.0 (CH, (CH_3_)_2_), 125.6 –129.2 (CHx15, Ar), 140.0 (C, C*_ipso_*), 141.0 (C, C*_ipso_*), 144.2 (C, C*_ipso_*),143.4 (C, C*_ipso_*).

To a solution of **4** (25 mg, 0.04 mmol) in CH_2_Cl_2_ (3.5 mL), molecular sieves (11 mg) and TPAP (1 mg, 0.004 mmol) were added. After 3 h of stirring, the resulting mixture was filtered through Celite-silica with CH_2_Cl_2_ and, after solvent evaporation, 23 mg of **10** (yield 91%) were obtained.

Pentan-3-yl (1R,2R,5S)-2-(benzyl((R)-1-phenylethyl)amino)-5-((R)-1,3-dioxo-1-(pentan-3-yloxy)-3-phenyl-propan-2-yl)cyclopentane-1-carboxylate (**10**): H.R.M.S.: calcd for C_40_H_51_NO_5_: 626.3767; found: 626.3903. ^1^H-NMR δ (ppm) (400 MHz, CDCl_3_): 0.70 (3H, t, *J* = 7.3 Hz, CH(CH_2_CH_3_)_2_), 0.77 (3H, t, *J* = 7.3 Hz, CH(CH_2_CH_3_)_2_), 0.84 (3H, t, *J* = 7.3 Hz, CH(CH_2_CH_3_)_2_), 0.92 (3H, t, *J* = 7.3 Hz, CH(CH_2_CH_3_)_2_), 1.25 (2H, m, CH(CH_2_CH_3_)_2_), 1.31 (3H, d, *J* = 6.9 Hz, H-2′) 1.41 (2H, m, CH(CH_2_CH_3_)_2_), 1.45 (2H, m, CH(CH_2_CH_3_)_2_), 1.52 (1H, m, H-4A), 1.64 (2H, m, CH(CH_2_CH_3_)_2_), 1.75 (2H, m, H-3), 1.85 (1H, m, H-4B), 2.77 (1H, m, H-5), 2.92 (1H, dd, *J* = 8.6 and 7.3 Hz, H-1), 3.62 (1H, m, H-2), 3.89 (1H, q, *J* = 6.9 Hz, H-1′), 4.3 (1H, d, *J* = 10 Hz, H-7), 3.90 (1H, d, *J* = 14.5 Hz, H-1′’A), 3.93 (1H, d, *J* = 14.5 Hz H-1′’B), 4.59 (1H, quintet, *J* = 5.7 Hz, CH(CH_2_CH_3_)_2_), 4.69 (1H, quintet, *J* = 5.7 Hz, CH(CH_2_CH_3_)_2_), 7.1-7.6 (13H, m, Ar-H), 7.9 (2H, d, *J* = 7.3 Hz). ^13^C-NMR δ (ppm) (CDCl_3_): 9.5 (CH_3_, CH(CH_2_CH_3_)_2_x3), 9.6 (CH_3_, CH(CH_2_CH_3_)_2_) 15.8 (CH_3_, C-2′), 25.5 (CH_2_, CH(CH_2_CH_3_)_2_), 25.6 (CH_2_, CH(CH_2_CH_3_)_2_x2), 26.1 (CH_2_, CH(CH_2_CH_3_)_2_), 27.7 (CH_2_, C-3), 28.8 (CH_2_, C-4), 41.5 (CH, C-5), 52.6 (CH, C-1), 50.3 (CH_2_, C-1′’), 57.0 (CH_2_, C-7), 57.8 (CH, C-1′), 65.3 (CH, C-2), 77.1 (CH, CH(CH_2_CH_3_)_2_), 78.5 (CH, CH(CH_2_CH_3_)_2_), 126.7 - 128.8 (CHx15, Ar), 137.8 (C, C_ipso_), 141.9 (C, C_ipso_), 144.7 (C, C_ipso_), 169.2 (COOR, C-8), 175.1 (COOR, C-6), 194.1 (C, C-1′’’).

To a solution of **4** + **3** (29 mg, 0.045 mmol) in CH_2_Cl_2_ (3 mL) PDC (26 mg, 0.0687 mmol) was added. After 24 h, saturated NH_4_Cl solution (3 mL) was added and the resulting mixture was filtered, dissolved in EtOAc and washed with H_2_O and brine. After drying over Na_2_SO_4_, filteration and solvent evaporated, the resulting 27 mg were chromatographed and the desired compounds eluted with hexane/EtOAc 95:5 to afford 5 mg of **10** (yield 18%) and 13 mg of **11** (yield 49%).

Pentan-3-yl (1R,2S,5R)-2-((R)-1,3-dioxo-1-(pentan-3-yloxy)-3-phenylpropan-2-yl)-5-(N-((R)-1-phenyl-ethyl)benzamido)cyclopentane-1-carboxylate (**11**) IR (cm^−1^): 2969, 1732, 1688, 1456, 1219, 1105, 702. ^1^H-NMR δ (ppm) (400 MHz, CDCl_3_): 0.60-0.93 (12H, m, CH(CH_2_CH_3_)_2_), 1.26 (3H, d, *J* = 7.1 Hz, H-2′), 1.30-1.62 (8H, m, CH(CH_2_CH_3_)_2_), 1.52 –1.99 (4H, m, H-3 and H-4), 2.48 (1H, dd, *J* = 8.6 and 7.3 Hz, H-1), 3.15 (1H, m, H-5), 3.17 (1H, m, H-7), 3.89 (1H, q, *J* = 7.1 Hz, H-1′), 4.45 (1H, dd, *J* = 13.3 and 7.3 Hz, H-2), 4.71 (2H, m, CH(CH_2_CH_3_)_2_x2), 7.1-7.6 (11H, m, Ar-H), 7.9 (4H, m, Ar-H). ^13^C-NMR δ (ppm) (CDCl_3_): 9.4 (CH_3_, CH(CH_2_CH_3_)_2_) 9.5 (CH_3_, CH(CH_2_CH_3_)_2_x2), 9.7 (CH_3_, CH(CH_2_CH_3_)_2_), 22.2 (CH_3_, C-2′), 25.7 (CH_2_, CH(CH_2_CH_3_)_2_), 26.0 (CH_2_, CH(CH_2_CH_3_)_2_), 26.1 (CH_2_, CH(CH_2_CH_3_)_2_), 26.2 (CH_2_, CH(CH_2_CH_3_)_2_), 29.5 (CH_2_, C-3), 31.1 (CH_2_, C-4), 43.3 (CH, C-5), 53.1 (CH, C-1), 57.9 (CH_2_, C-7), 58.1 (CH, C-1′), 61.5 (CH, C-2), 78.1 (CH, CH(CH_2_CH_3_)_2_), 78.5 (CH, CH(CH_2_CH_3_)_2_), 128.8–129.5 (CHx15, Ar), 133.7 (C, C_ipso_), 133.8 (C, C_ipso_), 136.6 (C, C_ipso_), 168.9 (COOR, C-8), 173.7 (COOR, C-6), 194.7 (C, C-1′’’), 195.3 (C, C-1′’).

### 4.3. General Tandem Domino Michael Addition Procedure: Compounds ***12*–*20***

To a solution of (*R*)-**1** (2.3 equivalents) in THF at –78 °C under Ar atmosphere, 1.6 M *n*-BuLi (2.2 equivalents) was added. After 50 min of reaction, **2** (1 equivalent) was added and, after 1 h of reaction, an electrophile (1.5 equivalents) in THF was added. Then, after 3 h, the reaction was quenched with NH_4_Cl. The resulting reaction mixture was dissolved with EtOAc and washed with 10% aqueous citric acid solution, H_2_O and brine. After being dried over Na_2_SO_4_ and filtered, the solvent was evaporated and the reaction mixture was chromatographed.

When using benzaldehyde as electrophile, 1.34 g of reaction mixture were obtained and chromatographed to give 63 mg (yield 30%) of **12** and 57 mg (yield 27%) of **13**.

Pentan-3-yl (1R,2R,5S)-2-(benzyl((R)-1-phenylethyl)amino)-5-((2R,3R,E)-3-hydroxy-1-oxo-1-(pentan-3-yl-oxy)-5-phenylpent-4-en-2-yl)cyclopentane-1-carboxylate (**12**): IR (cm^−1^): 3503, 2967, 1724, 1458, 1177, 1105, 698. H.R.M.S.: calcd for C_42_H_55_NO_5_: 653.4080; found: 653.4150. ^1^H-NMR δ (ppm) (200 MHz, CDCl_3_): 0.60-0.96 (9H, m, CH(CH_2_CH_3_)_2_), 1.12 (3H, m, CH(CH_2_CH_3_)_2_), 1.32 (3H, d, *J* = 6.8 Hz, H-2′), 1.35-1.58 (8H, m, CH(CH_2_CH_3_)_2_), 1.55-1.90 (4H, m, H-3 and H-4), 2.53 (1H, d, *J* = 7.5, H-7), 2.61 (1H, m, H-5), 2.82 (1H, t, *J* = 6Hz, H-1), 3.35 (1H, d, *J* = 8.4 Hz, OH), 3.63 (1H, d, *J* = 4.2 Hz, H-1′’A), 3.77 (1H, ddd, H-2), 3.85 (1H, q, *J* = 6.8 Hz, H-1′), 3.87 (1H, d, *J* = 4.2 Hz, H-1′’B), 4.42 (1H, m, H-1′’’), 4.55 (1H, q, *J* = 6.0, CH(CH_2_CH_3_)_2_), 4.71 (1H, quintet, *J* = 6.0 Hz, CH(CH_2_CH_3_)_2_), 6.18 (1H, dd, *J* = 14.2 and 5.2 Hz, H-2′’’), 6.60 (1H, d, *J* = 17.4 Hz, H-3′’’), 7.1-7.6 (10H, Ar-H). ^13^C-NMR δ (ppm) (CDCl_3_): 9.4 (CH_3_, CH(CH_2_CH_3_)_2_x2), 9.8 (CH_3_, CH(CH_2_CH_3_)_2_x2), 15.6 (CH_3_, C-2′), 25.5 (CH_2_, CH(CH_2_CH_3_)_2_), 25.9 (CH_2_, CH(CH_2_CH_3_)_2_), 26.1 (CH_2,_ CH(CH_2_CH_3_)_2_), 27.5 (CH_2_, CH(CH_2_CH_3_)_2_), 28.8 (CH_2_, C-3), 29.1 (CH_2_, C-4), 41.5 (CH, C-5), 50.3 (CH_2_, C-1′’), 52.5 (CH, C-1), 53.8 (CH_2_, C-7), 57.5 (CH, C-1′), 65.1 (CH, C-2), 71.1 (CH, C-1′’’), 76.8 (CH, CH(CH_2_CH_3_)_2_), 77.1 (CH, CH(CH_2_CH_3_)_2_), 126.6-128.8 (CHx15, Ar), 130.4 (CH, Ph-CH=CH-), 130.9 (CH, Ph-CH=CH-), 136.7 (C, C_ipso_), 141.6 (C, C_ipso_), 144.3 (C, C_ipso_), 174.2 (COOR, C-8), 174.9 (COOR, C-6).

Pentan-3-yl (1R,2R,5S)-2-(benzyl((R)-1-phenylethyl)amino)-5-((2R,3S,E)-3-hydroxy-1-oxo-1-(pentan-3-yl-oxy)-5-phenylpent-4-en-2-yl)cyclopentane-1-carboxylate (**13**): IR (cm^−1^): 3510, 2969, 1719, 1458, 1105, 698. ^1^H-NMR δ (ppm) (200 MHz, CDCl_3_): 0.63 (3H, m, CH(CH_2_CH_3_)_2_), 0.73-1.01 (9H, m, CH(CH_2_CH_3_)_2_), 1.27 (3H, d, *J* = 6.8 Hz, H-2′), 1.38-1.95 (8H, m, CH(CH_2_CH_3_)_2_; 2H, m, H-3; 2H, m, H-4), 2.60 (1H, m, H-5), 2.63 (1H, d, *J* = 8 Hz, H-7), 2.93 (1H, d, *J* = 4.5 Hz, OH), 3.10 (1H, t, *J* = 6Hz, H-1), 3.63 (1H, d, *J* = 4.2 Hz, H-1′’A), 3.77 (1H, m, H-2), 3.85 (1H, q, *J* = 6.8 Hz, H-1′), 3.87 (1H, d, *J* = 6.8 Hz, H-1′’B), 4.31 (1H, m, H-1′’’), 4.64 (2H, m, CH(CH_2_CH_3_)_2_x2), 6.18 (1H, dd, H-2′’’), 6.53 (1H, d, H-3′’’), 7.1-7.6 (10H, Ar-H).

When using formaldehyde as electrophile, 155 mg of crude product were obtained and chromatographed to give after elution with hexane/EtOAc 95:5 140 mg (yield 80%) of **14**.

Pentan-3-yl (1R,2R,5S)-2-(benzyl((R)-1-phenylethyl)amino)-5-((S)-3-hydroxy-1-oxo-1-(pentan-3-yloxy)-propan-2-yl)cyclopentane-1-carboxylate (**14**): IR (cm^−1^): 3488, 2969, 1726, 1456, 1105, 748, 700. H.R.M.S.: calcd for C_34_H_49_NO_5_: 551.3611; found: 551.3573. ^1^H-NMR δ (ppm) (400 MHz, CDCl_3_): 0.83 (9H, m, CH(CH_2_CH_3_)_2_), 0.96 (3H, t, *J* = 7.5 Hz, CH(CH_2_CH_3_)_2_), 1.30 (3H, d, *J* = 6.8 Hz, H-2′), 1.4-1.6 (8H, m, CH(CH_2_CH_3_)_2_), 1.63 (2H, m, H-3), 1.72 (2H, m, H-4), 2.46 (1H, m, H-5), 2.62 (1H, d, *J* = 7.5, H-7), 2.76 (1H, t, *J* = 9.5 Hz, H-1), 3.60 (1H, m, H-1′’’A), 3.64 (1H, m, H-2), 3.65 (1H, m, H-1′’’B),3.71 (1H, d, *J* = 11.1 Hz, H-1′’A), 3.64 (1H, m, H-2), 3.80 (1H, d, *J* = 11.1 Hz, H-1′’B), 3.85 (1H, q, *J* = 6.8 Hz, H-1′), 4.61 (1H, quintet, *J* = 6.0, CH(CH_2_CH_3_)_2_), 4.75 (1H, quintet, *J* =6 Hz, CH(CH_2_CH_3_)_2_), 7.1-7.6 (10H, Ar-H). ^13^C-NMR δ (ppm) (CDCl_3_): 9.4 (CH_3_, CH(CH_2_CH_3_)_2_), 9.5 (CH_3_, CH(CH_2_CH_3_)_2_), 9.7 (CH_3_, CH(CH_2_CH_3_)_2_), 9.8 (CH_3_, CH(CH_2_CH_3_)_2_), 15.4 (CH_3_, C-2′), 25.4 (CH_2_, CH(CH_2_CH_3_)_2_x2), 25.6 (CH_2_, C-3), 26.4 (CH_2_, CH(CH_2_CH_3_)_2_), 26.6 (CH_2_, CH(CH_2_CH_3_)_2_), 41.1 (CH, C-5), 49.5 (CH, C-1), 50.2 (CH_2_, C-1′’), 51.8 (CH, C-1′), 57.8 (CH, C-7), 61.1 (CH_2_, C-1′’’), 64.5 (CH_2_, C-2), 77.1 (CH, CH(CH_2_CH_3_)_2_), 77.6 (CH, CH(CH_2_CH_3_)_2_), 126.4 –128 (CHx10, Ar), 141.6 (C, C_ipso_), 144.3 (C, C_ipso_), 173.9 (COOR, C–8), 175.3 (COOR, C–6).

When using acetone as electrophile, 160 mg were obtained and chromatographed to obtain 60 mg (yield 27%) of **15** and 100 mg (yield 25%) of **16**.

Pentan-3-yl (1R,2R,5S)-2-(benzyl((R)-1-phenylethyl)amino)-5-((R)-3-hydroxy-3-methyl-1-oxo-1-(pentan-3-yloxy)butan-2-yl)cyclopentane-1-carboxylate (**15**): ^1^H-NMR δ (ppm) (200 MHz, CDCl_3_): 0.8-0.9 (12H, m, CH(CH_2_CH_3_)_2_), 1.17 (3H, s, CH_3_), 1.18 (3H, s, CH_3_), 1.26 (3H, d, *J* = 6.8 Hz, H-2′), 1.41-1.91 (8H, m, CH(CH_2_CH_3_)_2_); 2H, m, H-3; 2H, m, H-4), 2.38 (1H, d, *J* = 5.2 Hz, H-7), 2.63 (1H, m, H-5), 2.83 (1H, t, *J* = 8.8 Hz, H-1), 3.41-3.92 (1H, m, H-1′; 2H, m, H-1′’’; 1H, m, H-2), 4.50 (1H, quintet, *J* = 6 Hz, CH(CH_2_CH_3_)_2_), 4.75 (1H, quintet, *J* = 6 Hz, CH(CH_2_CH_3_)_2_), 7.1-7.6 (10H, Ar-H). ^13^C-NMR δ (ppm) (CDCl_3_): 9.3 (CH_3_, CH(CH_2_CH_3_)_2_x2), 9.8 (CH_3_, CH(CH_2_CH_3_)_2_x2), 14.7 (CH_3_, C-2′), 24.7 (CH_2_, CH(CH_2_CH_3_)_2_x2), 26.0 (CH_2_, CH(CH_2_CH_3_)_2_), 26.5 (CH_2_, C-3), 26.6 (CH_2_, CH(CH_2_CH_3_)_2_), 30.3 (CH_2_, C-4), 39.0 (CH, C-5), 49.5 (CH, C-1), 50.1 (CH_2_, C-1′’), 52.1 (CH, C-1), 57.8 (CH, C-7), 61.1 (CH_2_, C-1′’’), 64.5 (CH_2_, C-2), 77.1 (CH, CH(CH_2_CH_3_)_2_), 77.6 (CH, CH(CH_2_CH_3_)_2_), 126.4 –128 (CHx10, Ar), 141.6 (C, C_ipso_), 144.3 (C, C_ipso_), 173.9 (COOR, C –8), 175.3 (COOR, C–6).

*Pentan-3-yl (1R,2R,5R)-2-(benzyl((R)-1-phenylethyl)amino)-5-(2-oxo-2-(pentan-3-yloxy)ethyl)cyclopentane-1-carboxylate* (**16**): [α]_D_^26^ = -30.1 (CHCl_3_, c = 1.55). IR (cm^−1^): 2969, 1728, 1454, 1170, 974, 698. ^1^H-NMR *δ* (ppm) (400 MHz, CDCl_3_): 0.78 (3H, t, *J* = 7.5 Hz, CH(CH_2_C*H*_3_)_2_), 0.85 (6H, t, *J* = 7.5, CH(CH_2_C*H*_3_)_2_x2), 0.94 (3H, t, *J* = 7.5 Hz, CH(CH_2_C*H*_3_)_2_), 1.28 (3H, d, *J* = 6.8, H-2′), 1.40 - 1.75 (10H, m, CH_2_), 1.75 - 1.85 (2H, m), 2.17 (1H, dd, *J* = 14.8 and 9.6 Hz, H-7A), 2.39 (1H, m, H-5), 2.50 (1H, dd, *J* = 9.6 and 3.6 Hz, H-7B), 2.53 (1H, dd, *J* = 10.0 and 10.0 Hz, H-1), 3.71 (1H, m, H-2), 3.75 (2H, m, H-1′’), 3.86 (1H, q, *J* = 6.8 Hz, H-1′), 4.58 (1H, quintet, *J* = 7.5 Hz, C*H*(CH_2_CH_3_)_2_), 4.71 (1H, quintet, *J* = 7.5 Hz, C*H*(CH_2_CH_3_)_2_), 7.1-7.5 (10, m, Ar-H). ^13^C-NMR *δ* (ppm) (CDCl_3_): 9.4 (CH_3_, CH(CH_2_CH_3_)_2_), 9.5 (CH_3_, CH(CH_2_CH_3_)_2_x3), 15.5 (CH3, C-2′), 25.8 (CH_2_, C-3), 26.4 (CH_2_, CH(CH_2_CH_3_)_2_x3), 26.7 (CH_2_, CH(CH2CH_3_)_2_), 31.0 (CH_2_, C-4), 39.0 (CH_2_, C-7), 39.1 (CH, C-5), 50.0 (CH_2_, C-1′’), 55.3 (CH, C-1), 58.0 (CH, C-1′), 63.7 (CH, C-2), 76.6 (CH, CH(CH_2_CH_3_)_2_x2), 126.5 - 128.5 (CHx10, Ar), 141.6 (C, C*_ipso_*), 144.3 (C, C*_ipso_*), 171.7 (COOR, C-8), 174.3 (COOR, C-6).

When using diphenylketone as electrophile, 483 mg of crude product were obtained and chromatographed. The desired compounds were eluted with hexane/EtOAc 95:5 to afford 79 mg (yield 52%) of **17** and 99 mg (yield 15 %) of **16**.

Pentan-3-yl (1R,2R,5S)-2-(benzyl((R)-1-phenylethyl)amino)-5-((R)-1-hydroxy-3-oxo-3-(pentan-3-yloxy)-1,1-diphenylpropan-2-yl)cyclopentane-1-carboxylate (**17**): IR (cm^−1^): 3472, 2969, 1728, 1192, 1103, 746, 702. H.R.M.S.: calcd for C_46_H_57_NO_5_: 703.4237; found: 704.4325. ^1^H-NMR δ (ppm) (400 MHz, CHCl_3_): 0.66 (3H, t, *J* = 7.5 Hz, CH(CH_2_CH_3_)_2_), 0.72 (3H, t, *J* = 7.5 Hz, CH(CH_2_CH_3_)_2_), 0.85 (4H, m, CH(CH_2_CH_3_)_2_), 0.98 (3H, t, *J* = 7.5 Hz, CH(CH_2_CH_3_)_2_), 1.06 (3H, t, *J* = 7.5 Hz, CH(CH_2_CH_3_)_2_), 1.23 (3H, d, *J* = 6.8 Hz, H-2′), 1.40 (1H, m, H-4A), 1.53 (4H, m, CH(CH_2_CH_3_)_2_), 1.65 (2H, m, H-3), 1.75 (1H, m, H-4B), 2.15 (1H, m, H-5), 2.74 (1H, t, *J* = 10.1 Hz, H-1), 3.40 (1H, dd, *J* = 11.2 and 5.5 Hz, H-7), 3.75 (1H, m, H-2), 3.67 (1H, d, *J* = 12.4 Hz, H-1′’A), 3.71 (1H, d, *J* = 12.4 Hz, H-1′’B), 3.76 (1H, m, H-1′), 4.58 (1H, quintet, *J* = 5.7 Hz, CH(CH_2_CH_3_)_2_), 4.73 (1H, quintet, *J* = 5.7 Hz, CH(CH_2_CH_3_)_2_), 7.1 - 7.6 (20H, Ar-H). ^13^C-NMR δ (ppm) (CDCl_3_): 9.3 (CH_3_, CH(CH_2_CH_3_)_2_), 9.5 (CH_3_, CH(CH_2_CH_3_)_2_), 9.8 (CH_3_, CH(CH_2_CH_3_)_2_), 9.9 (CH_3_, CH(CH_2_CH_3_)_2_), 14.3 (CH_3_, C-2′), 25.4 (CH_2_, CH(CH_2_CH_3_)_2_), 25.8 (CH_2_, CH(CH_2_CH_3_)_2_), 25.9 (CH_2_, C-3), 26.1 (CH_2_, CH(CH_2_CH_3_)_2_x2), 26.2 (CH_2_, C-3), 28.5 (CH_2_, C- 4), 41.2 (CH, C-5), 50.0 (CH, C-1′’), 52.6 (CH_2_, C-1), 54.7 (CH, C-7), 57.2 (CH, C-1′), 61.8 (CH_2_, C-2), 77.9 (CH, CH(CH_2_CH_3_)_2_), 78.6 (CH, CH(CH_2_CH_3_)_2_), 79.3 (CH, C-1′’’), 125.2 –128.8 (CHx20, Ar), 141.3 (C, C_ipso_), 144.0 (C, C_ipso_), 144.2 (C, C_ipso_),148.2 (C, C_ipso_), 174.4 (COOR, C–8), 174.7 (COOR, C–6).

When using 1,2-epoxycyclohexane, 160 mg of crude product were obtained and chromatographed to obtain 86 mg (yield 43%) of **18** and 17 mg (yield 10%) of **16**.

Pentan-3-yl (1R,2R,5R)-2-(benzyl((R)-1-phenylethyl)amino)-5-((R)-1-((1S,2R)-2-hydroxycyclohexyl)-2-oxo-2-(pentan-3-yloxy)ethyl)cyclopentane-1-carboxylate (**18**): IR (cm-^1^): 3511, 2969, 1726, 1458, 1117, 748, 700. ^1^H-NMR δ (ppm) (200 MHz, CDCl_3_): 0.78-0.96 (9H, m, CH(CH_2_CH_3_)_2_), 1.11 (3H, m, CH(CH_2_CH_3_)_2_), 1.29 (3H, d, *J* = 6.8 Hz, H-2′), 1.42-1.93 (8H, m, CH(CH_2_CH_3_)_2_; 2H, m, H-3; 2H, m, H-4; 1H, H-1′’’), 2.43 (1H, dd, H-7), 2.94 (1H, m, H-5), 3.04 (1H, t, *J* = 7.1 Hz, H-1), 3.61 (1H, q, *J* = 7.7 Hz, H-2), 3.67 (1H, d, *J* = 14.3, H-1′’A), 3.87 (1H, d, *J* = 14.3, H-1′’B), 3.89 (1H, q, *J* = 6.8 Hz, H-1′), 4.17 (1H, -OH), 4.75 (2H, m, CH(CH_2_CH_3_)_2_x2), 7.1-7.6 (10H, Ar-H).

When using ClCOOEt as electrophile, 275 mg of crude product were obtained and chromatographed. The desired product was eluted in hexane/EtOAc 95:5 to furnish 80 mg (yield 54%) of **19**.

*1-Ethyl-3-(pentan-3-yl) 2-((1S,2R,3R)-3-(benzyl((R)-1-phenylethyl)amino)-2-((pentan-3-yloxy)carbonyl)- cyclopentyl)malonate* (**19**): IR (cm^−1^): 2972, 1732, 1462, 1113, 1030, 910, 748. H.R.M.S.: calcd for C_36_H_51_NO_6_: 593.3716; found: 593.3793. ^1^H-NMR *δ* (ppm) (400 MHz, CDCl_3_): 0.82 (3H, m, CH(CH_2_C*H*_3_)_2_), 0.84 (3H, t, *J* = 7.5 Hz, CH(CH_2_C*H*_3_)_2_x2), 0.86 (3H, m, CH(CH_2_C*H*_3_)_2_), 1.21 (3H, t, COOCH_2_C*H*_3_) 1.31 (3H, d, *J* = 6.8 Hz, H-2′), 1.35-1.47 (4H, m, CH(C*H*_2_CH_3_)_2_), 1.52-1.58 (4H, m, CH(C*H*_2_CH_3_)_2_) 1.55 (1H, m, H-4A), 1.75 (2H, m, H-3),1.87 (1H, m, H-4B), 2.68 (1H, m, H-5), 2.62 (1H, d, *J* = 7.5, H-7), 2.76 (1H, t, *J* = 7 Hz, H-1), 3.32 (1H, d, *J* = 6Hz, H-7), 3.40 (1H, d, *J* = 6Hz, H-7), 3.68 (1H, d, *J* = 4.2 Hz, H-1′’A), 3.71 (1H, d, *J* = 4.2 Hz, H-1′’B), 3.89 (1H, q, *J* = 6.8 Hz, H-1′), 4.12 (2H, m, COOC*H*_2_CH_3_), 4.64 (1H, quintet, *J* = 6.0, C*H*(CH_2_CH_3_)_2_), 4.76 (1H, quintet, *J* = 6 Hz, C*H*(CH_2_CH_3_)_2_), 7.1-7.6 (10H, Ar-H). ^13^C-NMR *δ* (ppm) (CDCl_3_): 9.7 (CH_3_, CH(CH_2_CH_3_)_2_ × 2), 9.8 (CH_3_, CH(CH_2_CH_3_)_2_x2), 15.4 (CH_3_, C-2′), 14.3 (CH_3_, COOCH_2_CH_3_), 15.6 (CH_3_, C-2′), 25.6 (CH_2_, CH(CH_2_CH_3_)_2_x2), 26.4 (CH_2_, CH(CH_2_CH_3_)_2_x2), 27.8 (CH_2_, C-3), 27.9 (CH_2_, C-4), 27.5 (CH_2_, C-4), 41.1 (CH, C-5), 50.2 (CH_2_, C-1′’), 50.3 (CH_2_, C-1′’), 52.8 (CH, C-1), 53.0 (CH, C-1), 54.3 (CH_2_, C-7), 55.0 (CH_2_, C-7), 57.7 (CH, C-1′), 61.3 (CH_2_, COOCH_2_CH_3_), 64.5 (CH, C-2), 64.8 (CH, C-2), 76.6 (CH, CH(CH_2_CH_3_)_2_), 76.8 (CH, CH(CH_2_CH_3_)_2_), 77.9 (CH, CH(CH_2_CH_3_)_2_), 78.3 (CH, CH(CH_2_CH_3_)_2_), 124.4 (CH2, C-1′’’), 126.1 - 128.8 (CHx10, Ar), 141.8 (C, C*_ipso_*), 141.8 (C, C*_ipso_*), 144.4 (C, C-7), 168.7 (COOR, C-8), 174.6 (COOR, C-6).

### 4.4. Synthesis of Nepetalactone Derivative ***26***

We started the synthesis similarly to the preparation of **14**, so 49 mg of this compound were obtained. Then, to a solution of **14** in benzene (3.5 mL), NaOMe (4.8 mg, 0.089 mmol) was added and the reaction mixture heated up to reflux for 4 h. Then, the mixture was extracted with ether and washed with H_2_O and brine. After being dried with Na_2_SO_4_ and filtered, the solvent was evaporated and 48 mg (yield 100%) of **20** were thus obtained. A quantitative amount of **20** was also obtained when treating **14** (47 mg) with NaH (2.5 mg, 0.103 mmol) under an Ar atmosphere for 6 h. Then, the reaction mixture was washed with H_2_O and dissolved in ether. After being dried with Na_2_SO_4_, filtered and the solvent evaporated, 46 mg of **20** (100% yield) were obtained.

Pentan-3-yl (1R,2R,5S)-2-(benzyl((R)-1-phenylethyl)amino)-5-(3-oxo-3-(pentan-3-yloxy)prop-1-en-2-yl)-cyclopentane-1-carboxylate (**20**): IR (cm^−1^): 2969, 2940, 2880, 1724, 1495, 1464, 1373, 1271, 1161, 1117, 1028, 943, 746, 698. ^1^H-NMR δ (ppm) (400 MHz, CDCl_3_): 0.71 (3H, t, *J* = Hz, CH(CH_2_CH_3_)_2_), 0.83 (6H, m, CH(CH_2_CH_3_)_2_), 0.90 (3H, t, *J* = Hz, CH(CH_2_CH_3_)_2_), 1.30 (3H, d, *J* = Hz, NCHCH_3_), 1.54 (4H, m, H-3, H-4), 2.94 (2H, m, CH(CH_2_CH_3_)_2_), 3.03 (2H, m, CH(CH_2_CH_3_)_2_), 3.63 (2H, m, NCH_2_Ph), 3.71 (1H, quintet, *J* = Hz NCHCH_3_), 4.57 (1H, m, H-5), 4.79 (1H, m, H-1), 5.53 (1H, s, CCH_2_-A), 6.08 (1H, s, CCH_2_-B), 7.16-7.31 (10H, Ar). RMN ^13^C δ (ppm) (CDCl_3_): 9.4 (CH_3_, CH(CH_2_CH_3_)_2_x4, 15.0 (CH_3_, NCHCH_3_), 25.6 (CH_2_, CH(CH_2_CH_3_)_2_x2), 26.6 (CH_2_, CH(CH_2_CH_3_)_2_ × 2), 31.8 (CH_2_, C-3, C-4), 45.4 (CH, C-5), 50.2 (CH_2_, NCH_2_Ph), 53.5 (CH, C-1), 57.7 (CH, NCH), 63.6 (CH, NCHCH_3_), 77.9 (CH, CH(CH_2_CH_3_)_2_), 124.4 (CH_2_, CCH_2_), 141.7 (C_ipso_x2), 144.4 (C, C-7), 166.6 (C, COOR), 174.5 (C, COOR).

To a solution of **14** (38 mg, 0.07 mmol) in benzene (3 mL), HCl gas was added at 0 °C and then, a catalytic amount of *p*TsOH was added and the reaction mixture was heated up to 60 °C for 24 h. Then, the resulting mixture was extracted with ether and washed with NaHCO_3_ saturated solution and H_2_O. After being dried with Na_2_SO_4_, filtered and the solvent evaporated, the desired compound **21** was eluted with hexane/EtOAc 95:5. 7 mg (yield 30%) were obtained.

*Pentan-3-yl (4S,4aS,7R)-7-(benzyl((R)-1-phenylethyl)amino)-1-oxooctahydrocyclopenta[c]pyran-4-carboxylate* (**21**): H.R.M.S.: calcd for C_29_H_37_NO_4_: 479.3036; found: 363.2764. ^1^H-NMR *δ* (ppm) (400 MHz, CDCl_3_): 0.83 (3H, t, *J* = 5.0 Hz, CH(CH_2_C*H*_3_)_2_), 0.86 (3H, t, *J* = 5.0 Hz, CH(CH_2_C*H*_3_)_2_), 1.34 (3H, d, *J* = 6.9 Hz, H-2′), 1.51 (1H, m, H-6A), 1.53 (1H, m, H-7A), 1.55-1.79 (4H, m, CH(C*H*_2_CH_3_)_2_), 1.79 (1H, m, H-6B), 1.82 (1H, m, H-7B), 2.74 (1H, dd, *J* = 11.6 and 4.6 Hz, H-9), 2.94 (1H, m, H-5), 3.07 (1H, ddd, *J* = 9.8, 9.8 and 7.5 Hz, H-4), 3.73 (1H, m, H-8), 3.76 (1H, d, *J* = 15.1 Hz, H-1′’A), 3.85 (1H, m, H-1′), 3.89 (1H, d, *J* = 15.1 Hz, H-1′’B), 4.11 (1H, dd, **J* =* 17.9 and 7.5 Hz, H-3B), 4.30 (1H, dd, *J* = 11.7 and 9.8 Hz, H-3A), 4.74 (1H, quintet, *J* = 6 Hz, C*H*(CH_2_CH_3_)_2_) 7.1-7.6 (10H, Ar-H). ^13^C-NMR δ (ppm) (CDCl_3_): 9.5 (CH_3_, CH(CH_2_CH_3_)_2_x2), 17.3 (CH_3_, C-2′), 25.9 (CH_2_, CH(CH_2_CH_3_)_2_x2), 27.3 (CH_2_, C-7), 27.8 (CH_2_, C-6), 40.0 (CH, C-5), 115 40.8 (CH, C-4), 46.3 (CH, C-8), 49.8 (CH_2_, C-1′’), 58.2 (CH, C-1′), 59.8 (CH, C-9), 67.3 (CH_2_, C-3), 77.9 (CH, CH(CH_2_CH_3_)_2_), 126.4-128.1 (10C, Ar), 142.8 (C, C*_ipso_*), 144.8 (C, C*_ipso_*), 170.8 (C, C-1), 173.9 (COOR, C-11).

Also, to a solution of **14** (85 mg) in CH_2_Cl_2_ (4 mL), *m*CPBA (66 mg, 0.385 mmol) was added. After 6 h of stirring, saturated Na_2_S_2_O_3_ solution (4 mL) was added and the resulting mixture was eluted with CH_2_Cl_2_ and washed with H_2_O, NaHCO_3_ and saturated Na_2_S_2_O_3_ solution. The resulting mixture was dried with Na_2_SO_4_ and, after being filtered, it was chromatographed. The desired compound was eluted with hexane/EtOAc 95:5 to give 31 mg (yield 82%) of **22**. Also, when the reaction was performed in one pot, starting from compound **2** and adding (*R*)-**1**, HCHO and then, directly at the reaction mixture, *m*CPBA was added, both **22** (yield 69%) and **23** (yield 6%) were obtained.

*Pentan-3-yl (R)-5-((S)-3-hydroxy-1-oxo-1-(pentan-3-yloxy)propan-2-yl)cyclopent-1-ene-1-carboxylate* (**22**): [α]_D_^26^ = + 9.6 (CHCl_3_, c = 1.00). IR (cm^−1^): 3524, 2880, 1709, 1098, 920. H.R.M.S.: calcd for C_19_H_32_O_5_: 340.2250; found: 340.2218. ^1^H-NMR *δ* (ppm) (400 MHz, CDCl_3_): 0.90 (12H, m, CH(CH_2_CH_3_)_2_), 1.59 (8H, m, CH(CH_2_CH_3_)_2_), 1.88 (1H, m, H-4B), 2.08 (1H, m, H-4A), 2.44 (1H, m, H-3B), 2.49 (1H, m, H-3A), 3.29 (1H, q, *J* = 4.4 Hz, H-7), 3.57 (1H, dd, *J* = 11.6 and 4.1 Hz, H-1′’’A), 3.62 (1H, m, H-5), 3.80 (1H, dd, *J* = 11.6 and 8.1 Hz, H-1′’’B), 4.83 (2H, q, *J* = 6.0 Hz, CH(CH_2_CH_3_)_2_), 6.86 (1H, dd, *J* = 2.4 Hz, H-2). ^13^C-NMR *δ* (ppm) (CHCl_3_): 10.1 (CH_3_, CH(CH_2_CH_3_)_2_x4), 26.8 (CH_2_, CH(CH_2_CH_3_)_2_x2), 26.9 (CH_2_, CH(CH_2_CH_3_)_2_x2), 27.0 (CH_2_, C-4), 32.8 (CH_2_, C-3), 44.5 (CH, C-5), 49.4 (CH, C-7), 60.3 (CH2, C-1′’’), 76.9 (CH, CH(CH_2_CH_3_)_2_), 77.6 (CH, CH(CH_2_CH_3_)_2_), 137.2 (C, C-1), 146.1 (C, C-2), 165.3 (C, C-6), 175.1 (C, C-8).

*Pentan-3-yl (S)-5-((S)-3-hydroxy-1-oxo-1-(pentan-3-yloxy)propan-2-yl)cyclopent-1-ene-1-carboxylate* (**23**): IR (cm^−1^): 3493, 2969, 2880, 1711, 1632, 1462, 1383, 1267, 1202, 1101, 1047, 934, 752. ^1^H-NMR *δ* (ppm) (400 MHz, CDCl_3_): 0.90 (12H, m, CH(CH_2_C*H*_3_)_2_), 1.51 (8H, m, CH(C*H*_2_CH_3_)_2_), 1.85 (1H, m, H-4A), 2.15 (1H, m, H-4B), 2.44 (1H, m, H-3A), 2.62 (1H, m, H-3B), 3.05 (1H, q, *J* = 4.4 Hz, H-7), 3.33 (1H, dd, *J* = 11.6 and 4.1 Hz, H-1′’’A), 3.75 (1H, m, H-5), 3.79 (1H, dd, *J* = 11.6 and 8.1 Hz, H-1′’’B), 4.76 (2H, quintet, *J* = 6.0 Hz, C*H*(CH_2_CH_3_)_2_ × 2), 6.80 (1H, m, H-2). ^13^C-NMR *δ* (ppm) (CHCl_3_): 9.82 (CH_3_, CH(CH_2_CH_3_)_2_x4), 26.53 (CH_2_, CH(CH_2_CH_3_)_2_x4), 27.9 (CH_2_, C-4), 31.8 (CH_2_, C-3), 43.3 (CH, C-5), 50.3 (CH, C-7), 63.1 (CH2, C-1′’’), 77.0 (CH, CH(CH_2_CH_3_)_2_), 77.5 (CH, CH(CH_2_CH3)_2_), 138.2 (C, C-1), 145.2 (C, C-2), 165.6 (C, C-6), 173.9 (C, C-8).

To a solution of **23** (8.5 mg, 0.025 mmol) in benzene (3 mL), a catalytic amount of *p*TsOH was added and the reaction mixture was heated up to 60 °C for 24 h, then, it was extracted with ether and washed with saturated NaHCO_3_ solution and H_2_O. The resulting mixture was dried with Na_2_SO_4_, filtered and the solvent evaporated. After chromatography, 5 mg (yield 65%) of the desired compound **24** were eluted with hexane/EtOAc 9:1.

*Pentan-3-yl (4S,4aR)-1-oxo-1,3,4,4a,5,6-hexahydrocyclopenta[c]pyran-4-carboxylate* (**24**): IR (cm^−1^): 3445, 2969, 1728, 1636, 1464, 1402, 1256, 1157, 1092, 1057, 910, 733. ^1^H-NMR *δ* (ppm) (400 MHz, CDCl_3_): 0.87 (6H, m, CH(CH_2_C*H*_3_)_2_x2), 1.55 – 1.62 (4H, m, CH(C*H*_2_CH_3_)_2_), 1.78 (1H, m, H-6B), 2.31 (1H, m, H-6A), 2.48 (2H, m, H-7), 2.69 (1H, ddd, *J* = 11.5, 11.5 and 3.1 Hz, H-4), 3.19 (1H, m, H-5), 4.32 (1H, dd, *J* = 11.9 and 3.5 Hz, H-3A), 4.52 (1H, dd, *J* = 11.9 and 2.9 Hz, H-3B), 4.80 (1H, quintet, *J* = 6 Hz C*H*(CH_2_CH_3_)_2_), 7.07 (1H, m, H-8). RMN ^13^C *δ* (ppm) (CDCl_3_): 9.5 (CH_3_, CH(CH_2_CH_3_)_2_x2), 26.3 (CH_2_, CH(CH_2_CH_3_)_2_x2), 31.7 (CH_2_, C-6), 31.9 (CH_2_, C-7), 45.0 (CH, C-4), 47.0 (CH, C-5), 69.6 (CH_2_, C-3), 77.9 (CH, CH(CH_2_CH_3_)_2_), 132.4 (C, C-9), 147.4 (C, C-8), 162.3 (C, C-1), 170.2 (COOR, C-11).

To a solution of **22** (13 mg, 0.038 mmol) in benzene (3 mL), a catalytic amount of *p*TsOH was added and the reaction flask was heated to 60 °C for 24 h. Then, the reaction mixture was diluted with ether and washed with saturated NaHCO_3_ solution and H_2_O. The resulting mixture was dried with Na_2_SO_4_ and, after being filtered and the solvent evaporated, it was chromatographed and the desired compound was eluted with hexane/EtOAc 9:1 to give 6 mg (yield 77%) of **25**.

*Pentan-3-yl (4S,4aR)-1-oxo-1,3,4,4a,5,6-hexahydrocyclopenta[c]pyran-4-carboxylate* (**25**): [α]_D_^26^ = -0.56 (CHCl_3_, c = 0.89). IR (cm^−1^): 2969, 1728, 1256, 1157, 910, 733. H.R.M.S.: calcd for C_14_H_20_O_4_: 252.3062; found: 252.2386. RMN ^1^H *δ* (ppm) (400 MHz, CDCl_3_): 0.84 (3H, t, *J* = 7.4 Hz, CH(CH_2_C*H*_3_)_2_), 0.87 (3H, t, *J* = 7.4 Hz, CH(CH_2_C*H*_3_)_2_), 1.53 – 1.60 (4H, m, CH(C*H*_2_CH_3_)_2_), 1.91 (1H, m, H-6A), 2.31 (1H, m, H-6B), 2.51 (1H, m, H-7A), 2.51 (1H, m, H-7B), 3.02 (1H, ddd, *J* = 11.5, 11.5 and 3.1 Hz, H-4), 3.31 (1H, m, H-5), 4.35 (1H, dd, *J* = 11.9 and 3.5 Hz, H-3A), 4.55 (1H, dd, *J* = 11.9 and 2.9 Hz, H-3B), 4.80 (1H, quintet, *J* = 6 Hz C*H*(CH_2_CH_3_)_2_), 6.98 (1H, m, H-8). RMN ^13^C *δ* (ppm) (CDCl_3_): 9.2 (CH_3_, CH(CH_2_CH_3_)_2_), 9.8 (CH_3_, CH(CH_2_CH_3_)_2_), 26.4 (CH_2_, CH(CH_2_CH_3_)_2 ×_ 2), 29.3 (CH_2_, C-6), 31.9 (CH_2_, C-7), 43.1 (CH, C-4), 43.8 (CH, C-5), 69.8 (CH_2_, C-3), 78.2 (CH, CH(CH_2_CH_3_)_2_), 132.7 (C, C-9), 145.3 (C, C-8), 163.2 (C, C-1), 170 (COOR, C-11).

Finally, when it comes to the syntheses of nepetalactone derivative **26**, first, a lithium dimethyl-cuprate solution was prepared by adding MeLi 1.6 M (0.13 mL, 0.21 mmol) to a solution of CuI (20 mg, 0.103 mmol) in ether (2.50 mL) at 0 °C. Then, **25** (13 mg, 0.05 mmol) in ether (1.50 mL) was added and, after 90 min of stirring, 3.00 mL of NH_4_Cl saturated solution were added. The resulting reaction mixture was diluted with ether and dried with Na_2_SO_4_. After being filtered, 11 mg of crude product were obtained and chromatographed. The desired nepetalactone was eluted in hexane/EtOAc 95:5 To afford 10 mg (yield 80%) of **26**.

*Pentan-3-yl (4S,4aS,7S,7aR)-7-methyl-1-oxooctahydrocyclopenta[c]pyran-4-carboxylate* (**26)**: IR (cm^−1^): 2967, 1736, 1196, 1034, 909. H.R.M.S.: calcd for C_15_H_24_O_4_: 268.1675; found: 269.1768. ^1^H-NMR *δ* (ppm) (400 MHz, CDCl_3_): 0.88 (3H, t, *J* = 5.0 Hz, CH(CH_2_C*H*_3_)_2_), 0.89 (3H, t, *J* = 5.0 Hz, CH(CH_2_C*H*_3_)_2_), 1.20 (3H, d, *J* = 6.4 Hz, H-10), 1.50 (1H, m, H-6A), 1.54–1.60 (4H, m, CH(C*H*_2_CH_3_)_2_), 1.88 (1H, m, H-6B), 1.98 (1H, m, H-7A), 2.07 (1H, m, H-8), 2.26 (1H, m, H-7B), 2.51 (1H, dd, *J* = 10.5 and 10.3 Hz, H-9), 2.94 (1H, m, H-5), 3.06 (1H, ddd, *J* = 10.4, 6.1 and 4.6 Hz, H-4), 4.37 (1H, m, H-3A), 4.41 (1H, m, 1.5 Hz, H-3B), 4.78 (1H, quintet, *J* = 6 Hz, C*H*(CH_2_CH_3_)_2_). ^13^C-NMR *δ* (ppm) (CDCl_3_): 9.8 (CH_3_, CH(CH_2_CH_3_)_2 ×_ 2), 19.0 (CH_3_, C-10), 26.4 (CH_2_, CH(CH_2_CH_3_)_2 ×_ 2), 28.1 (CH_2_, C-7), 35.3 (CH_2_, C-6), 37.4 (CH, C-8), 42.1 (CH, C-5), 42.7 (CH, C-4), 50.5 (CH, C-9), 64.7 (CH2, C-3), 78.0 (CH, CH(CH_2_CH_3_)_2_), 170.4 (C, C-1), 173.2 (COOR, C-11).

## Figures and Tables

**Table 1:** One bond and long-range 2D ^1^H-^13^C correlation for compounds **4**, **5** and **6**.

	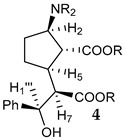			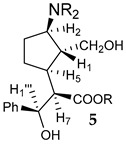			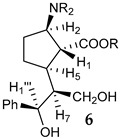		
CARBON	*δ*C/ppm	Observed ^1^H (*δ)*. One Bond Correlation	Observed Long Range Correlation	*δ*C/ppm	Observed ^1^H (*δ)*. One Bond Correlation	Observed Long Range Correlation	*δ*C/ppm	Observed ^1^H (*δ)*. One Bond Correlation	Observed Long Range Correlation
**1**	50.0	3.23, dd, 10.1, 9.3	2, 3, 4, 5, 7	45.1	2.24, m	2	53.0	2.73, t, 9.1	5, 3
**2**	65.1	3.56, ddd, 9.9, 8.2, 8.2	1, 1′, 1′’, 3, 4	60.7	2.84, m	1, 1′, 3	65.1	3.55, m	1, 1′, 3
**3**	26.6	1.75, m	4	25.7	1.64, m	4	27.1	1.65, m 1.75, m	4
**4**	28.8	1.55, m 1.85, m	2	28.5	1.75, m	5	30.3	1.26, m 1.80, m	5
**5**	40.0	2.74, m	1, 3, 4, 7	36.1	2.84, m	1′’’	39.8	2.27, m	1
**6**	177.4		1, 2	64.7	3.55, m 3.44, m	2	176.6		1, 2
**7**	56.5	2.87, dd, 10.0, 3.8	1, 5, OH	57.2	2.83, m	1′’’	50.6	2.29, m	1
**8**	172.0		5, 7	173.0		7	63.4	3.51, d, 4.5	5, 7
**1′**	57.2	3.90, q, 6.8	1′’, 2′, H_Ar_	55.4	3.94, q, 6.8	1′’, 2′	58.0	3.90, q, 6.8	1′’, 2′
**2′**	15.1	1.28, d, 6.8	1′	12.4	1.38, d, 6.8	1′	15.1	1.34, d, 6.8	1′
**1′’**	49.9	3.85, d, 14.4 3.67, d, 14.4	H_Ar_, 1′	50.1	3.91, d, 14.4 3.58, d, 14.4	H_Ar_, 1′, 2	50.0	3.69, d, 14.6 3.77, d, 14.6	H_Ar_, 1′, 2
**1′’’**	73.3	4.64, dd, 10.0, 3.5	H_Ar_, 7	73.3	4.96, d, 6.5	7	75.6	4.86, d, 6.5	8
**-CH(CH2CH3)2**	**77.4** **77.1**	**4.67, q, 6.0** **4.44, q, 6.0**	**CH_2_, CH_3_**	**76.8**	**4.58, q, 6.0**	**CH_2_, CH_3_**	**77.0**	**4.58, q, 5.9**	**CH_2_, CH_3_**
**-CH(CH2CH3)2**	25.4 25.2 25.0 24.5	1.08, m 1.35, m 1.64, m 1.75, m	CH_3_	25.3 25.4	1.38, m 1.13, m	CH_3_	24.8 25.1	1.35, m 1.41, m	CH_3_
**-CH(CH2CH3)2**	9.4 9.2 9.0 8.6	1.00, t, 7.5 0.98, t, 7.5 0.73, t, 7.5 0.32, t, 7.5	CH_2_	8.6 9.7	0.37, t, 7.5 0.78, t, 7.5	CH_2_	9.0 9.2	0.83, t, 7.6 0.89, t, 7.6	CH_2_
**OH**		3.04, d, 3.5							

**Table 2:**
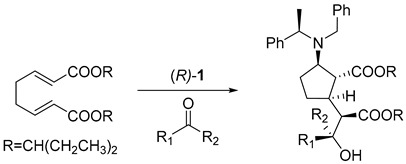
Electrophile additions.

Entry	Electrophile	Products	Yield
1	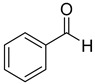 Benzaldehyde; R_1_=Ph, R_2_=H	**3** + **4**	**3**: 23%; **4**: 45%
2	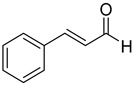 Cinnamaldehyde; R_1_=PhCH=CH, R_2_=H	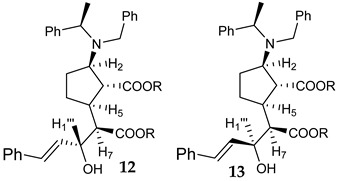	**12**: 30%; **13**: 27%
3	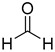 Formaldehyde; R_1_=R_2_=H	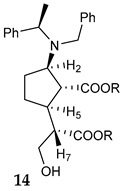	**14**: 80%
4	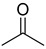 Acetone; R_1_=R_2_=Me	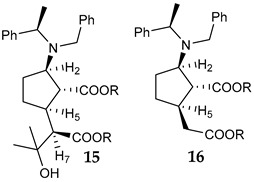	**15**: 27%; **16**: 25%
5	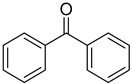 Diphenylketone; R_1_=R_2_=Ph	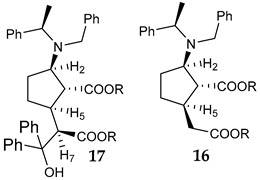	**17**: 52%; **16**: 15%
6	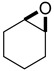 1,2-epoxycyclohexane	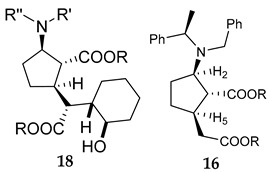	**18**: 43%; **16**: 10%
7	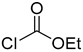 Ethyl Chloroformate; R_1_= Cl; R_2_= OEt	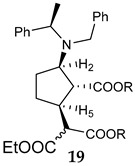	**19**: 54%

## Supplementary Materials

The following are available online. Characterization data, IR, ^1^H NMR, ^13^C NMR and HRMS Spectra of products and HMQC, HMBC, COSY and ROESY, where it has been carried out.
