# Supplementary material for: Multicomponent Domino Reaction in the Asymmetric Synthesis of Cyclopentan[c]pyran Core of Iridoid Natural Products
Source: Molecules. 2020 Mar 13;25(6):1308. doi: 10.3390/molecules25061308 (PMC7144114; doi:10.3390/molecules25061308)
Supplement: Supplementary file 1 [file molecules-25-01308-s001.pdf]

Supporting Information

Multicomponent Domino Reaction in the Asymmetric Synthesis of Cyclopentan[c]pyran Core of Iridoid Natural Products.

Alejandro Manchado, Victoria Elena Ramos, David Díez and Narciso M. Garrido.*

Dpto. de Química Orgánica, Facultad de Ciencias Químicas, Universidad de Salamanca, Plaza de los Caídos 1-5, 37008 Salamanca, Spain.

Table of Contents **Page No.**

IR, ^{1}H RMN, ^{13}C RMN and COSY for 3 …… 2
IR, ^{1}H RMN and ^{13}C RMN for 4 …… 4
IR, ^{1}H RMN, ^{13}C RMN, HMQC, HMBC and HRMS for 5 …… 6
IR, ^{1}H RMN, ^{13}C RMN, HMQC, HMBC, COSY and HRMS for 6 …… 9
IR, ^{1}H RMN, ^{13}C RMN and HRMS for 7 …… 13
IR, ^{1}H RMN and ^{13}C RMN for 8 …… 15
IR, ^{1}H RMN, ^{13}C RMN, ROESY, COSY and HRMS for 9 …… 17
^{1}H RMN, ^{13}C RMN and HRMS for 10 …… 20
IR, ^{1}H RMN and ^{13}C RMN for 11 …… 22
IR, ^{1}H RMN, ^{13}C RMN and HRMS for 12 …… 25
IR and ^{1}H RMN for 13 …… 26
IR, ^{1}H RMN, ^{13}C RMN and HRMS for 14 …… 27
^{1}H RMN and ^{13}C RMN for 15 …… 29
IR, ^{1}H RMN and ^{13}C RMN for 16 …… 30
IR, ^{1}H RMN, ^{13}C RMN and HRMS for 17 …… 32
IR and ^{1}H RMN for 18 …… 34
IR, ^{1}H RMN, ^{13}C RMN and HRMS for 19 …… 35
IR, ^{1}H RMN and ^{13}C RMN for 20 …… 37
^{1}H RMN, ^{13}C RMN, ROESY, COSY and HRMS for 21 …… 39
IR, ^{1}H RMN, ^{13}C RMN, COSY and HRMS for 22 …… 42
IR, ^{1}H RMN, ^{13}C RMN and HRMS for 23 …… 45
^{1}H RMN, ^{13}C RMN and COSY for 24 …… 47
IR, ^{1}H RMN, ^{13}C RMN, COSY and HRMS for 25 …… 49
IR, ^{1}H RMN, ^{13}C RMN, COSY and HRMS for 26 …… 52

* Corresponding author. Tel.: +34-666589065; fax: +34-923-294574; e-mail: nmg@usal.es

1. IR, ^{1}H RMN, ^{13}C RMN and COSY for 3

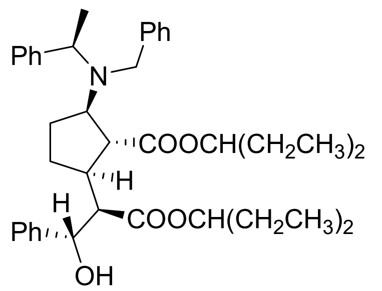

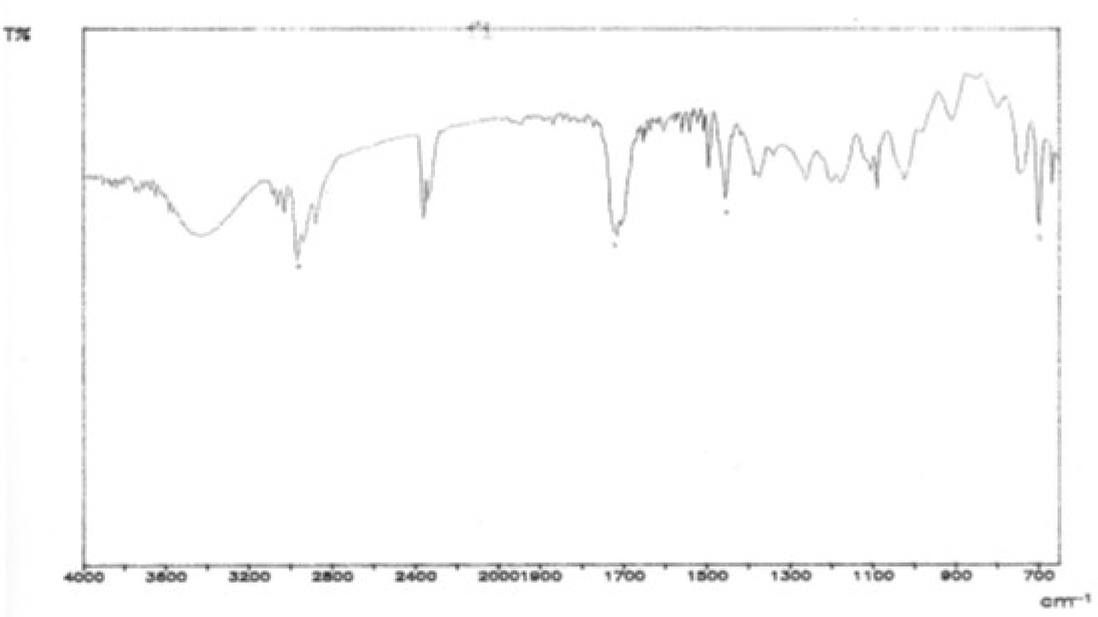

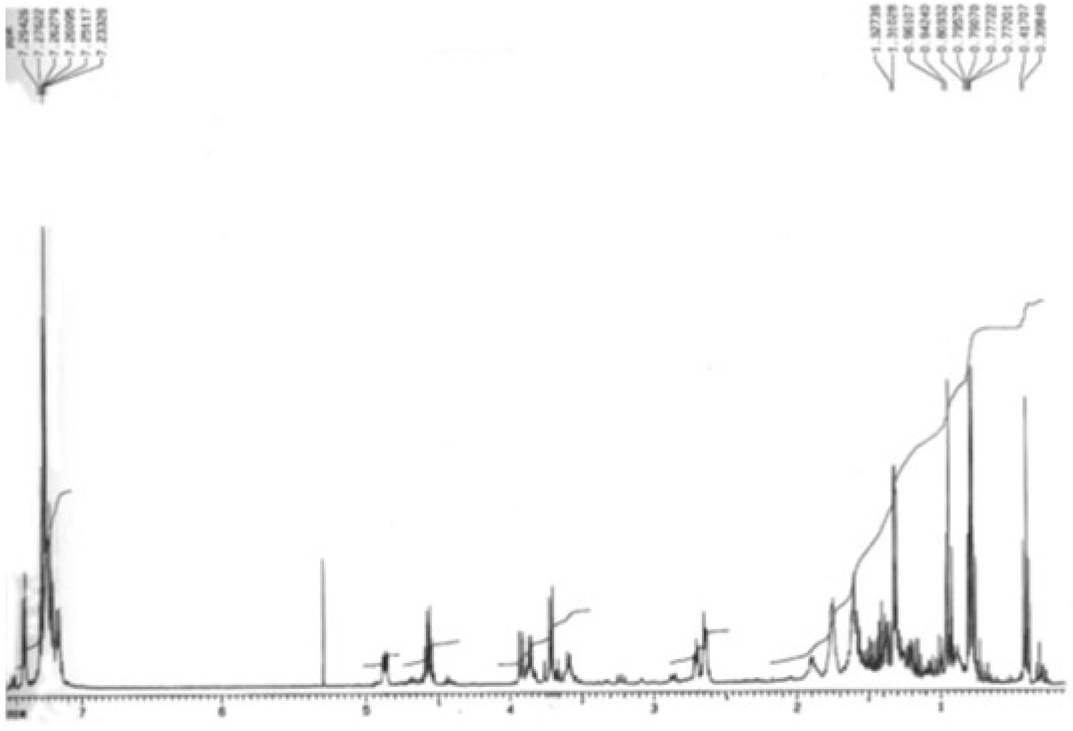

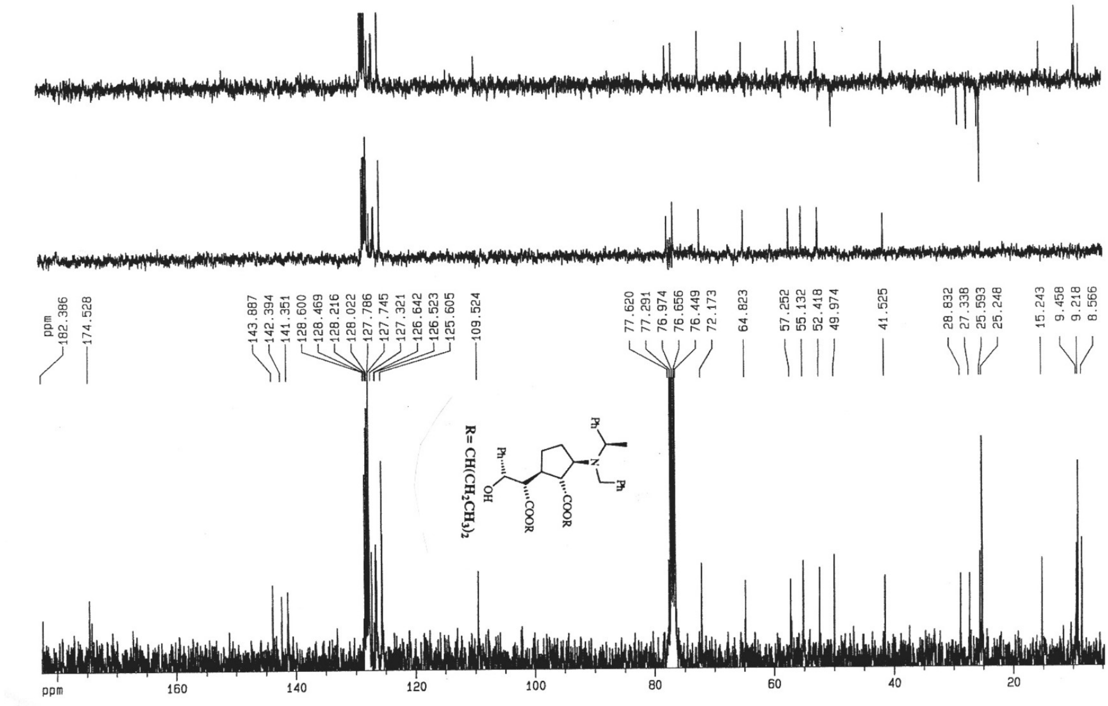
ppm
182.386
174.528
143.887
142.394
141.351
128.600
128.469
128.216
128.022
127.786
127.745
127.321
126.642
126.523
125.605
109.524
77.620
77.291
76.974
76.656
76.449
72.173
64.823
57.252
55.132
52.418
49.974
41.525
28.832
27.338
25.593
25.248
15.243
9.458
9.218
8.566
Ph
N
Ph
COOR
COOR
OH
Ph
R= CH(CH2CH3)2
ppm
160
140
120
100
80
60
40
20

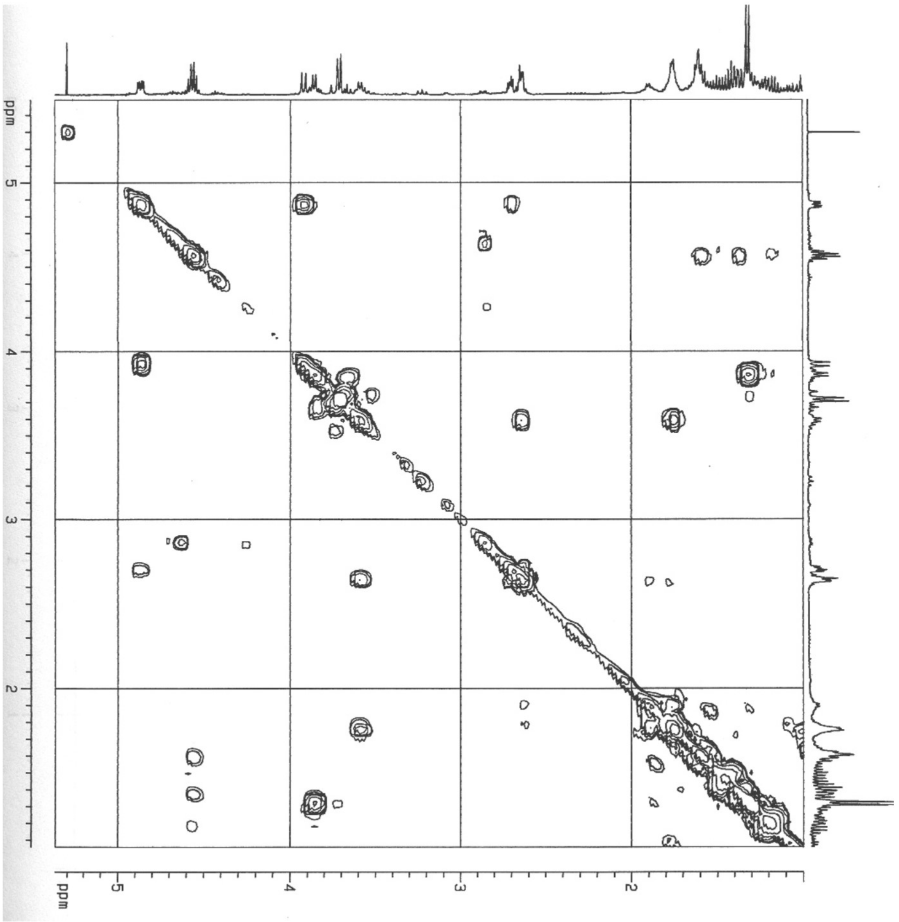
ppm
5
4
3
2
ppm

2. IR, ^{1}H RMN and ^{13}C RMN for 4

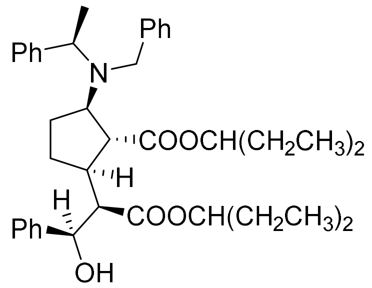

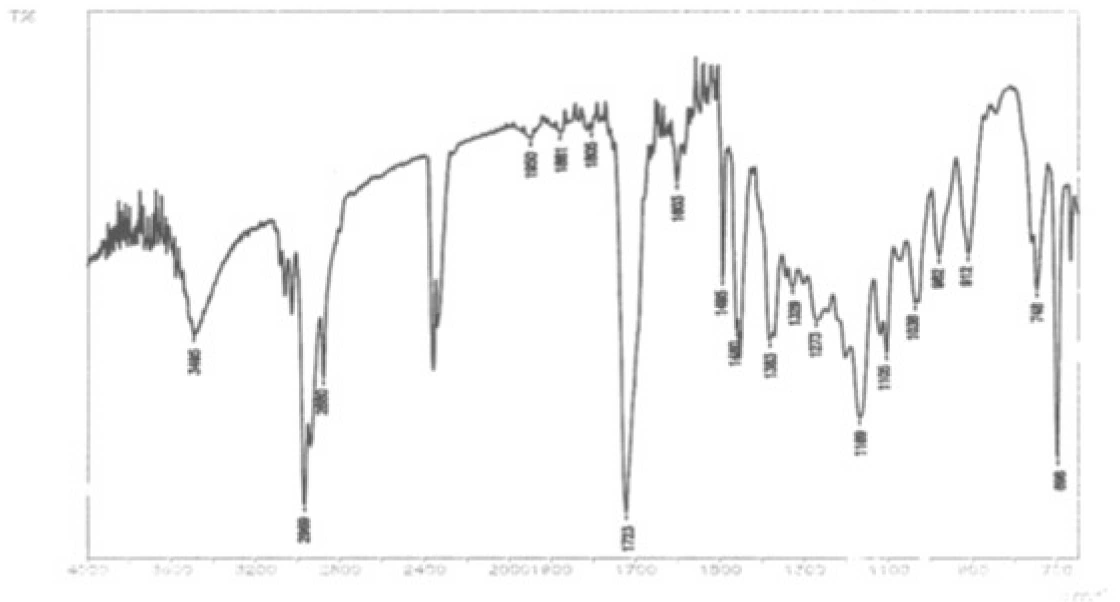

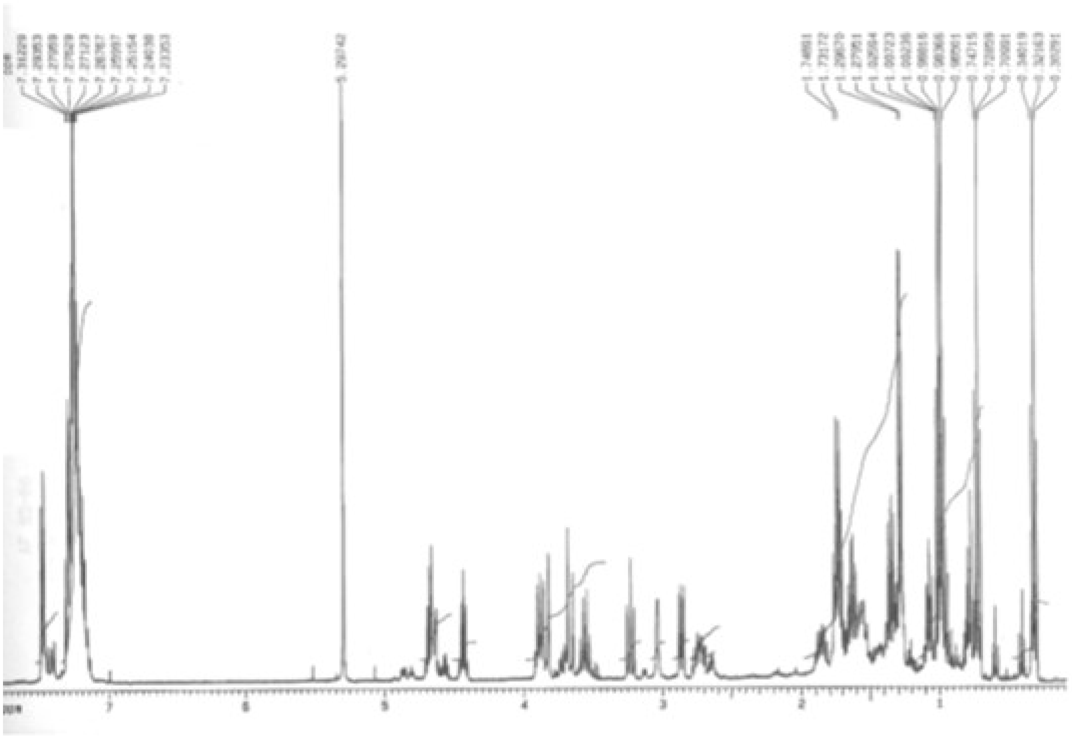

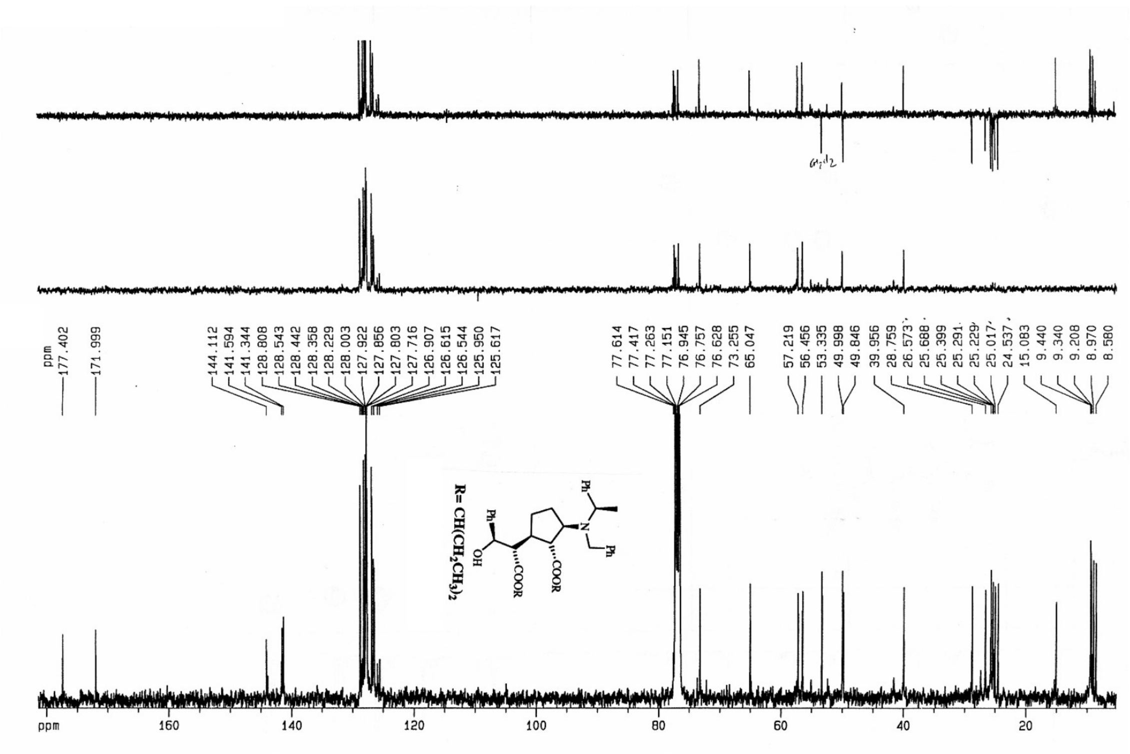
ppm
177.402
171.999
144.112
141.594
141.344
128.808
128.543
128.442
128.358
128.229
128.003
127.922
127.856
127.803
127.716
126.907
126.615
126.544
125.950
125.617
77.614
77.417
77.263
77.151
76.945
76.757
76.628
73.255
65.047
57.219
56.456
53.335
49.998
49.846
39.956
28.759
9.440
9.340
9.208
8.970
8.580
15.083
Ph
N
COOR
OH
R= CH(CH2CH3)2
ppm
160
140
120
100
80
60
40
20

3. IR, ^{1}H RMN, ^{13}C RMN, HMQC, HMBC and HRMS for 5

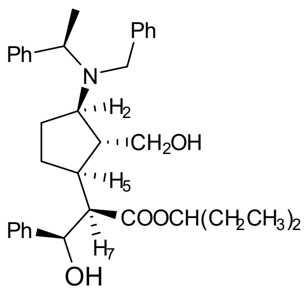

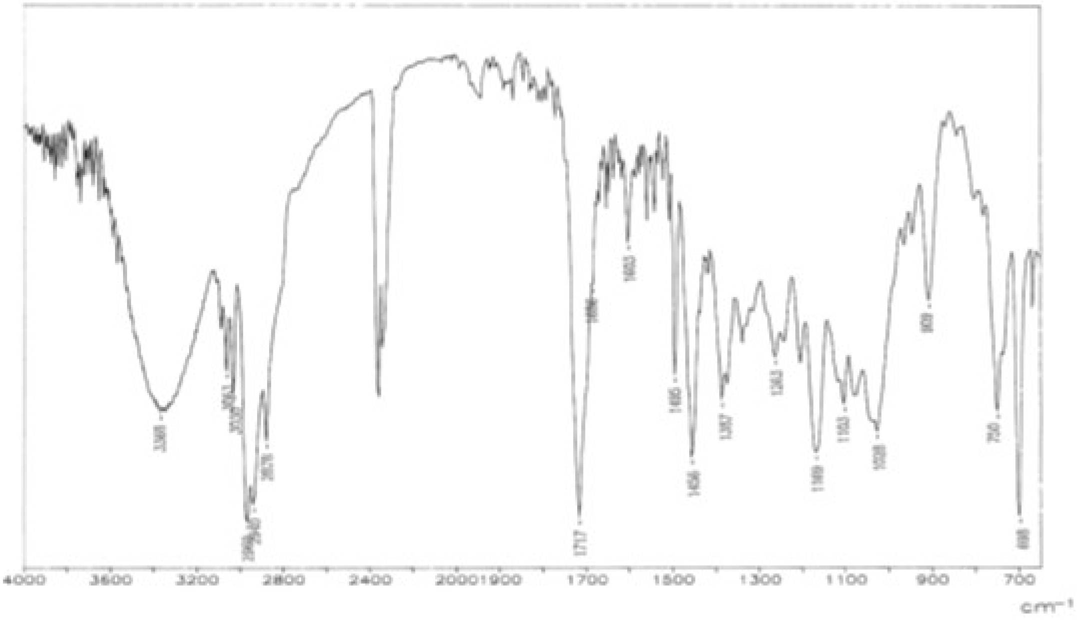

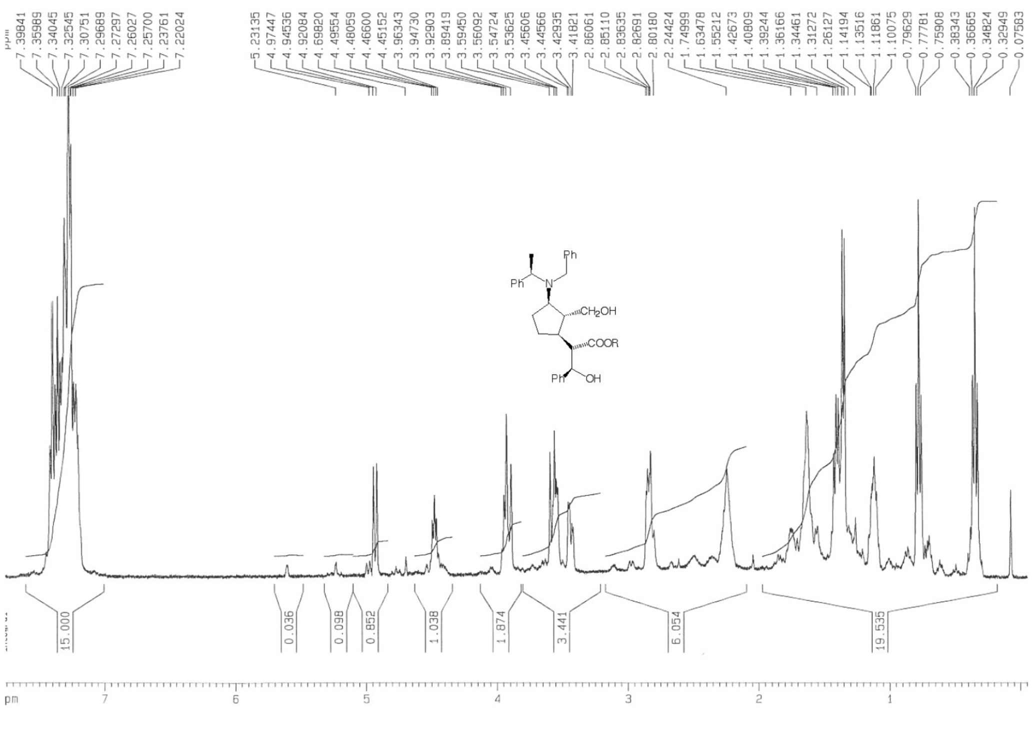

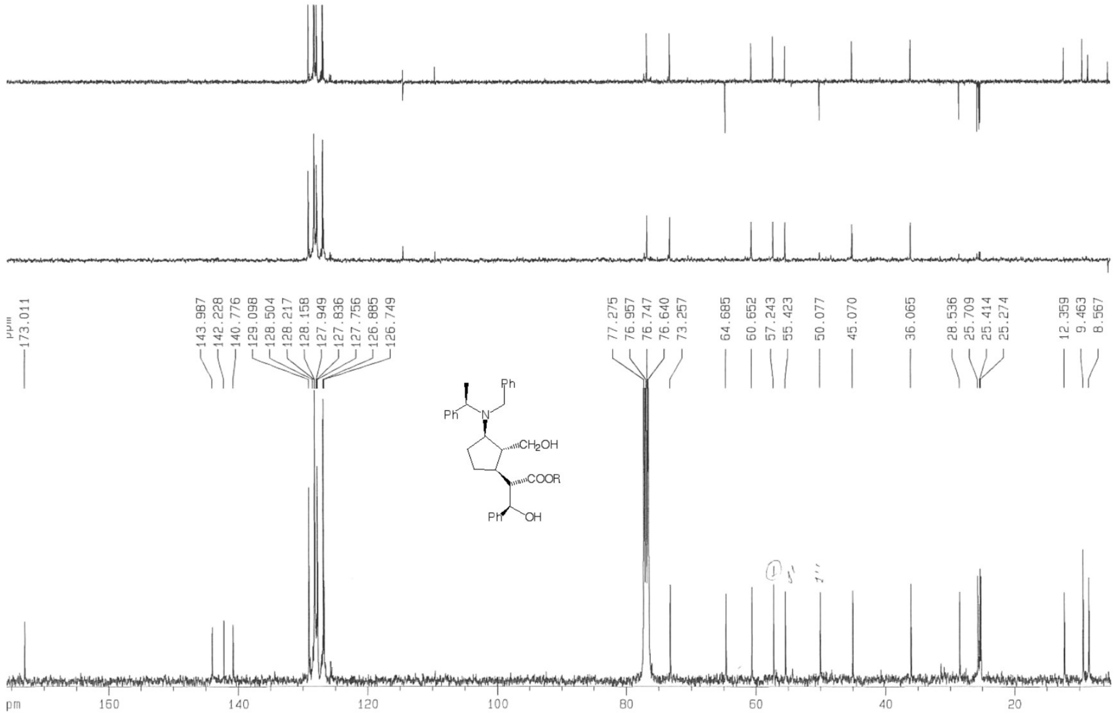
173.011
143.987
142.228
140.776
129.098
128.504
128.217
128.158
127.949
127.836
127.756
126.885
126.749
77.275
76.957
76.747
76.640
73.257
64.685
60.652
57.243
55.423
50.077
45.070
36.065
28.536
25.709
25.414
25.274
12.359
9.463
8.567
Ph
N
Ph
CH2OH
COOR
Ph
OH
160
140
120
100
80
60
40
20

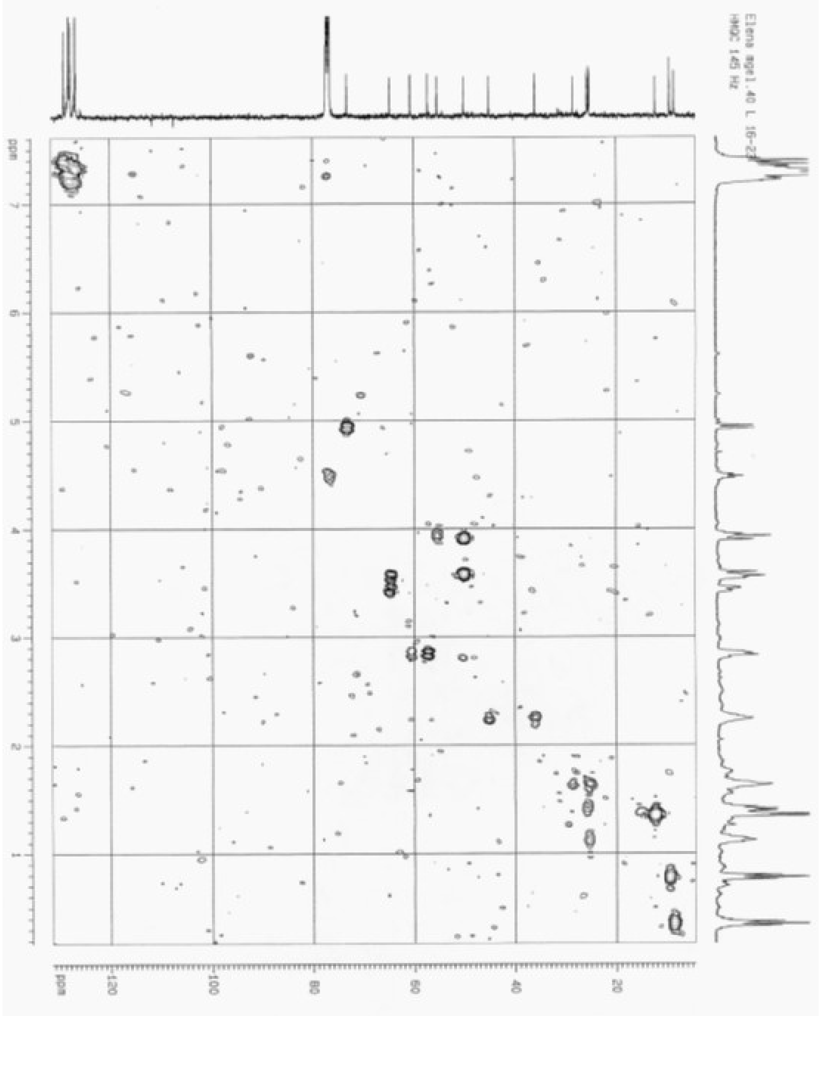
120
100
80
60
40
20

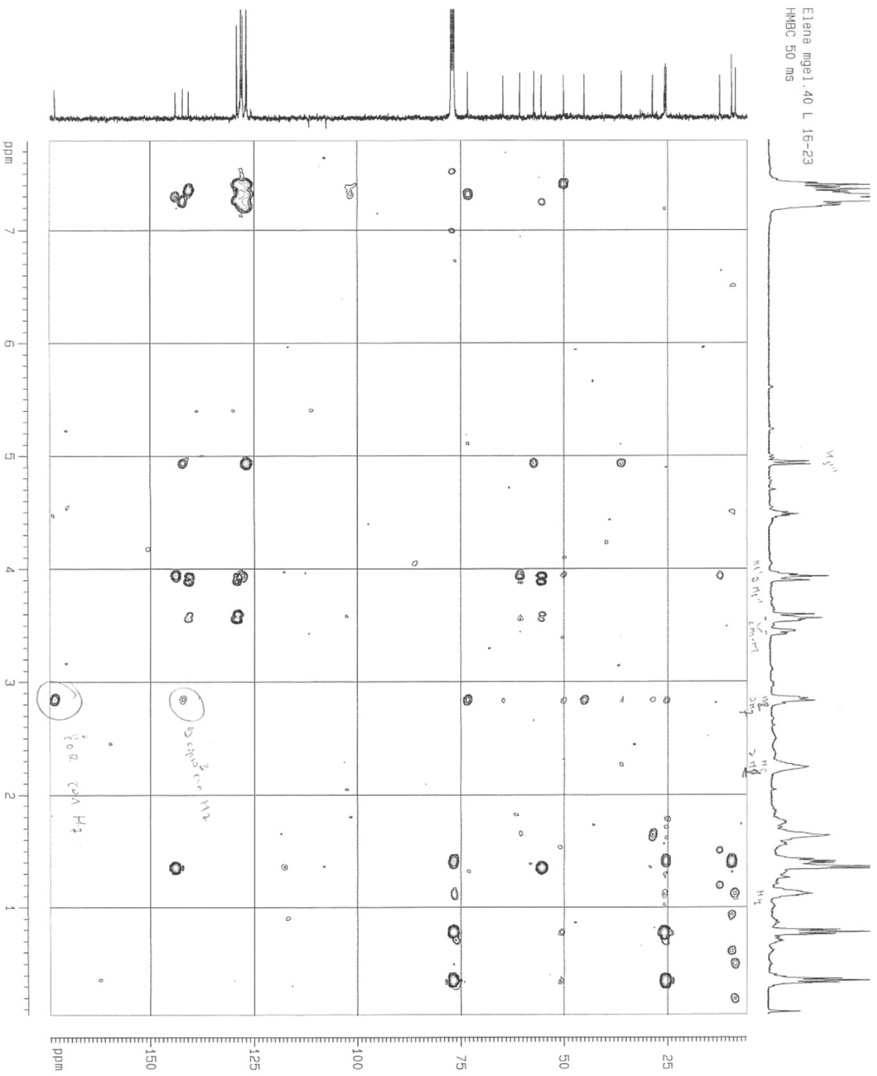
Elena mgel.40 L 16-23
HMBC 50 ms
ppm
150
125
100
75
50
25
ppm
7
6
5
4
3
2
1

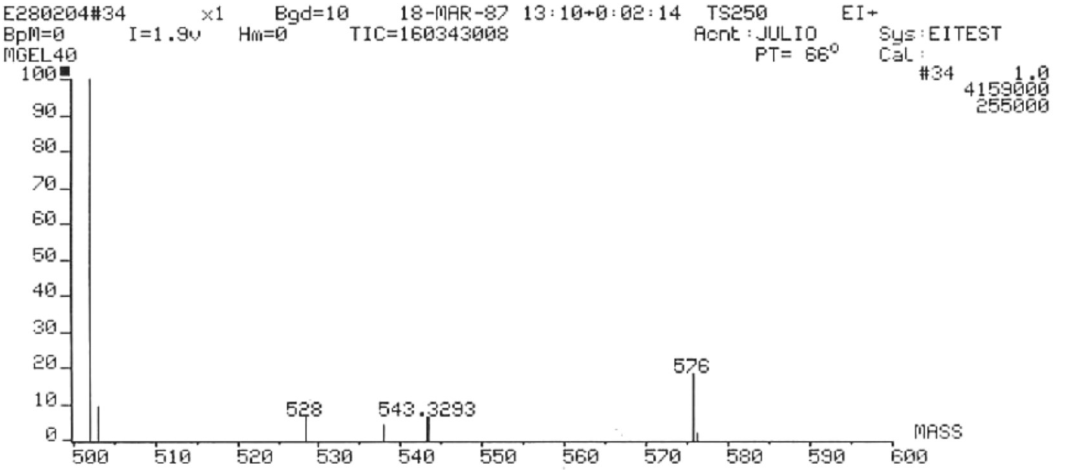
E280204#34 x1 Bgd=10 18-MAR-87 13:10+0:02:14 TS250 EI+
BpM=0 I=1.9v Hm=0 TIC=160343008 Acnt:JULIO Sys:EITEST
MGEL40 PT= 66° Cal:
#34 1.0
4159000
255000
100
90
80
70
60
50
40
30
20
10
0
576
528
543.3293
MASS
500
510
520
530
540
550
560
570
580
590
600

4. IR, ^{1}H RMN, ^{13}C RMN, HMQC, HMBC, COSY and HRMS for 6

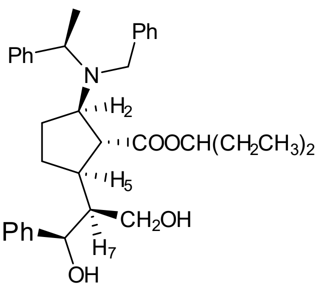

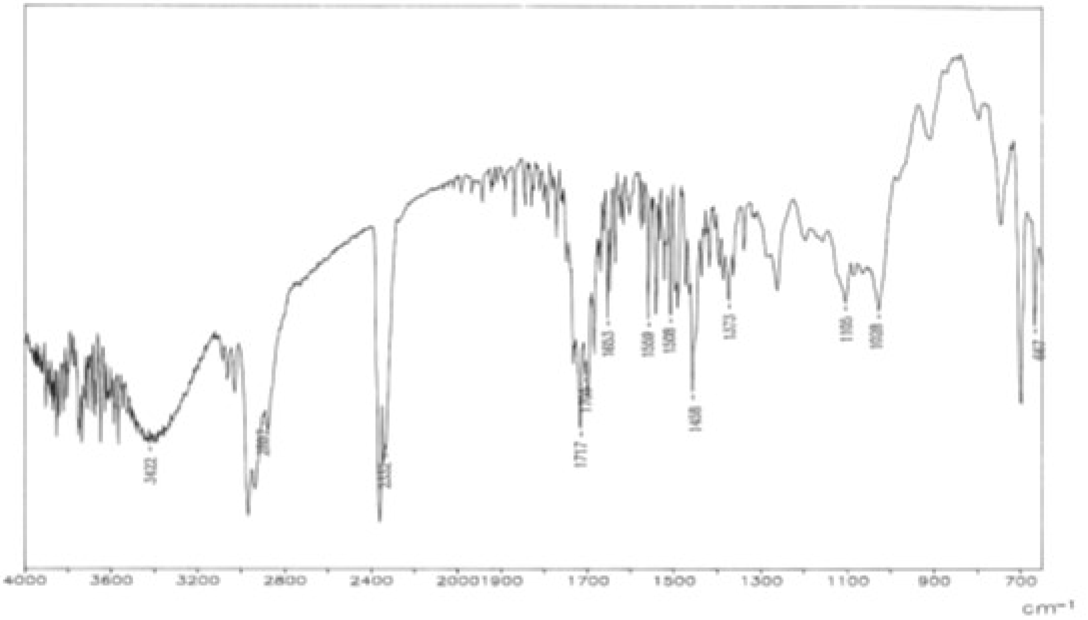

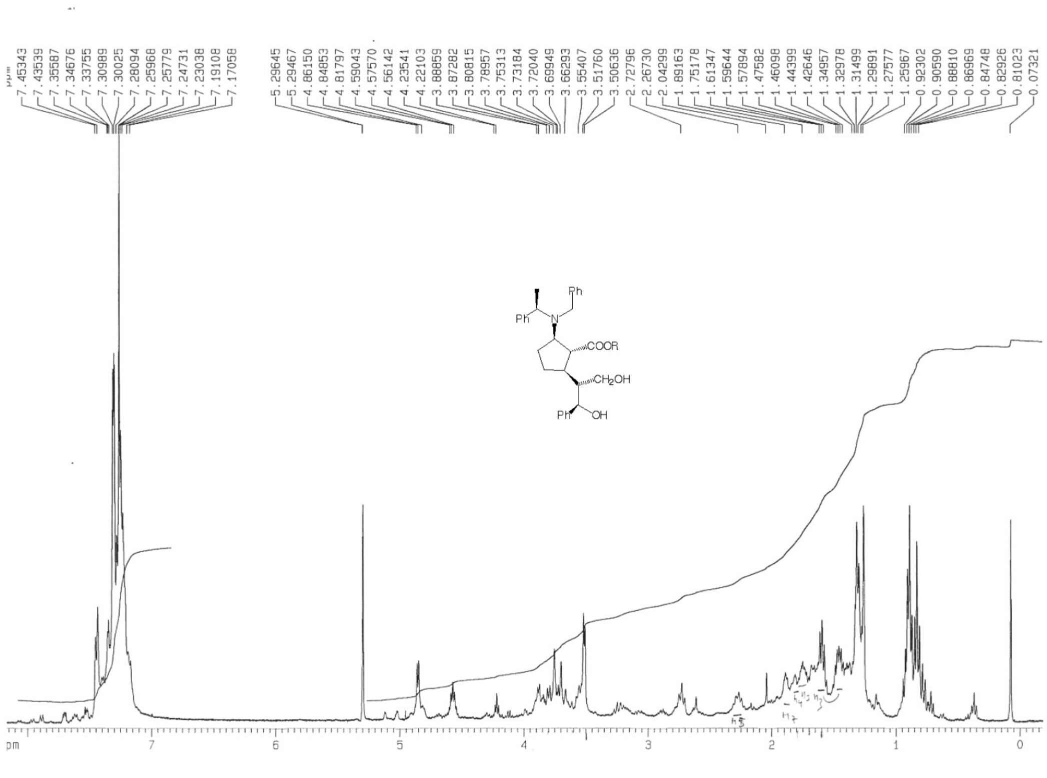

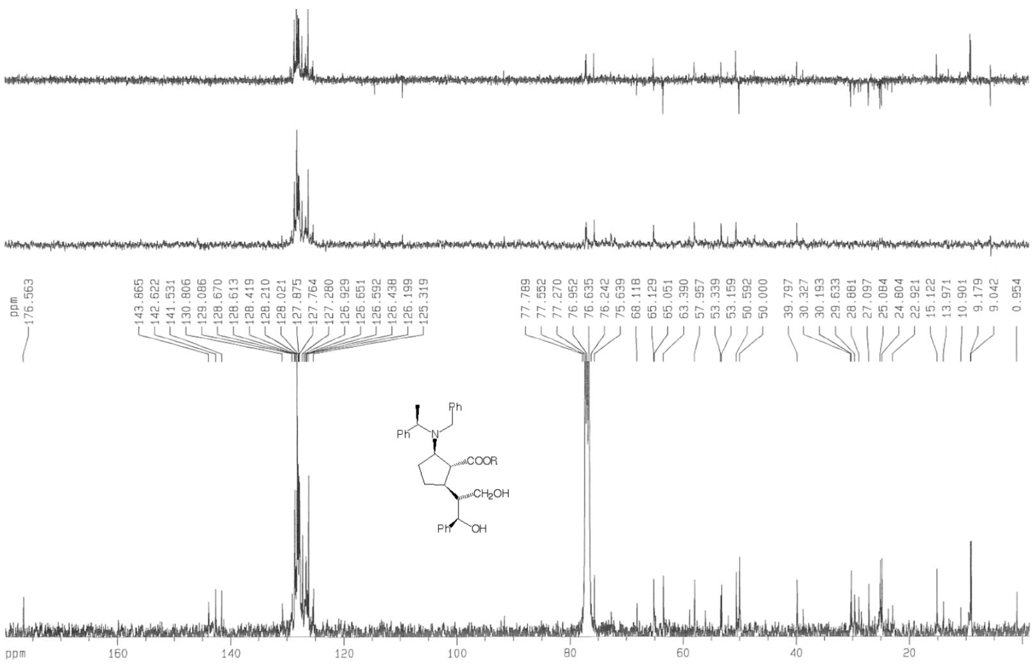
ppm
176.563
143.865
142.622
141.531
130.806
129.086
128.670
128.613
128.419
128.210
128.021
127.875
127.764
127.280
126.929
126.651
126.592
126.438
126.199
125.319
77.789
77.552
77.270
76.952
76.635
76.242
75.639
68.118
65.129
65.051
63.390
57.957
53.339
53.159
50.592
50.000
39.797
30.327
30.193
29.633
28.881
27.097
25.084
24.804
22.921
15.122
13.971
10.901
9.179
9.042
0.954
Ph
N
Ph
COOR
CH2OH
Ph
OH
ppm 160 140 120 100 80 60 40 20

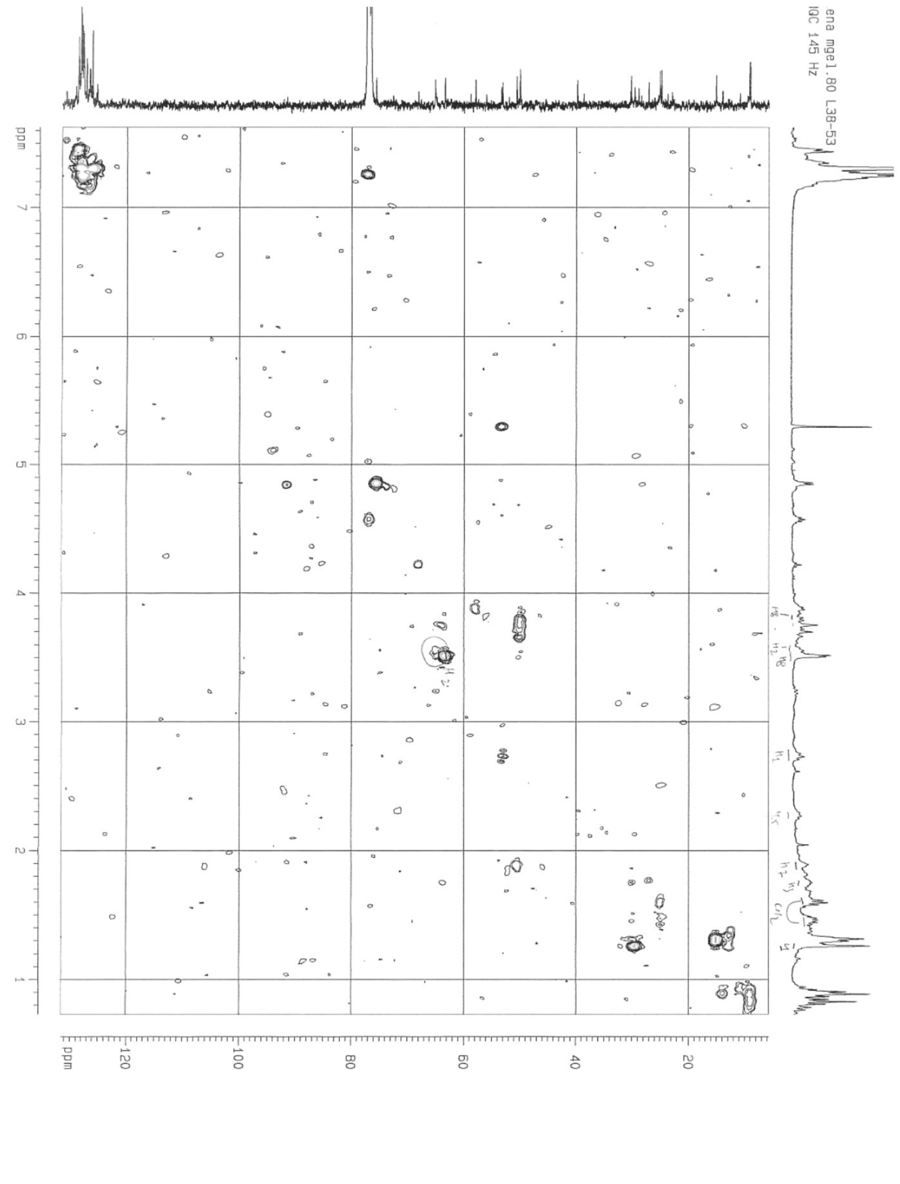
ena mge1.80 L38-53
IQC 145 Hz
ppm 120 100 80 60 40 20
ppm 7 6 5 4 3 2 1

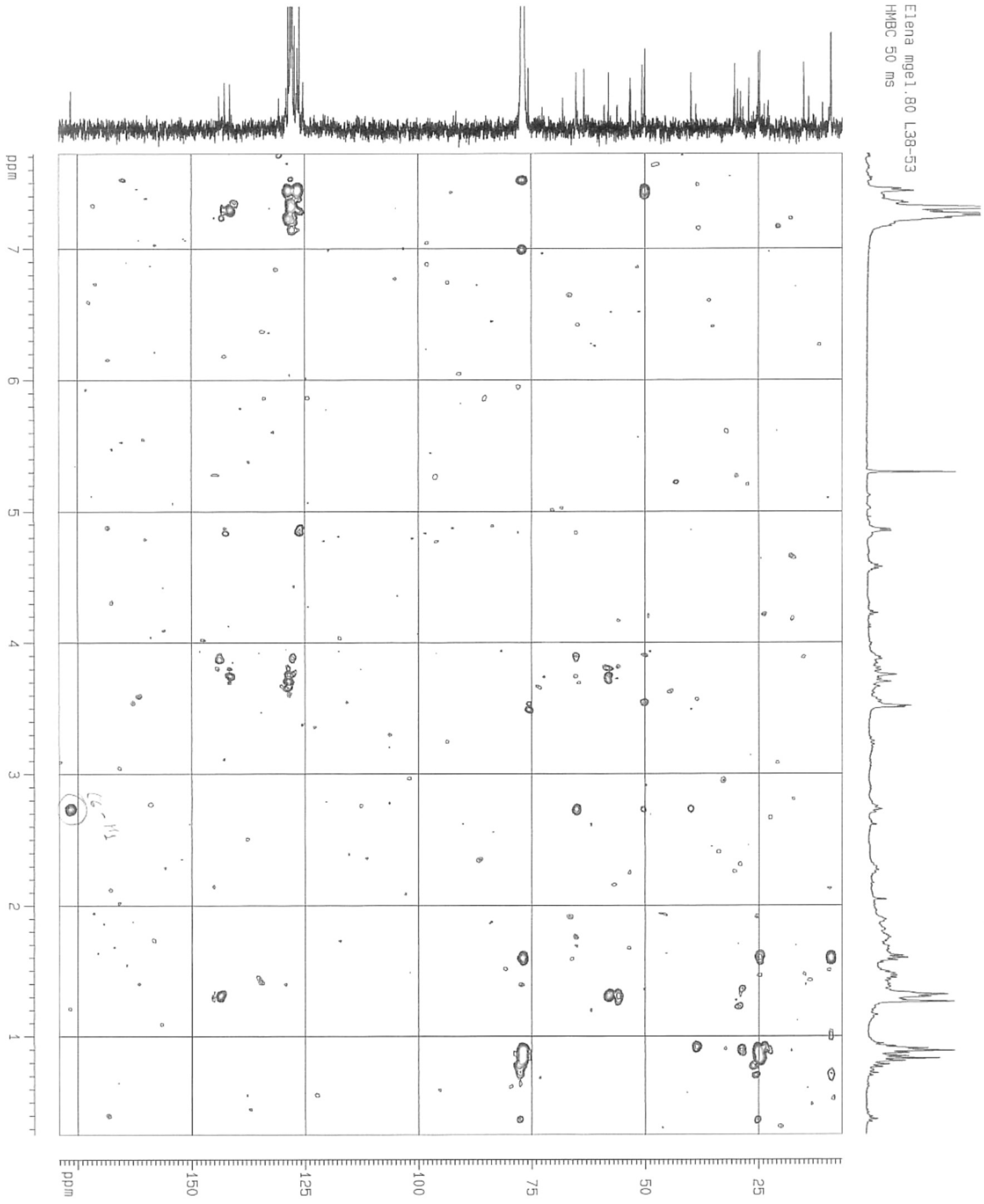
Elena mge1.80 L38-53
HMBC 50 ms
ppm
ppm

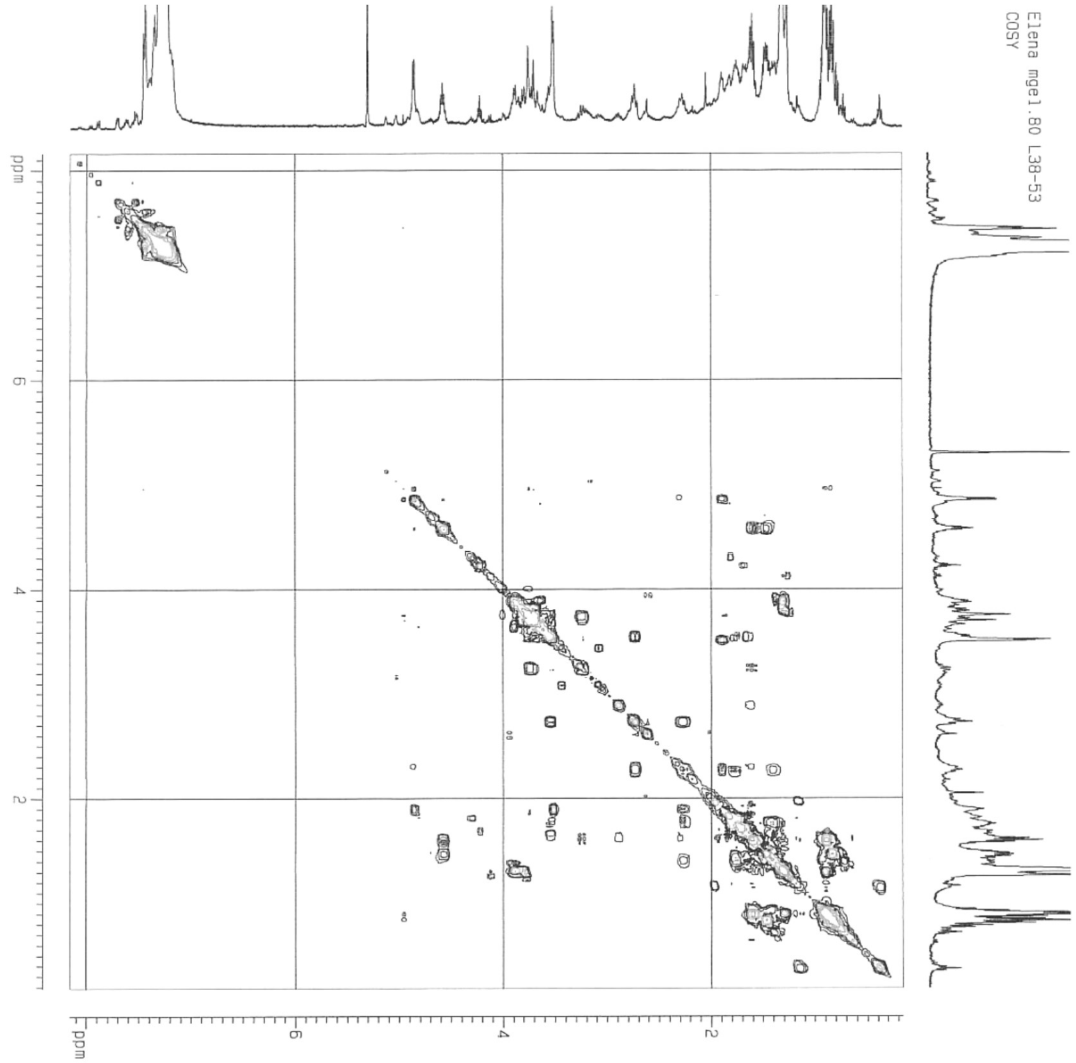
Elena mge1.80 L38-53
COSY
ppm
6
4
2
ppm
6
4
2

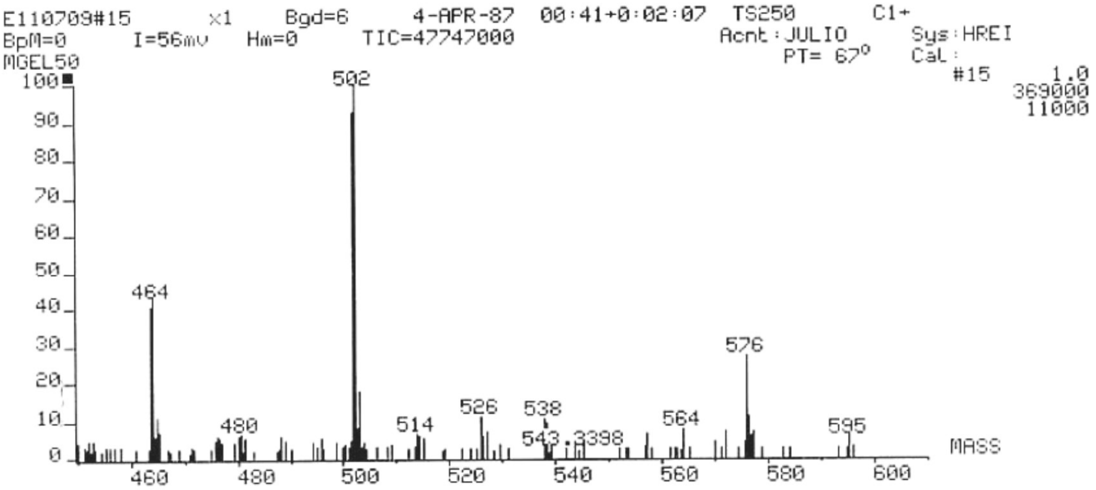
E110709#15 ×1 Bgd=6 4-APR-87 00:41+0:02:07 TS250 CI+
BpM=0 I=56mv Hm=0 TIC=47747000 Acnt:JULIO Sys:HREI
MGEL50 PT= 67° Cal:
#15 1.0
369000
11000
100
90
80
70
60
50
40
30
20
10
0
502
464
480
514
526
538
543.3398
564
576
595
460
480
500
520
540
560
580
600
MASS

5. **IR, ¹H RMN, ¹³C RMN and HRMS for 7**

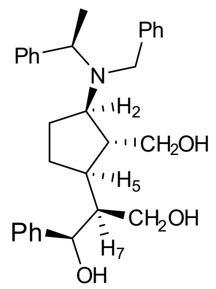

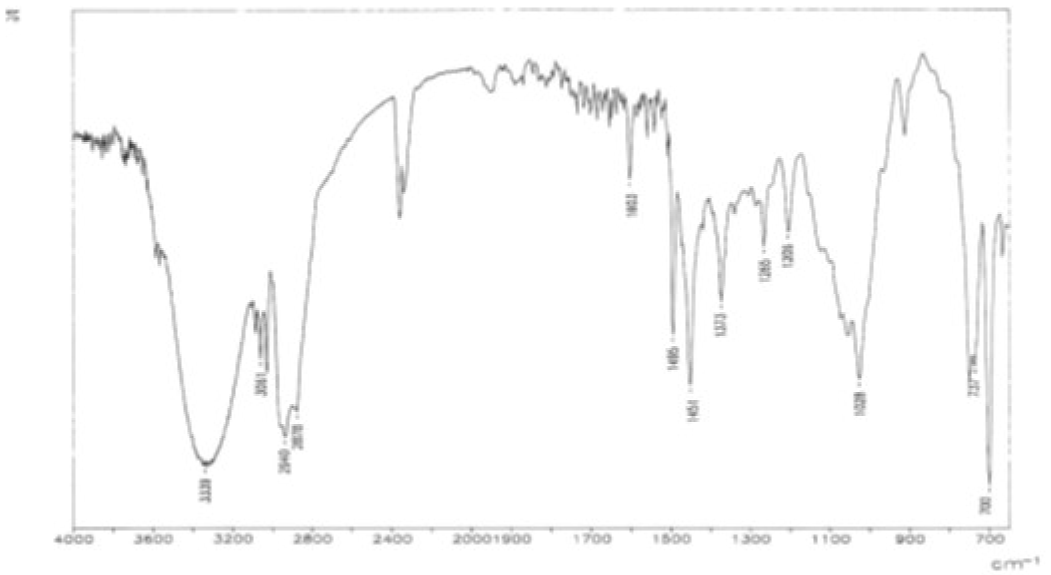

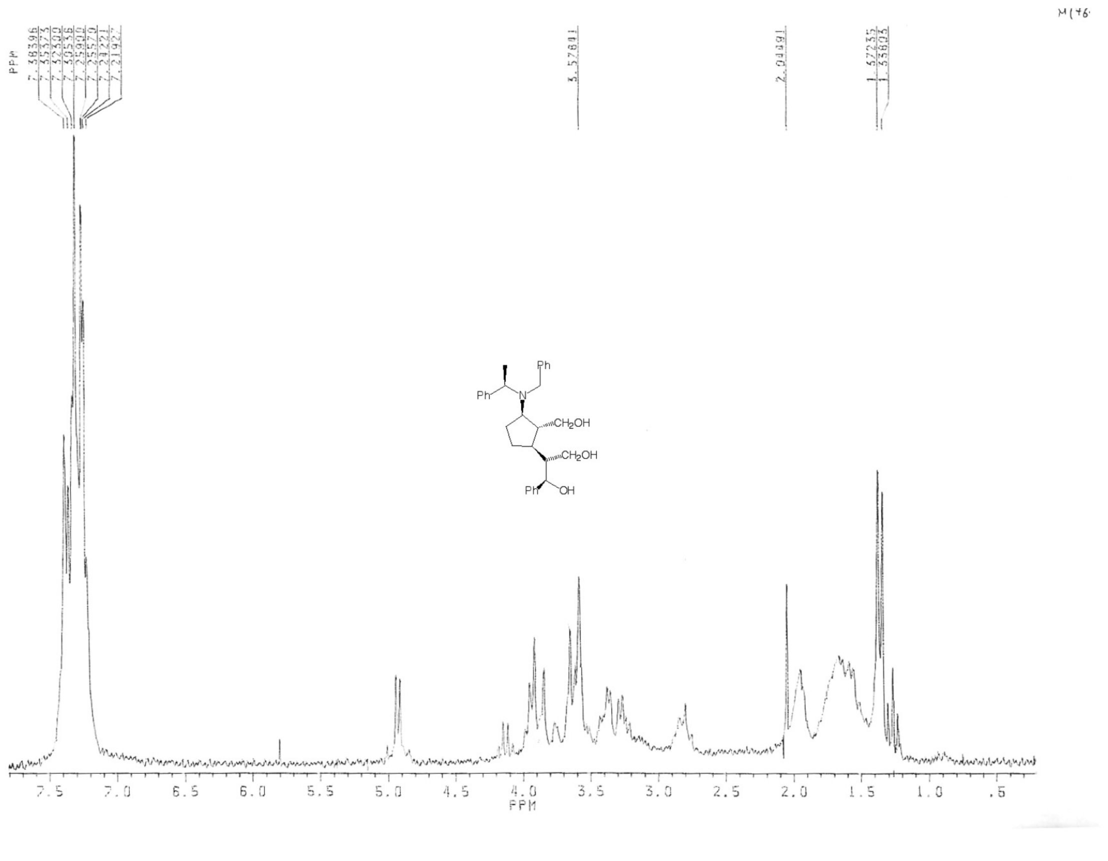

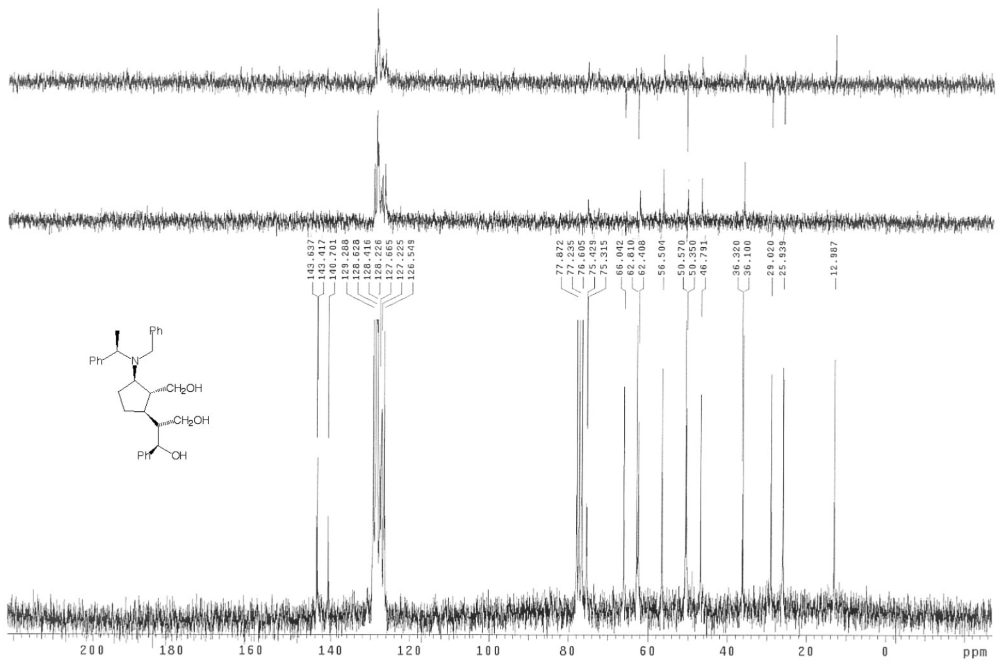
143.637
143.417
140.701
129.208
128.628
128.416
128.226
127.665
127.225
126.549
77.872
77.235
76.605
75.429
75.315
66.042
62.810
62.408
56.504
50.570
50.350
46.791
36.320
36.100
29.020
25.939
12.987
Ph
Ph
N
CH2OH
CH2OH
Ph
OH
200
180
160
140
120
100
80
60
40
20
0
ppm

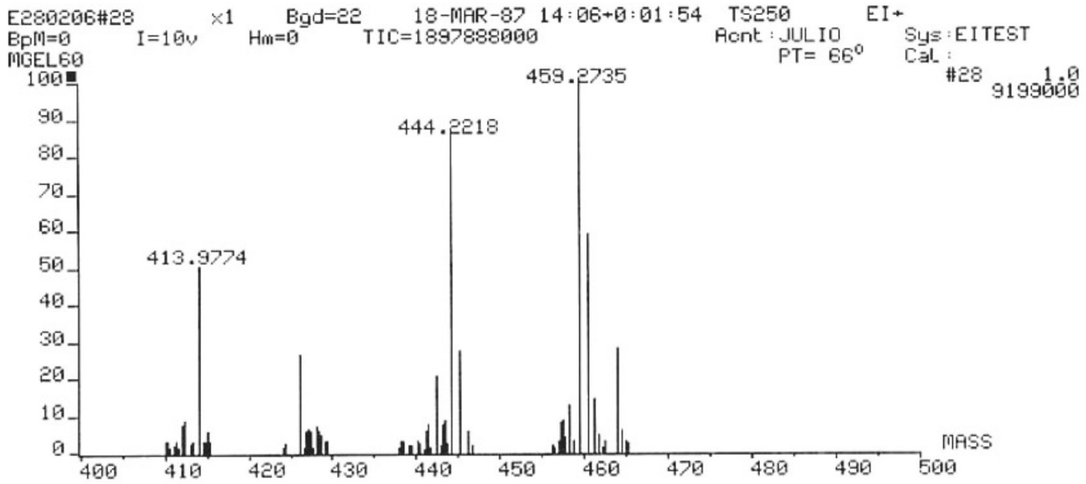
E280206#28 ×1 Bgd=22 18-MAR-87 14:06+0:01:54 TS250 EI+
BpM=0 I=10v Hm=0 TIC=1897888000 Acnt:JULIO Sys:EITEST
MGEL60 PT= 66° Cal:
#28 1.0
9199000
459.2735
444.2218
413.9774
MASS

6. IR, ^{1}H RMN and ^{13}C RMN for 8

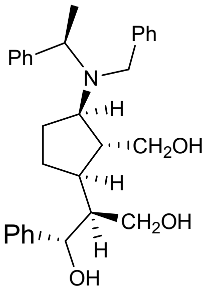

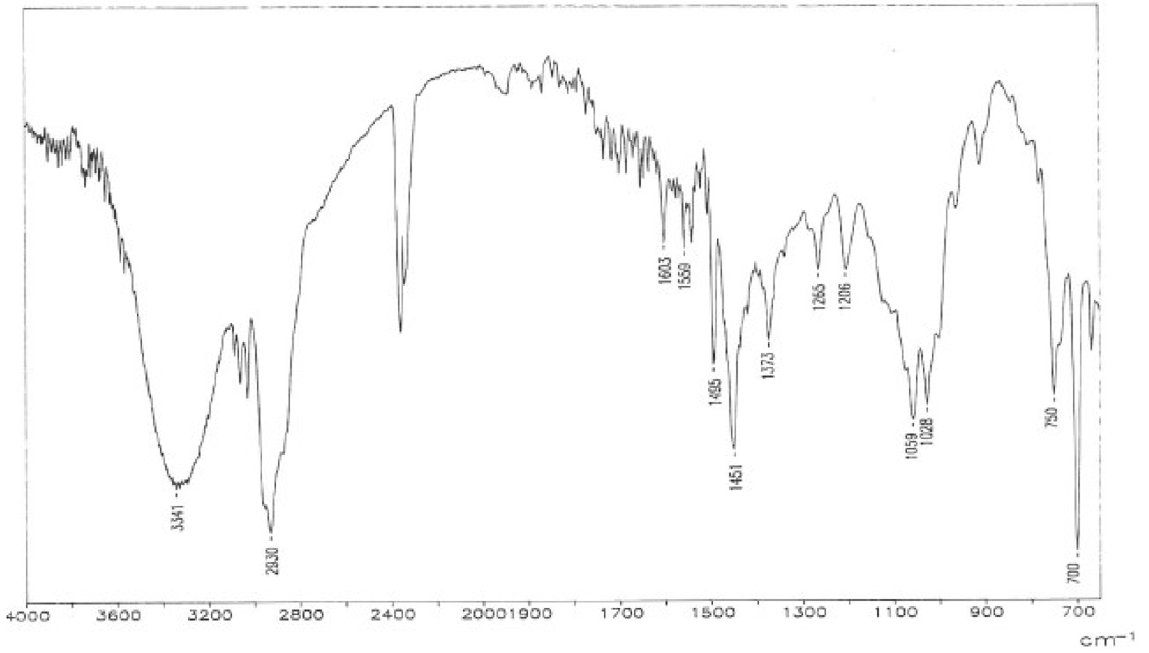

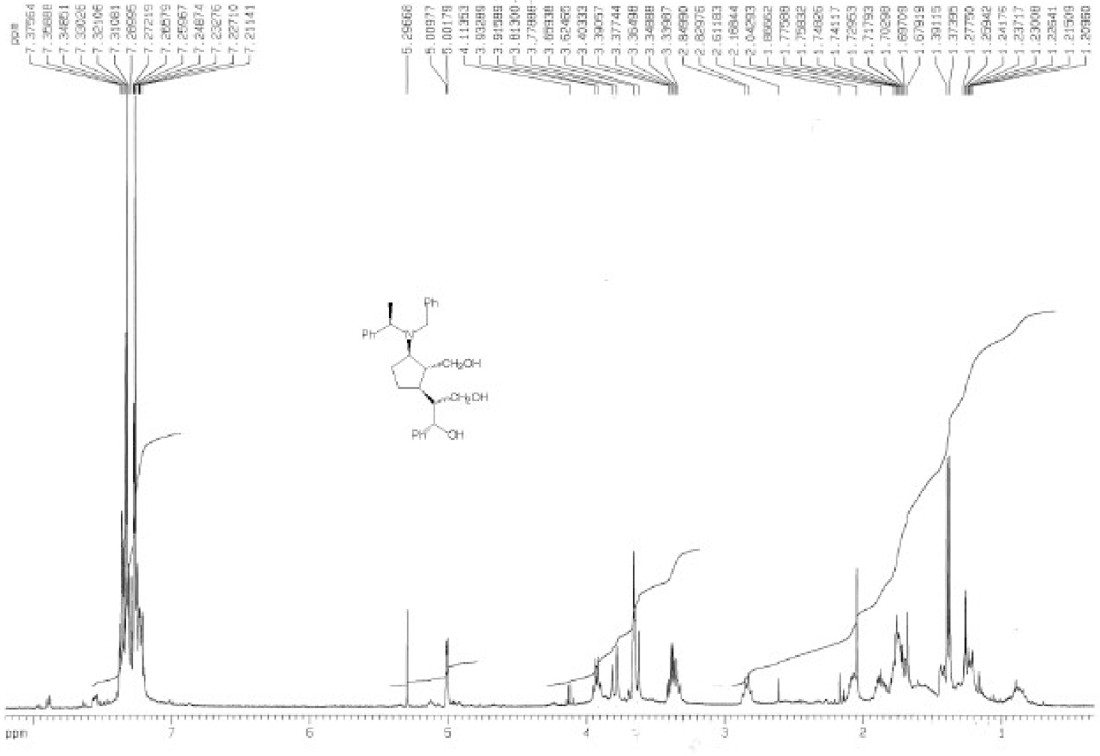

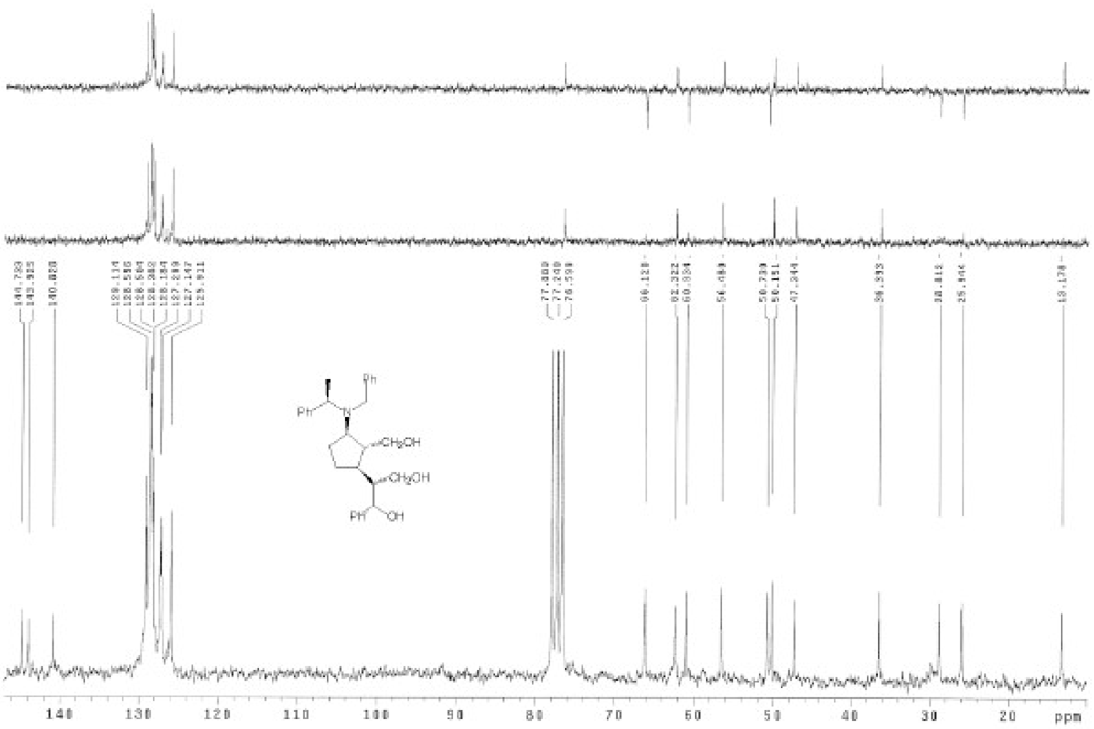
Ph
Ph
N
CH2OH
CH2OH
Ph
OH
140
130
120
110
100
90
80
70
60
50
40
30
20
ppm

7. IR, ^{1}H RMN, ^{13}C RMN, ROESY, COSY and HRMS for 9

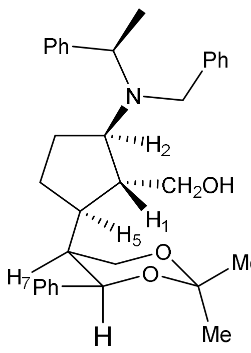

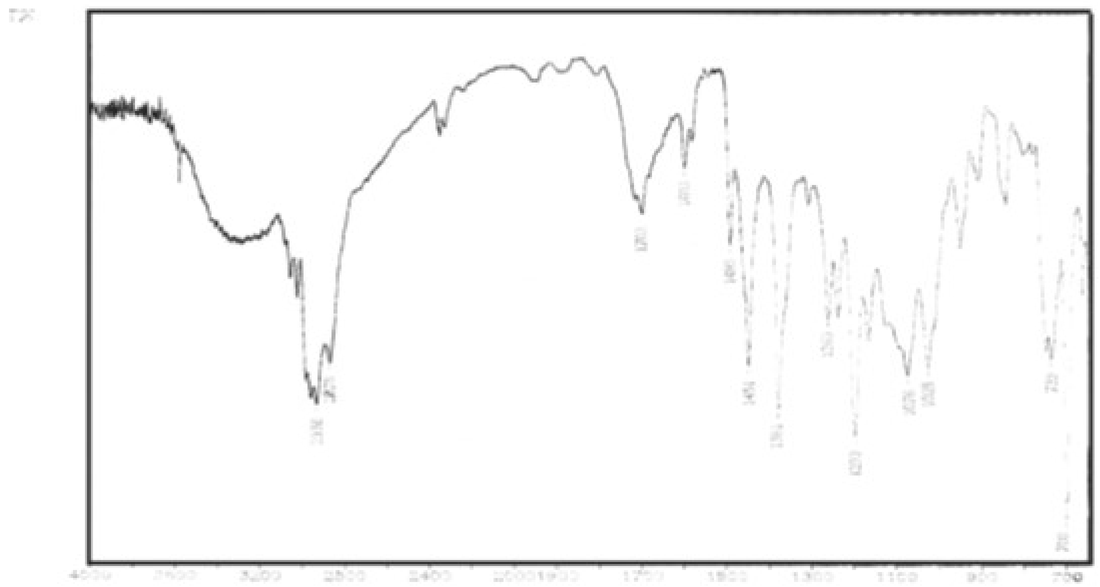

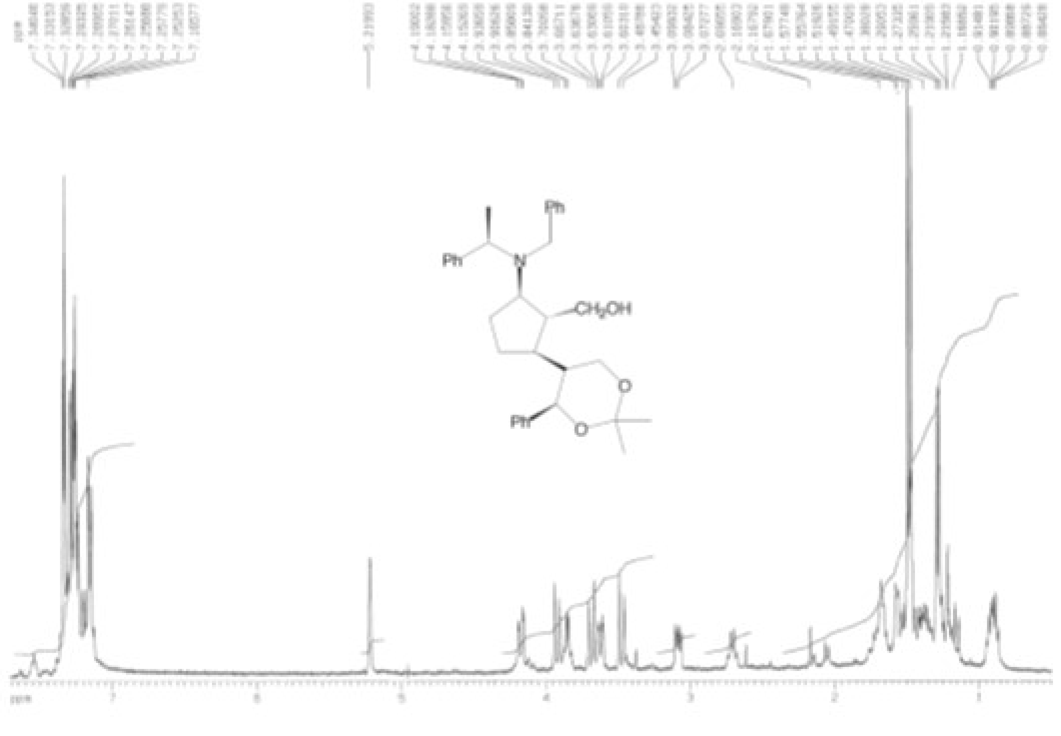

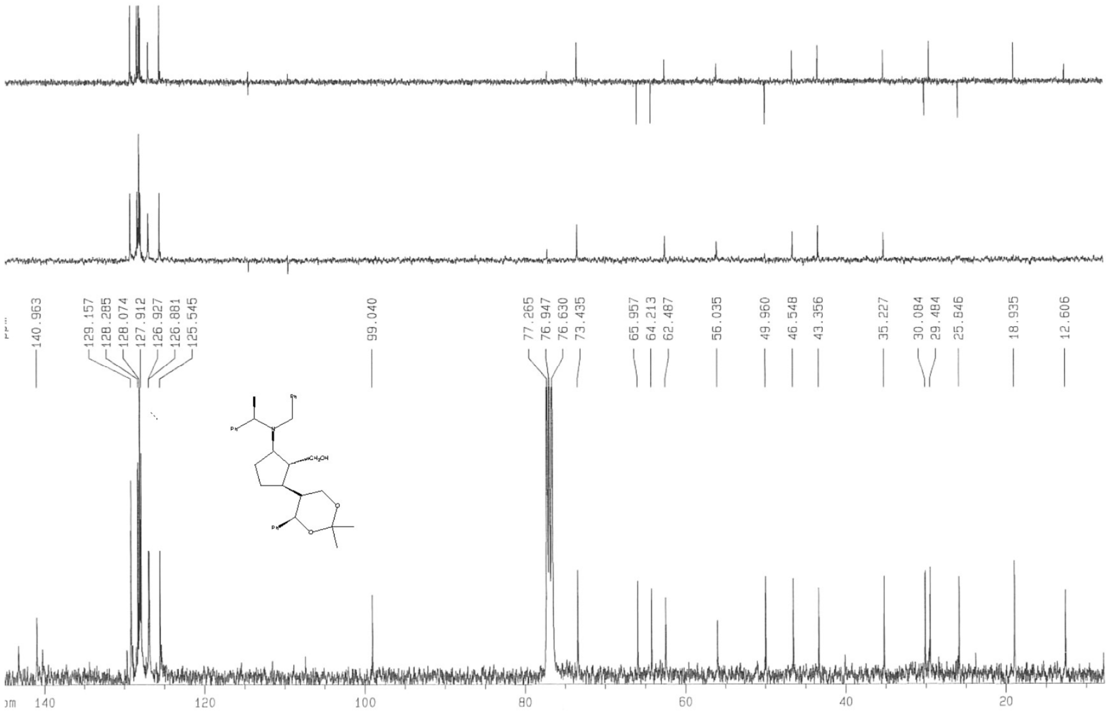
140.963
129.157
128.285
128.074
127.912
126.927
126.881
125.545
99.040
77.265
76.947
76.630
73.435
65.957
64.213
62.487
56.035
49.960
46.548
43.356
35.227
30.084
29.484
25.846
18.935
12.606

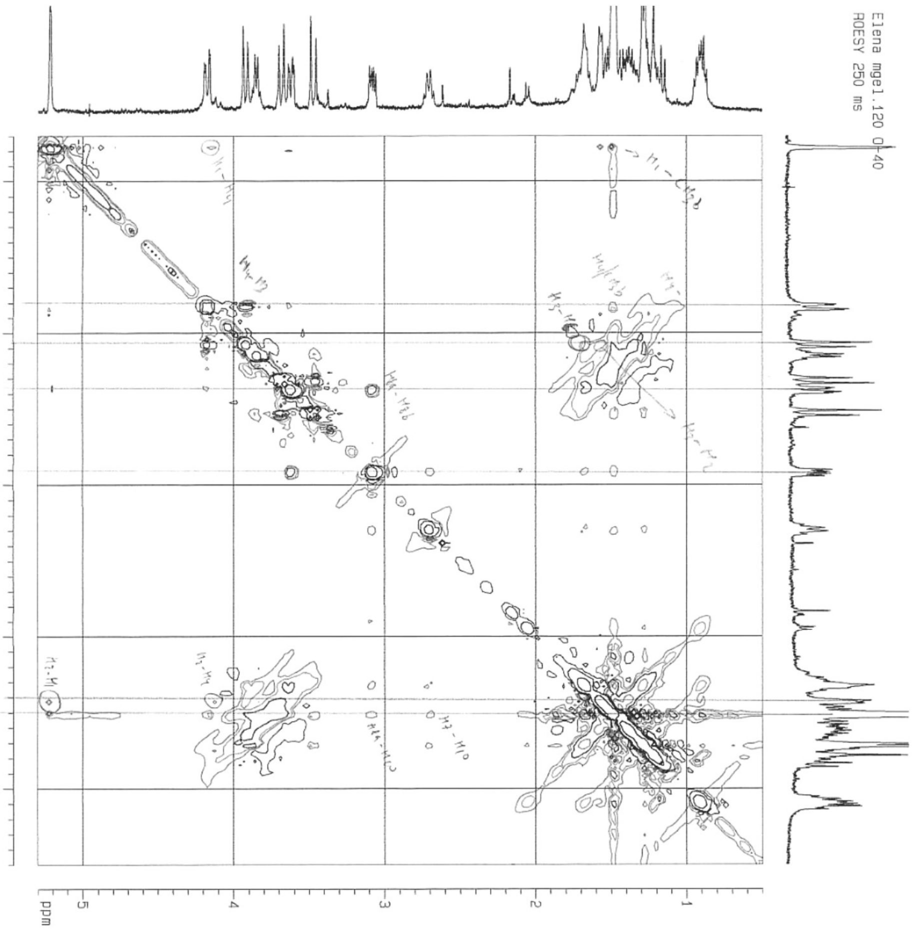
Elena mge1.120 0 40
ROESY 250 ms

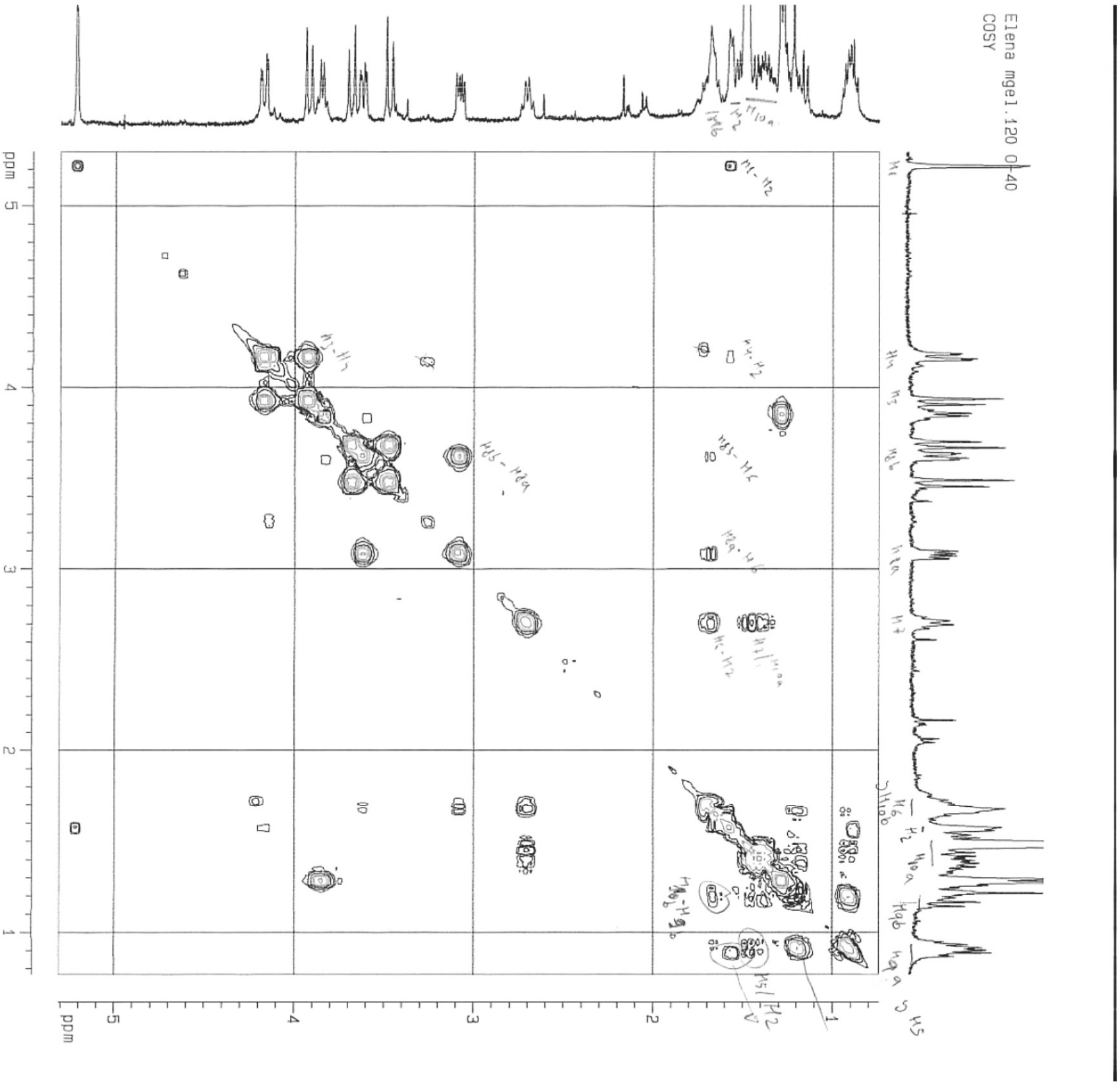

Elena mge1.120 0-40
COSY
ppm

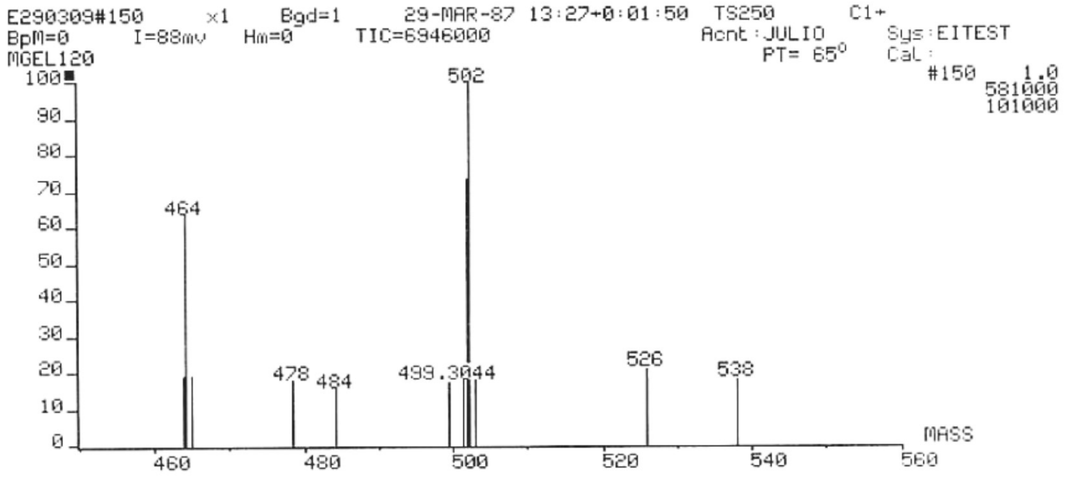

E290309#150 x1 Bgd=1 29-MAR-87 13:27+0:01:50 TS250 C1+
BpM=0 I=88mv Hm=0 TIC=6946000 Acnt:JULIO Sys:EITEST
MGEL120 PT= 65° Cal:
#150 1.0
581000
101000
502
464
478
484
499.3044
526
538
MASS

8. ^{1}H RMN, ^{13}C RMN and HRMS for 10

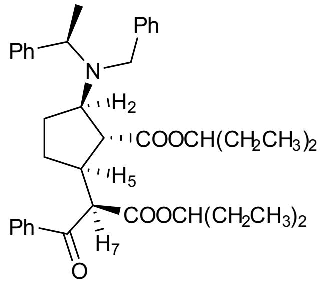

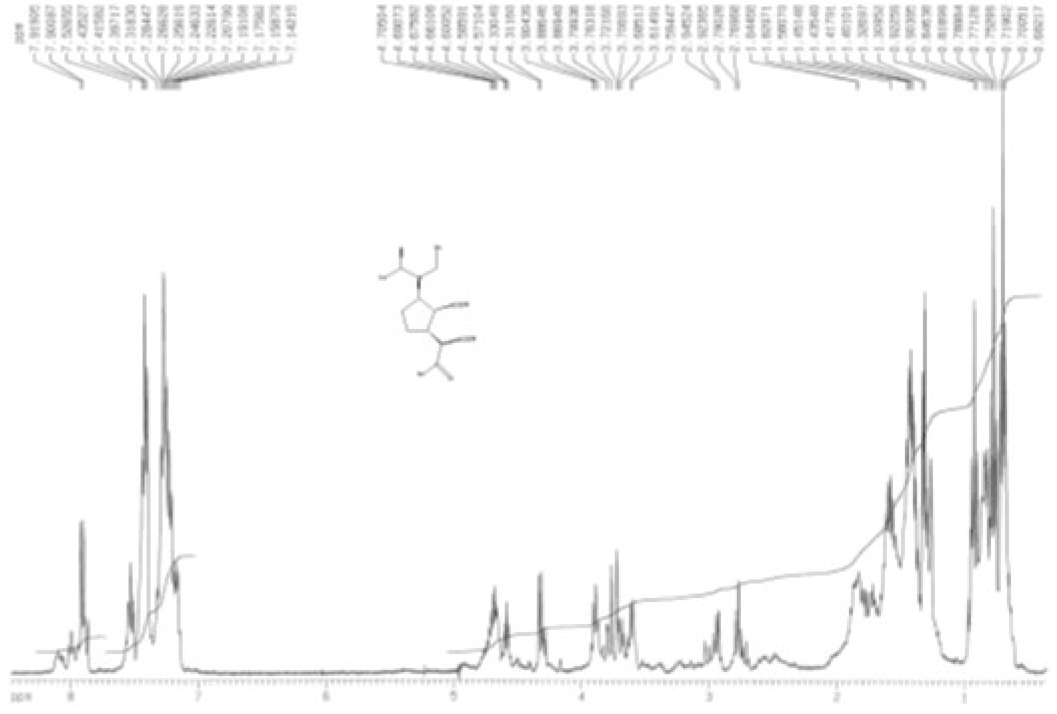

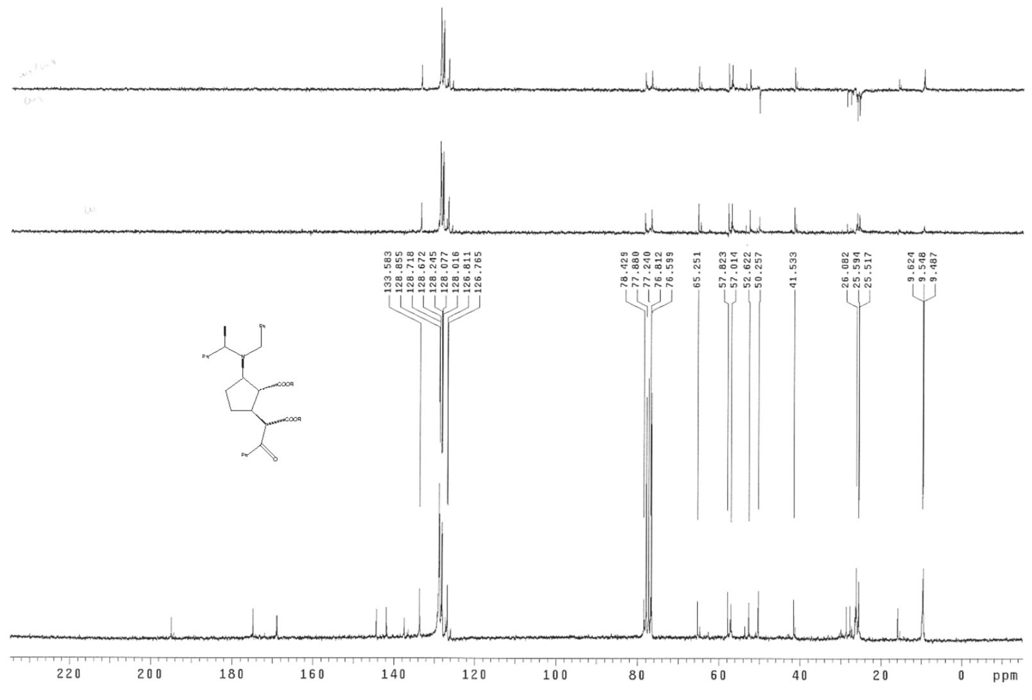

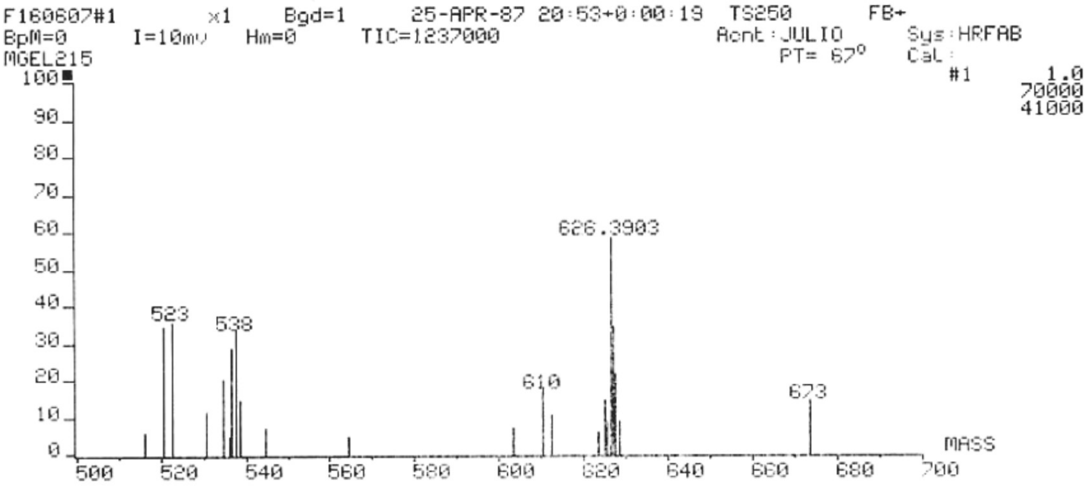

F160607#1 x1 Bgd=1 25-APR-87 20:53+0:00:19 TS250 FB+
BpM=0 I=10mv Hm=0 TIC=1237000 Acnt:JULIO Sys:HRFAB
MGEL215 PT= 67° Cal:
#1 1.0
70000
41000
626.3903
523
538
610
673
MASS

9. IR, ^{1}H RMN and ^{13}C RMN for 11

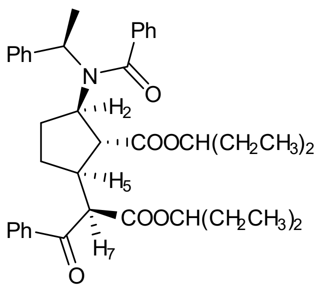

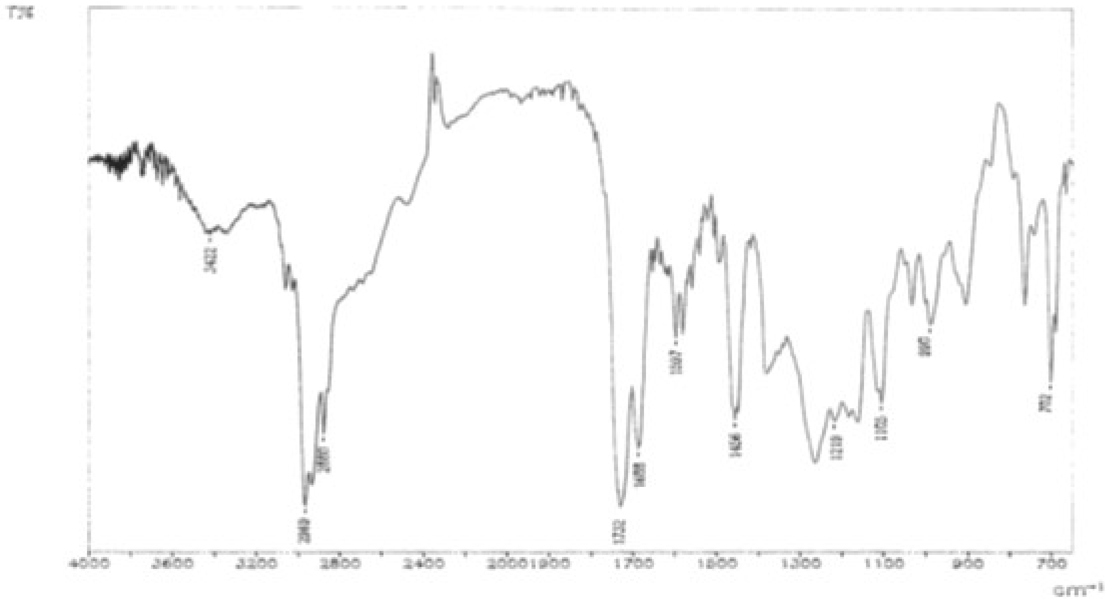

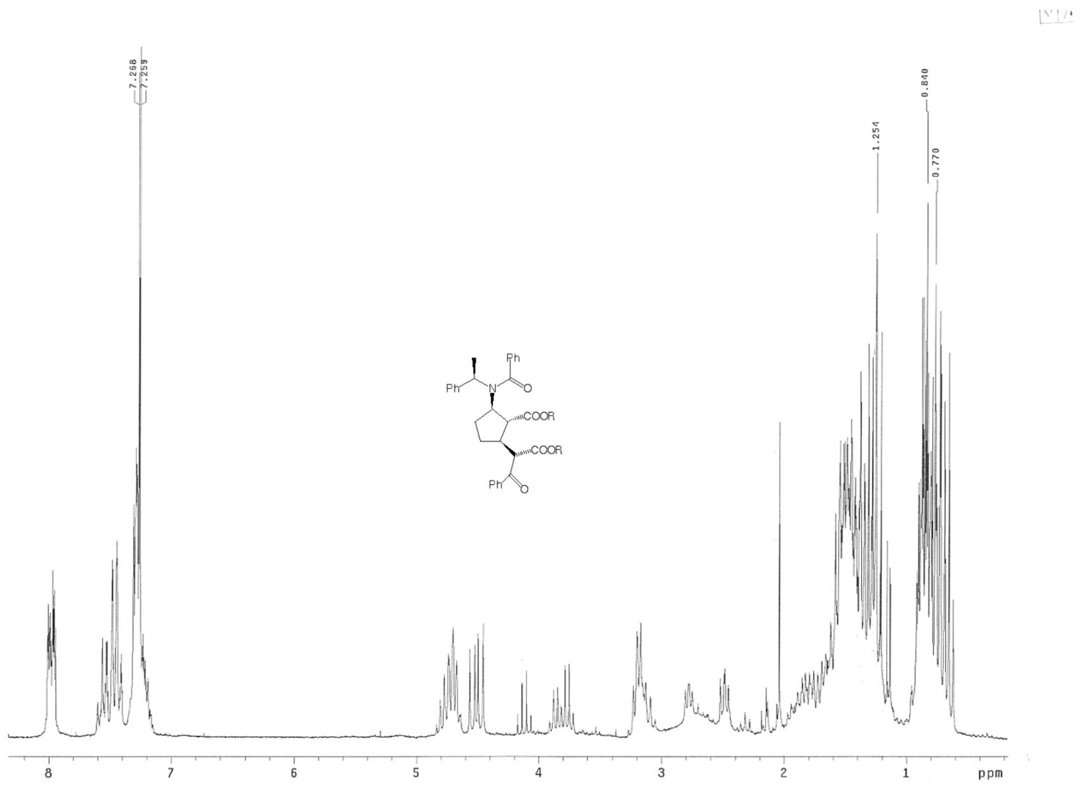

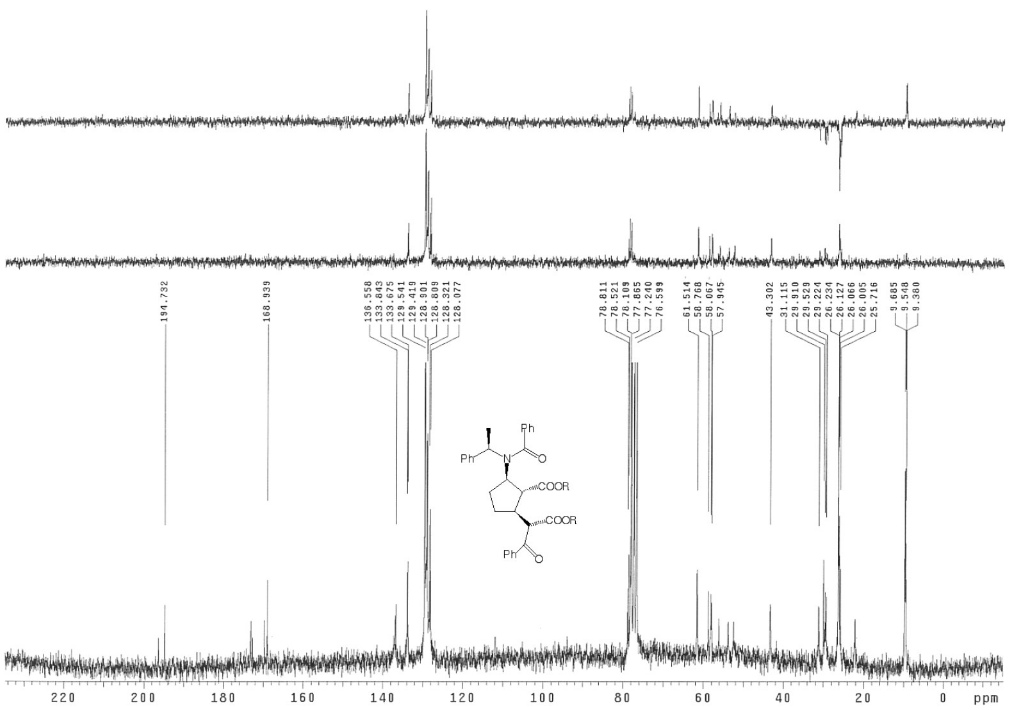

10. IR, ^{1}H RMN, ^{13}C RMN and HRMS for 12

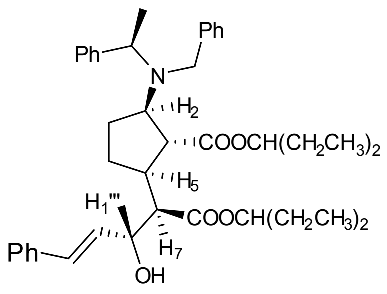

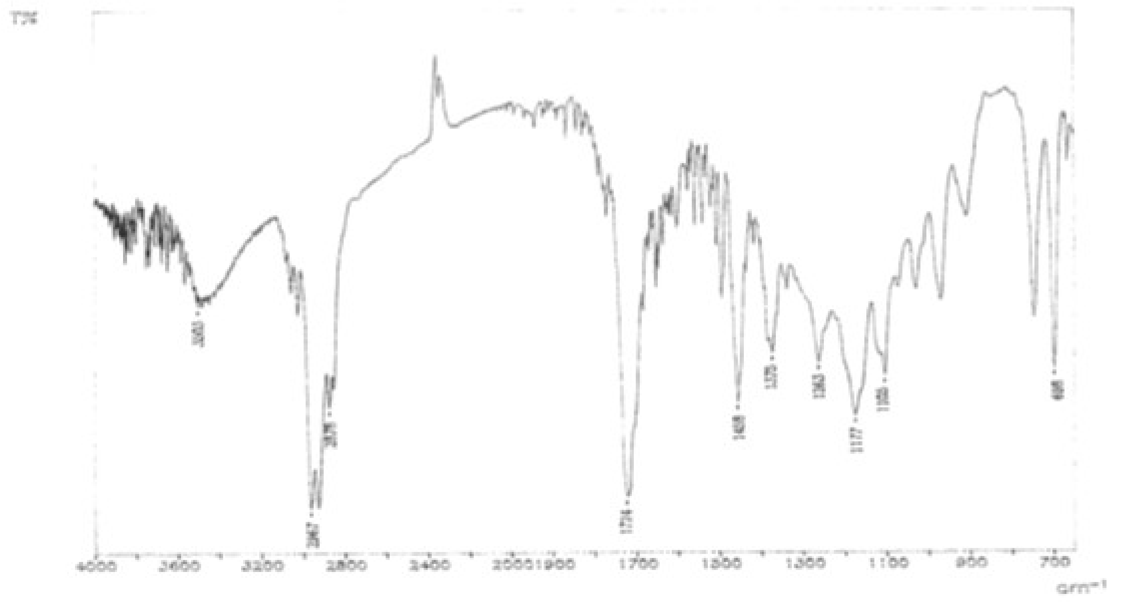

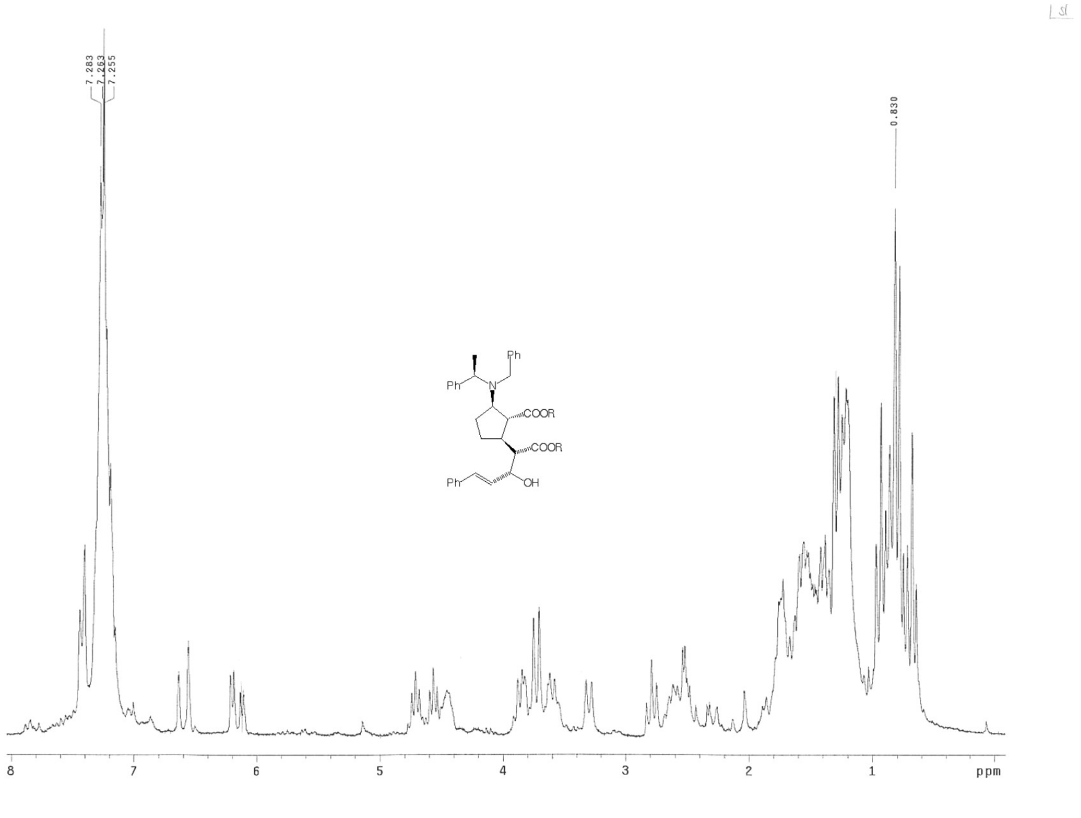

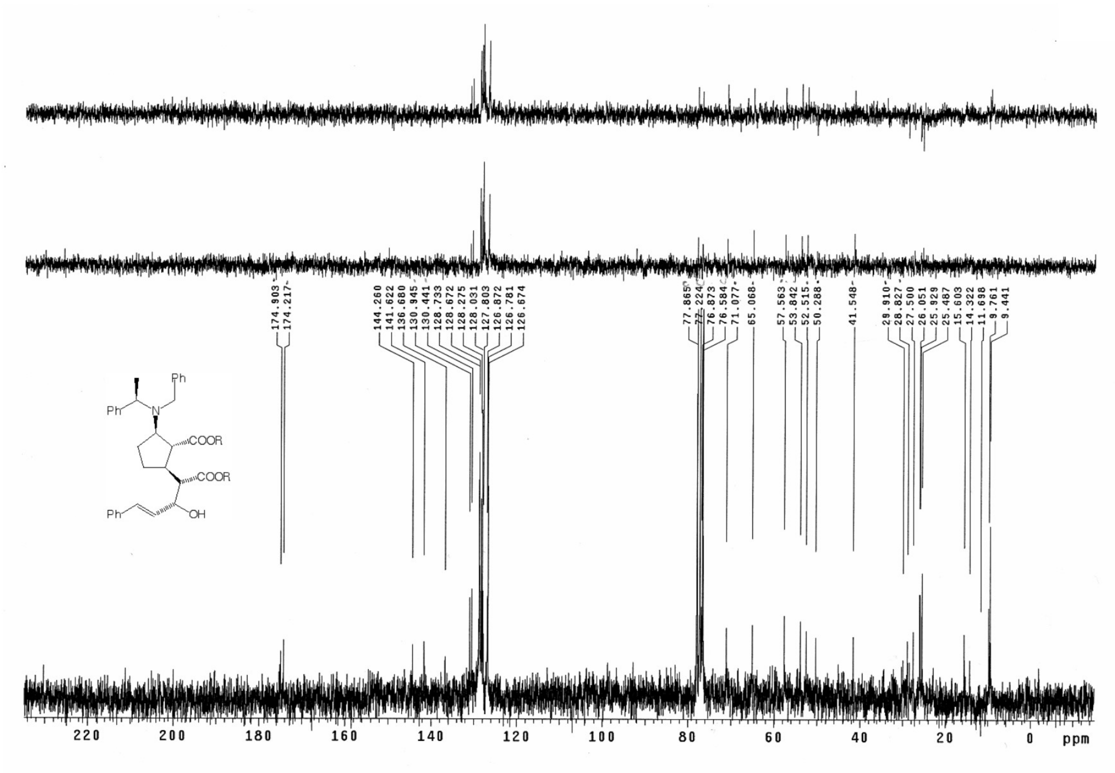
174.903
174.217
144.260
141.622
136.680
130.945
130.441
128.733
128.672
128.275
128.031
127.803
126.872
126.781
126.674
77.865
77.224
76.873
76.584
71.077
65.068
57.563
53.842
52.515
50.288
41.548
29.910
28.827
27.500
26.051
25.929
25.487
15.603
14.322
9.761
9.441
Ph
Ph
N
COOR
COOR
Ph
OH
220 200 180 160 140 120 100 80 60 40 20 0 ppm

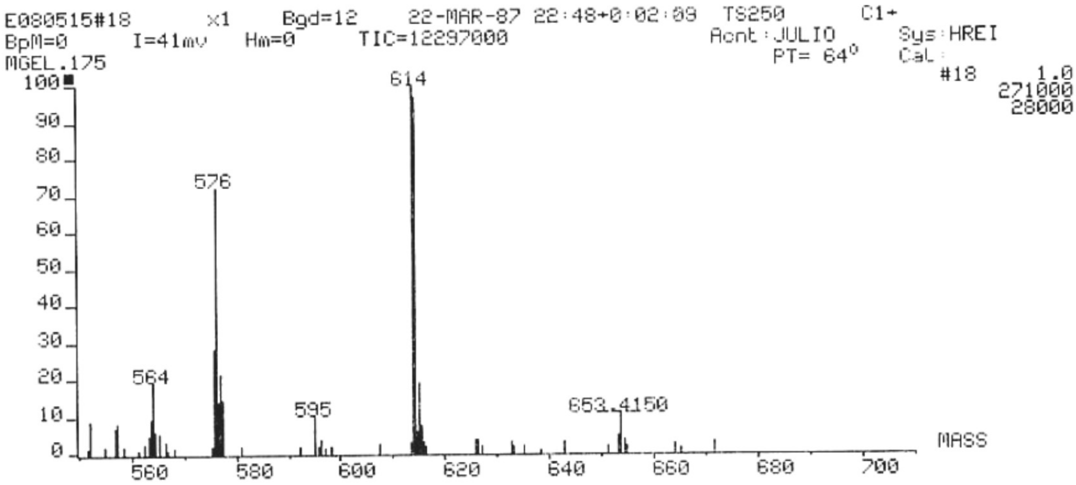
E080515#18 x1 Bgd=12 22-MAR-87 22:48+0:02:09 TS250 C1+
BpM=0 I=41mv Hm=0 TIC=12297000 Acnt:JULIO Sys:HREI
MGEL.175 PT= 64° Cal:
#18 1.0
271000
28000
614
576
564
595
653.4150
MASS
100 90 80 70 60 50 40 30 20 10 0
560 580 600 620 640 660 680 700

11. IR and ^{1}H RMN for 13

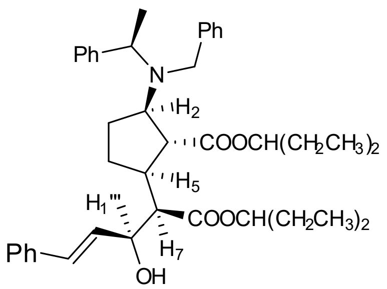

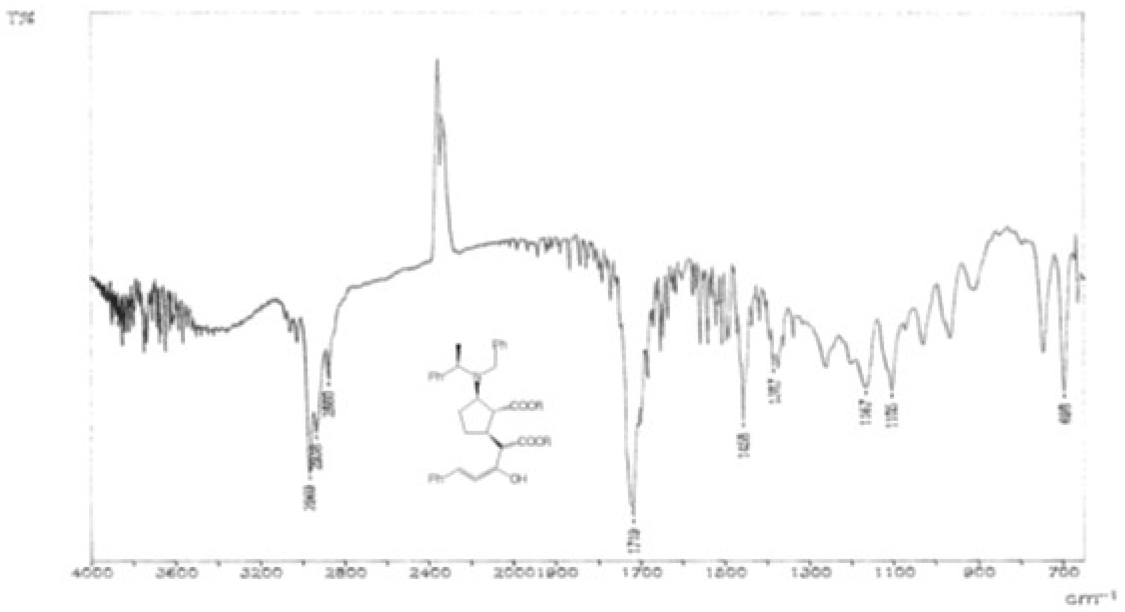

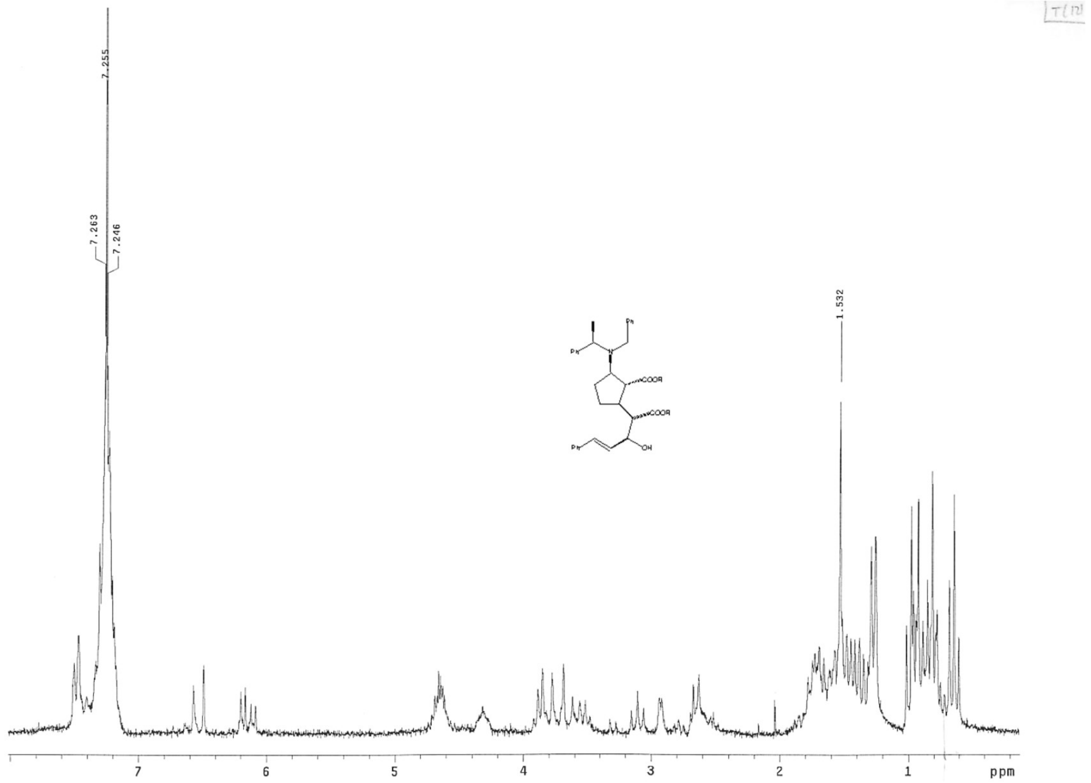

12. IR, ^{1}H RMN, ^{13}C RMN and HRMS for 14

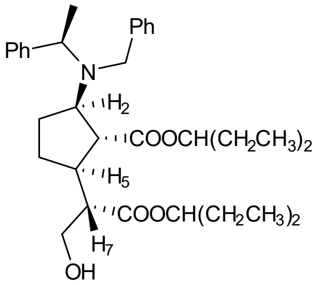

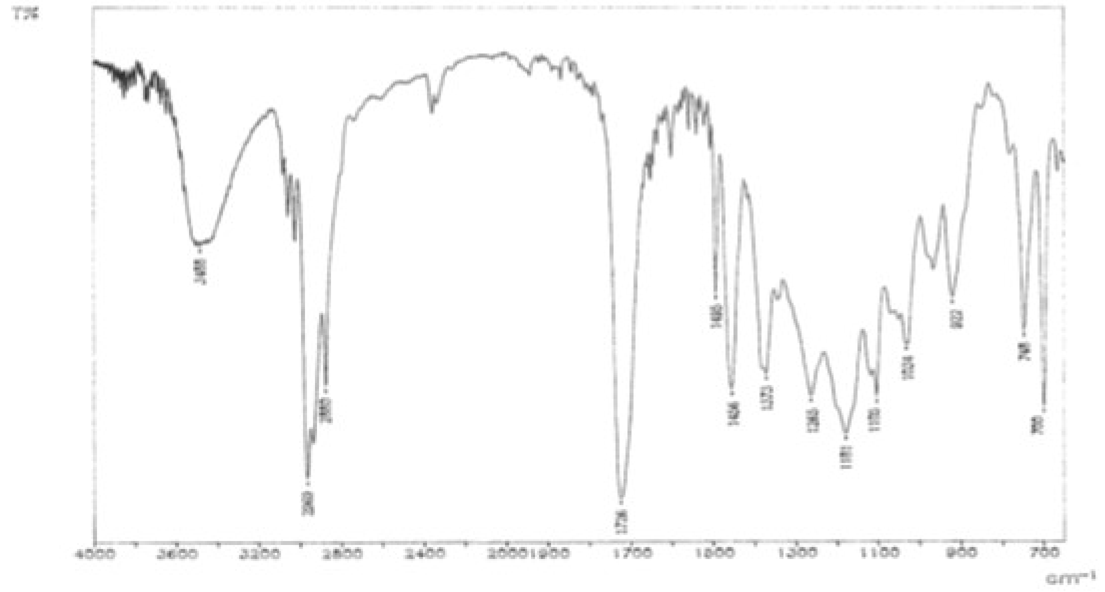

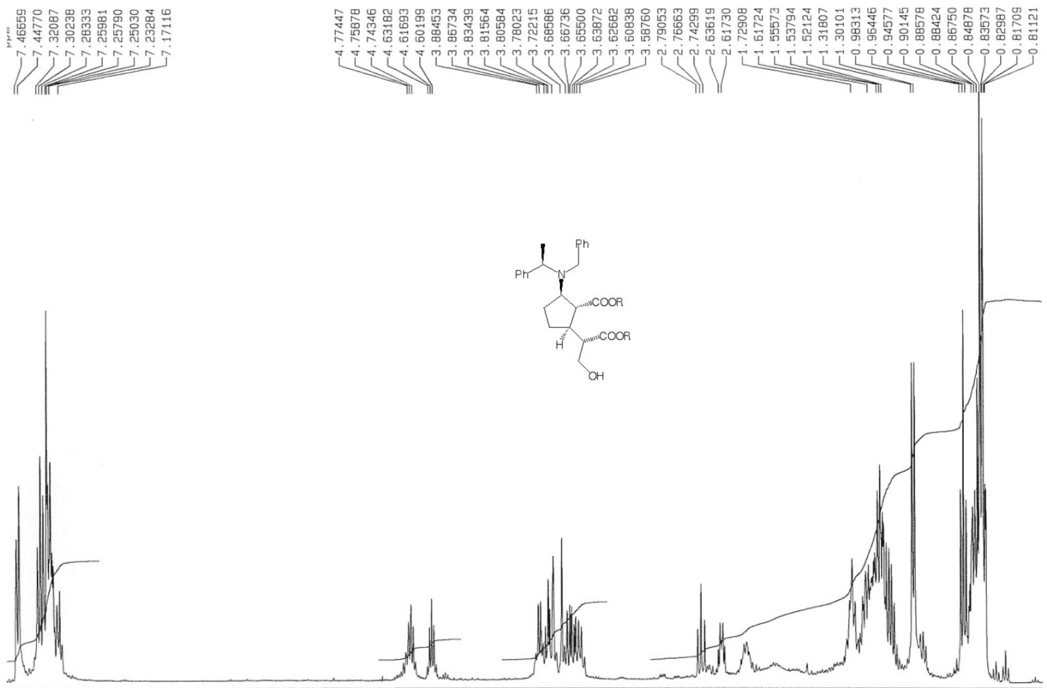

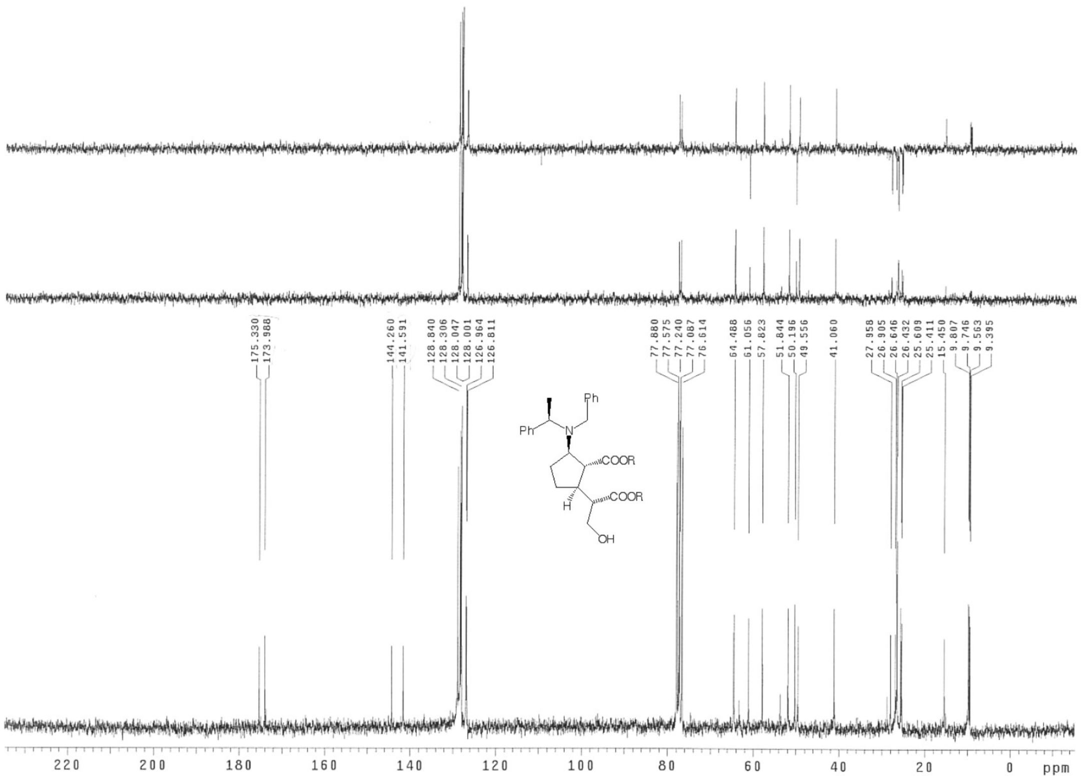
175.330
173.988
144.260
141.591
128.840
128.306
128.047
128.001
126.964
126.811
77.880
77.575
77.240
77.087
76.614
64.488
61.056
57.823
51.844
50.196
49.556
41.060
27.958
26.905
26.646
26.432
25.609
25.411
15.450
9.807
9.746
9.563
9.395
Ph
Ph
N
COOR
COOR
H
OH
220
200
180
160
140
120
100
80
60
40
20
0
ppm

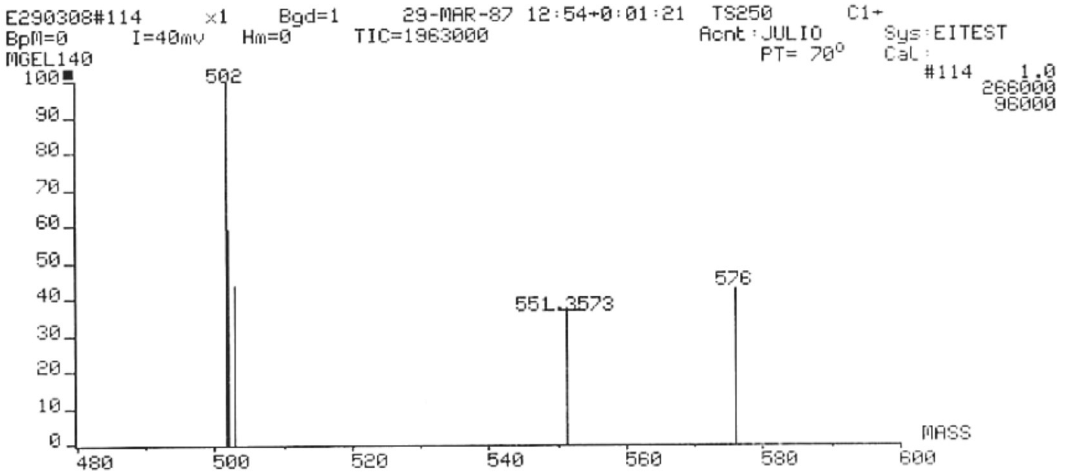
E290308#114 ×1 Bgd=1 29-MAR-87 12:54+0:01:21 TS250 C1+
BpM=0 I=40mv Hm=0 TIC=1963000 Acnt:JULIO Sys:EITEST
MGEL140 PT= 70° Cal:
#114 1.0
266000
96000
100
90
80
70
60
50
40
30
20
10
0
502
551.3573
576
480
500
520
540
560
580
600
MASS

13. ¹H RMN and ¹³C RMN for 15

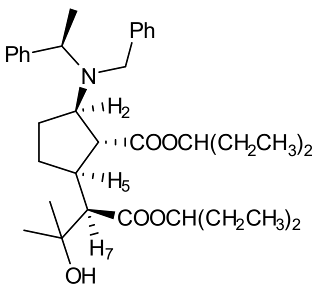

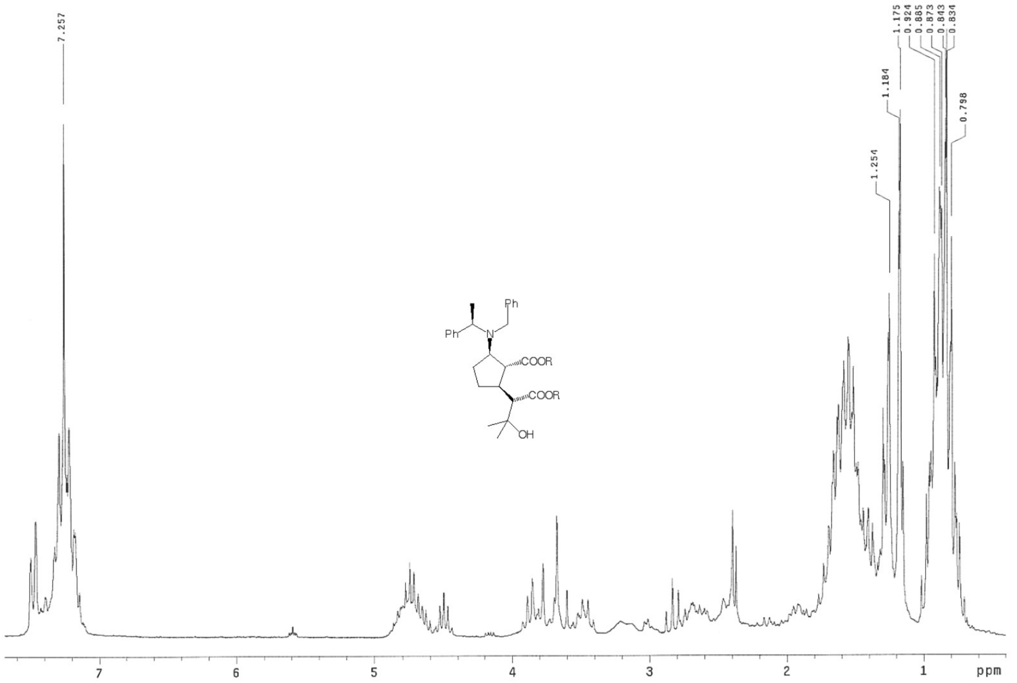

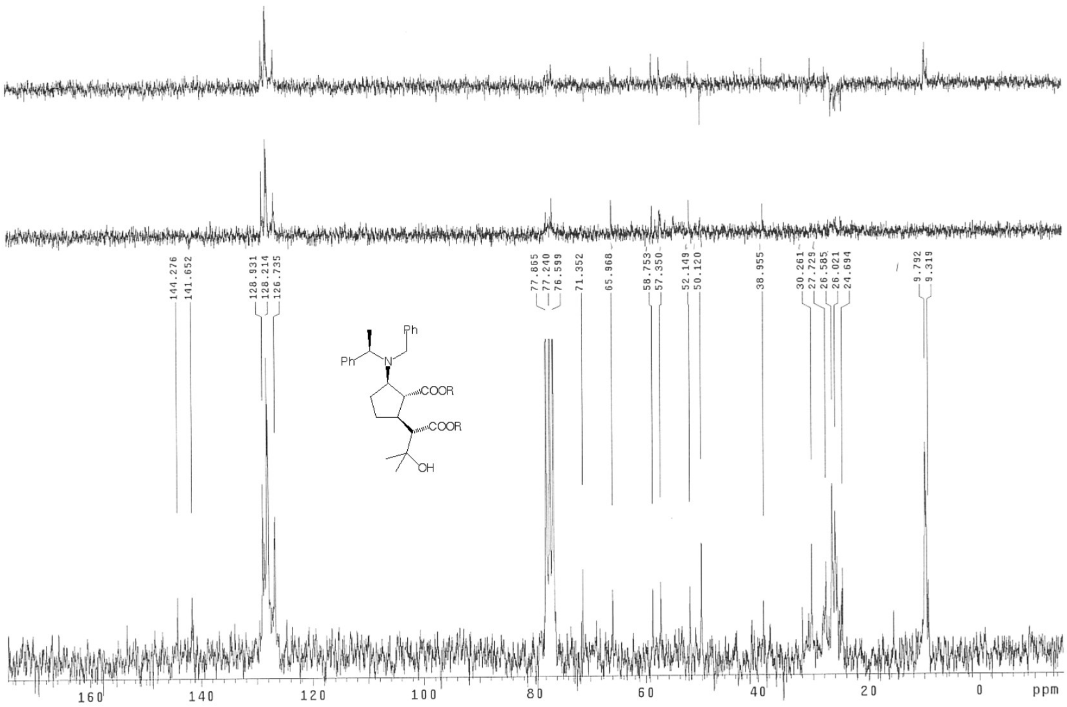

14. IR, ^{1}H RMN and ^{13}C RMN for 16

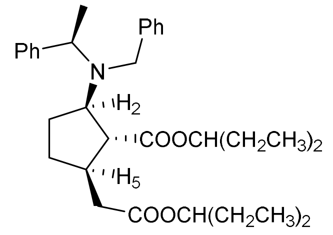

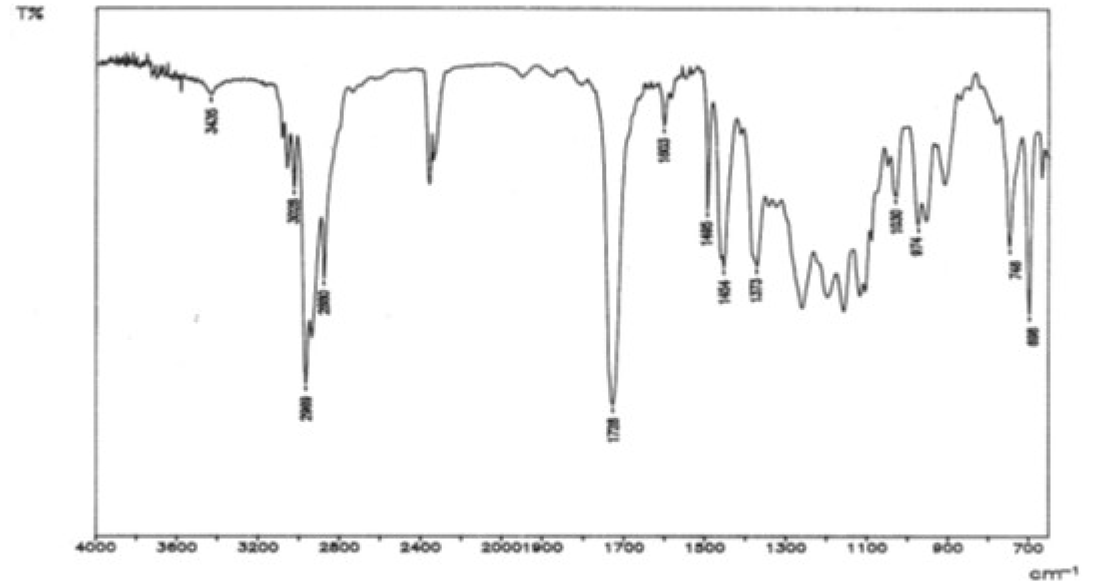

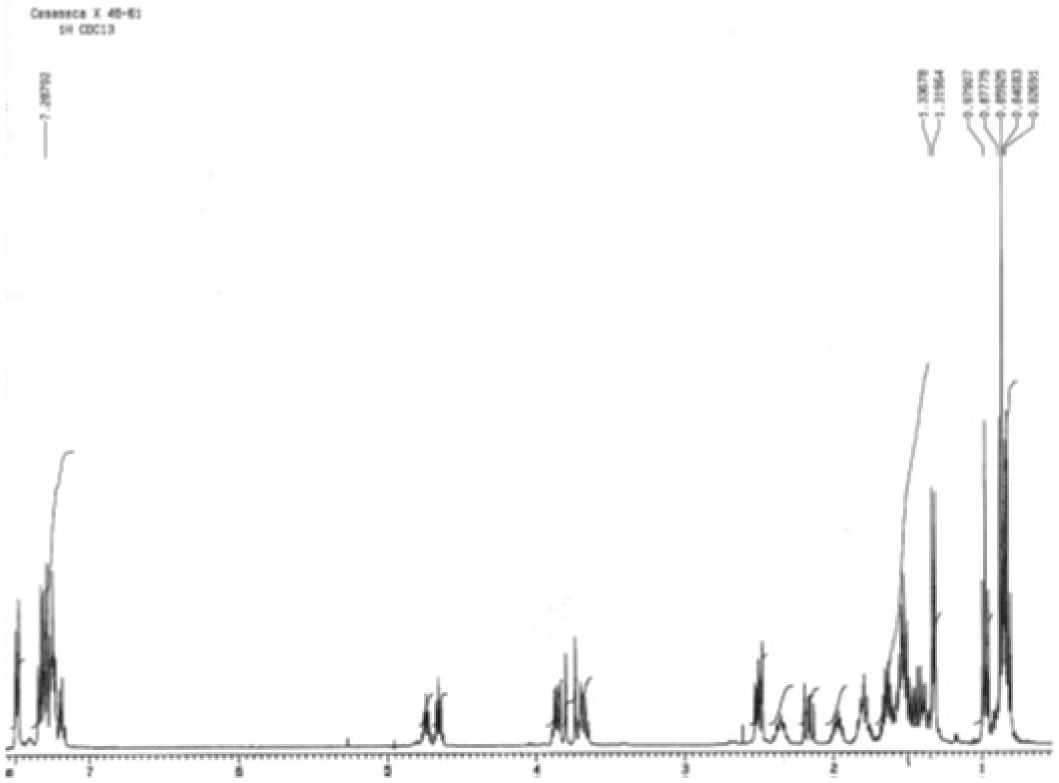

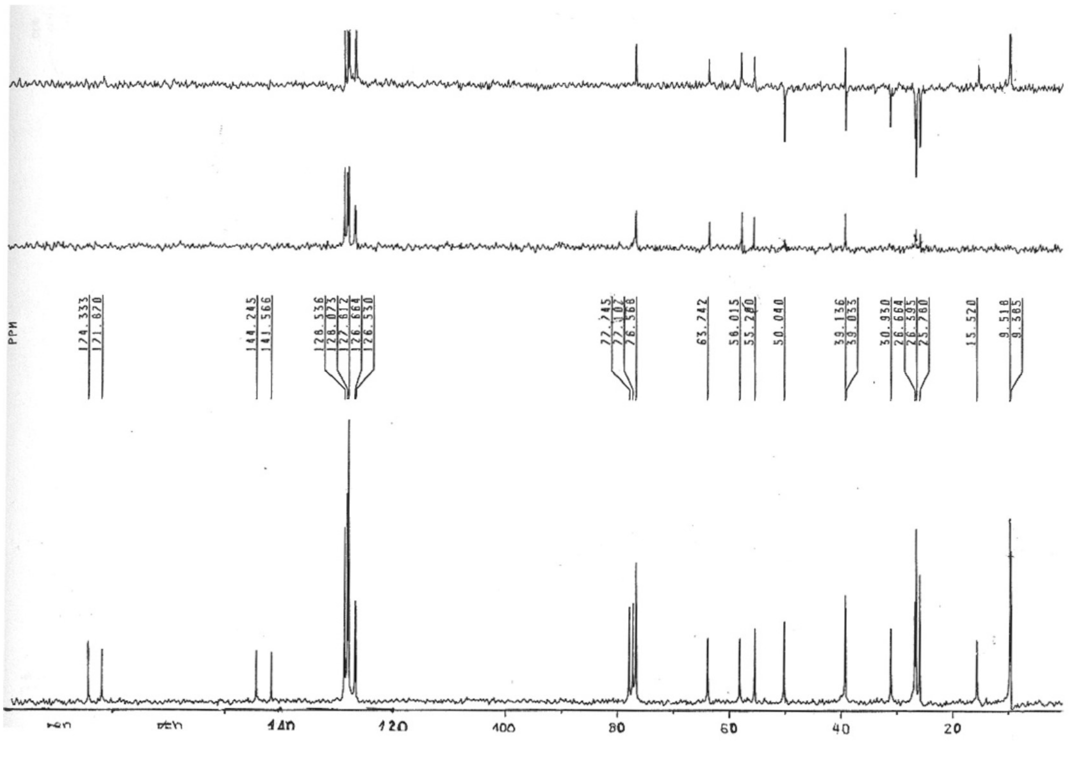

15. IR, ^{1}H RMN, ^{13}C RMN and HRMS for 17

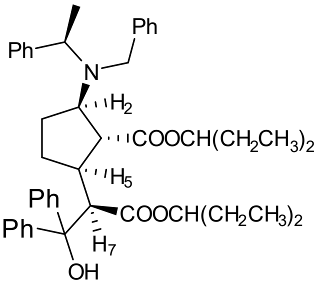

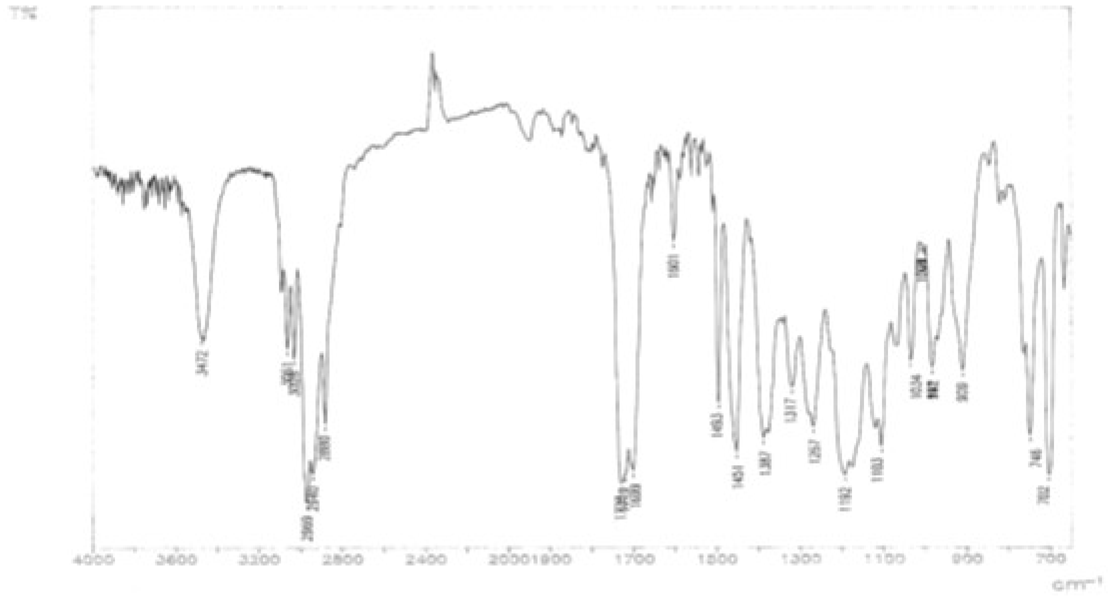

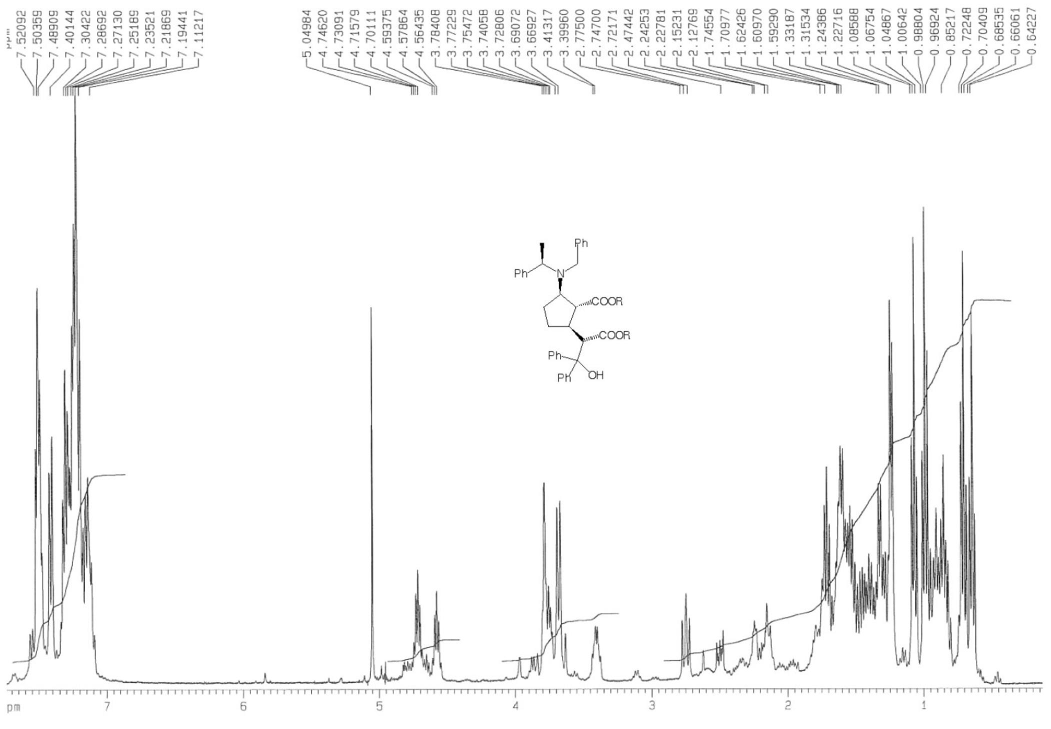

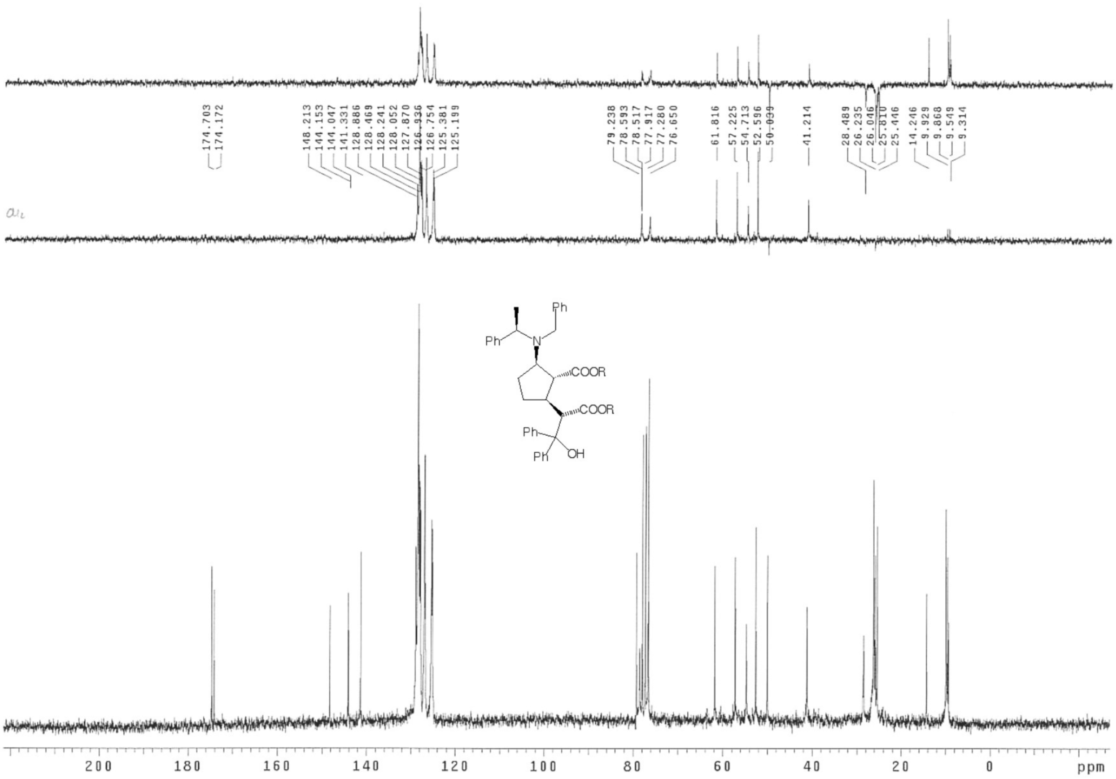
174.703
174.172
148.213
144.153
144.047
141.331
128.886
128.469
128.241
128.052
127.870
126.936
126.754
125.381
125.199
79.238
78.593
78.517
77.917
77.280
76.650
61.816
57.225
54.713
52.596
50.099
41.214
28.489
26.235
26.046
25.810
25.446
14.246
9.929
9.868
9.549
9.314
Ph
Ph
N
COOR
COOR
Ph
Ph
OH
200
180
160
140
120
100
80
60
40
20
0
ppm

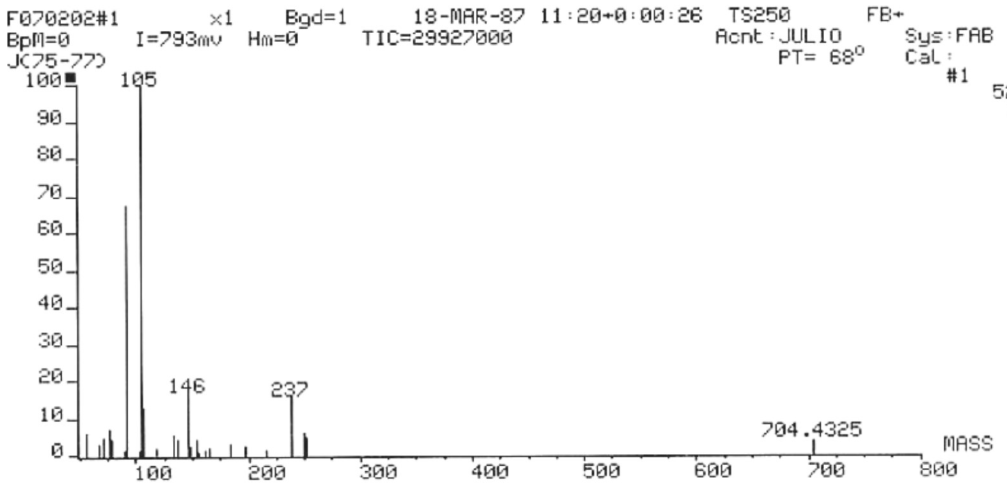
F070202#1 ×1 Bgd=1 18-MAR-87 11:20+0:00:26 TS250 FB+
BpM=0 I=793mv Hm=0 TIC=29927000 Acnt:JULIO Sys:FAB
J(75-77) PT= 68° Cal:
#1 1.0
5200000
191000
105
146
237
704.4325
MASS
100
200
300
400
500
600
700
800

16. IR and ^{1}H RMN for 18

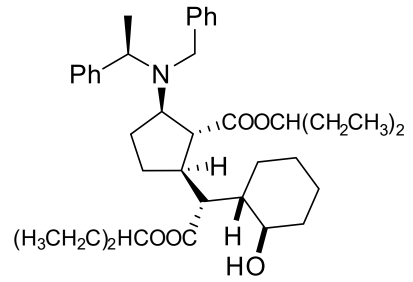

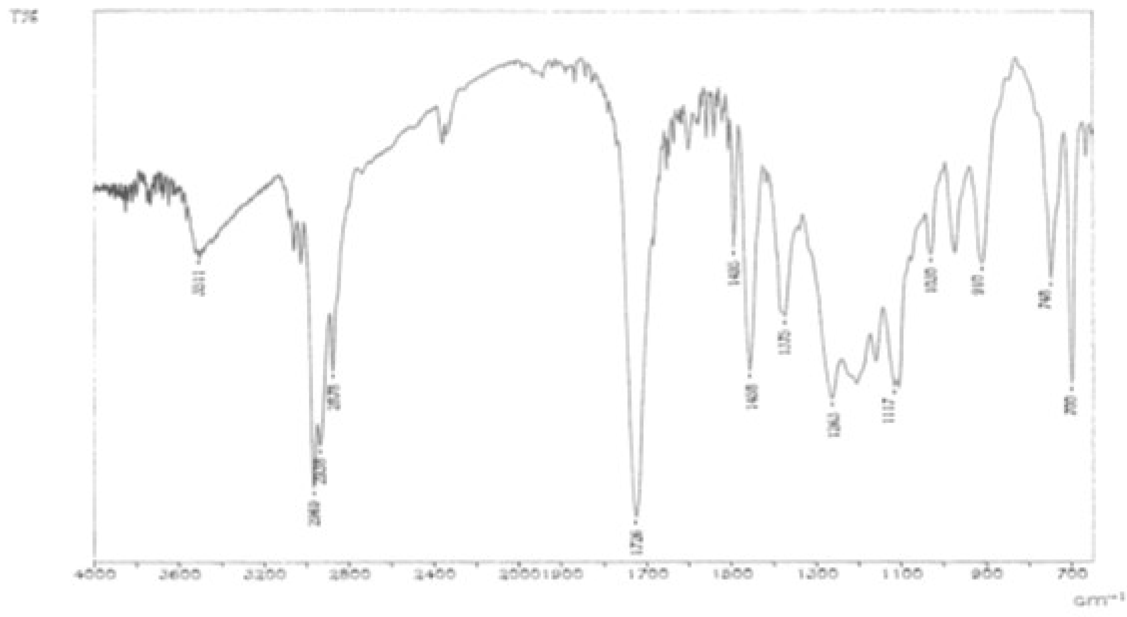

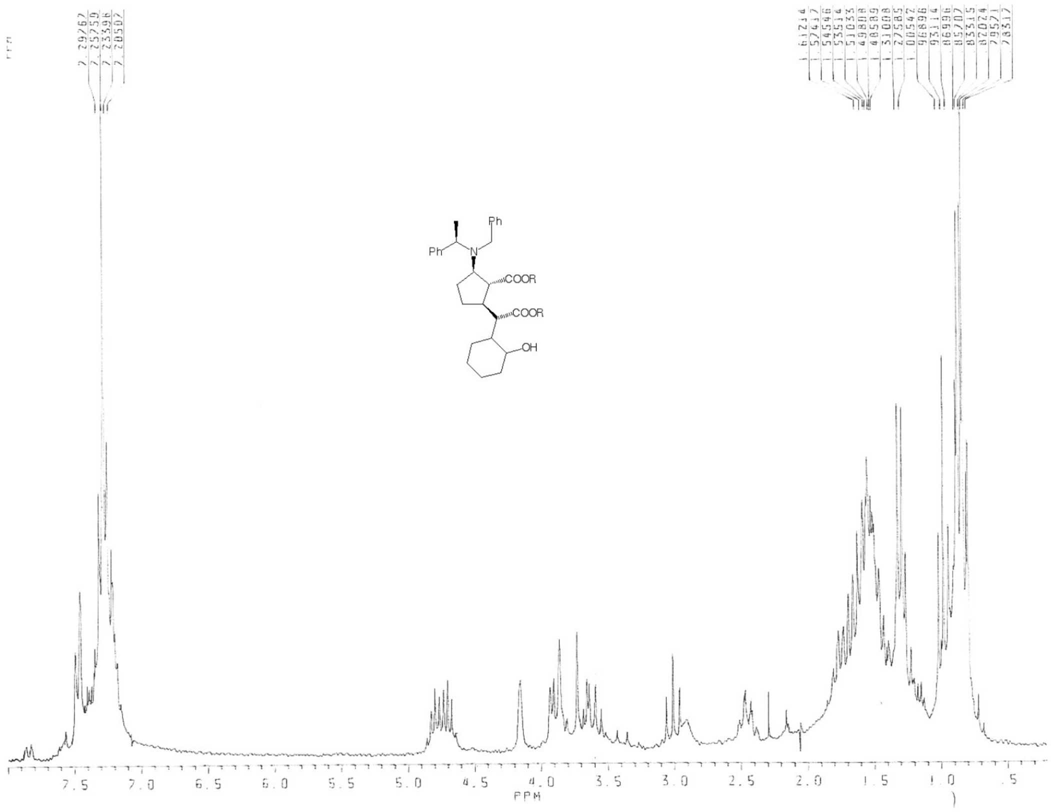

17. IR, ^{1}H RMN, ^{13}C RMN and HRMS for 19

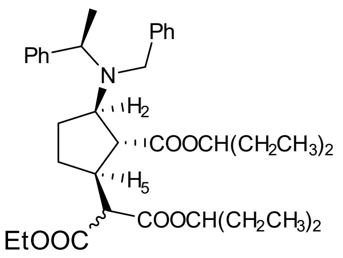

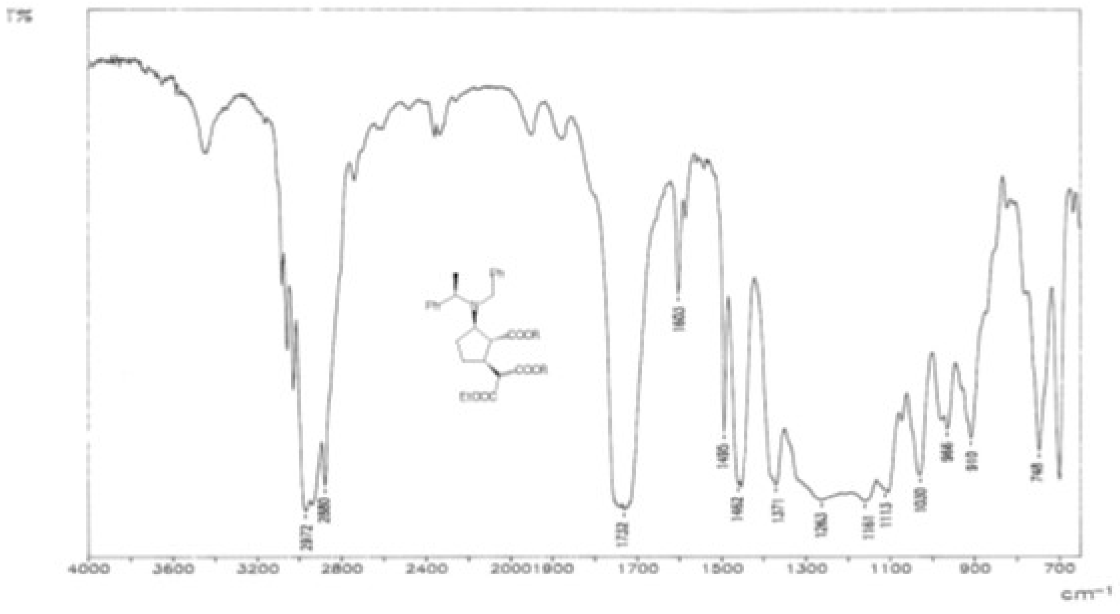

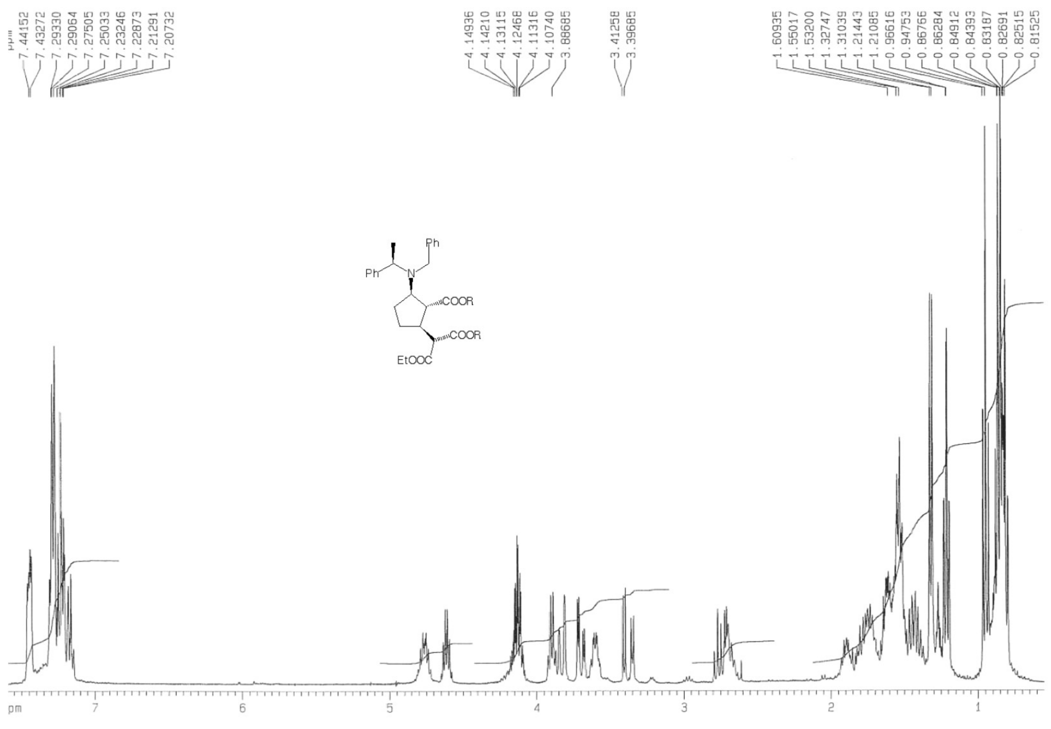

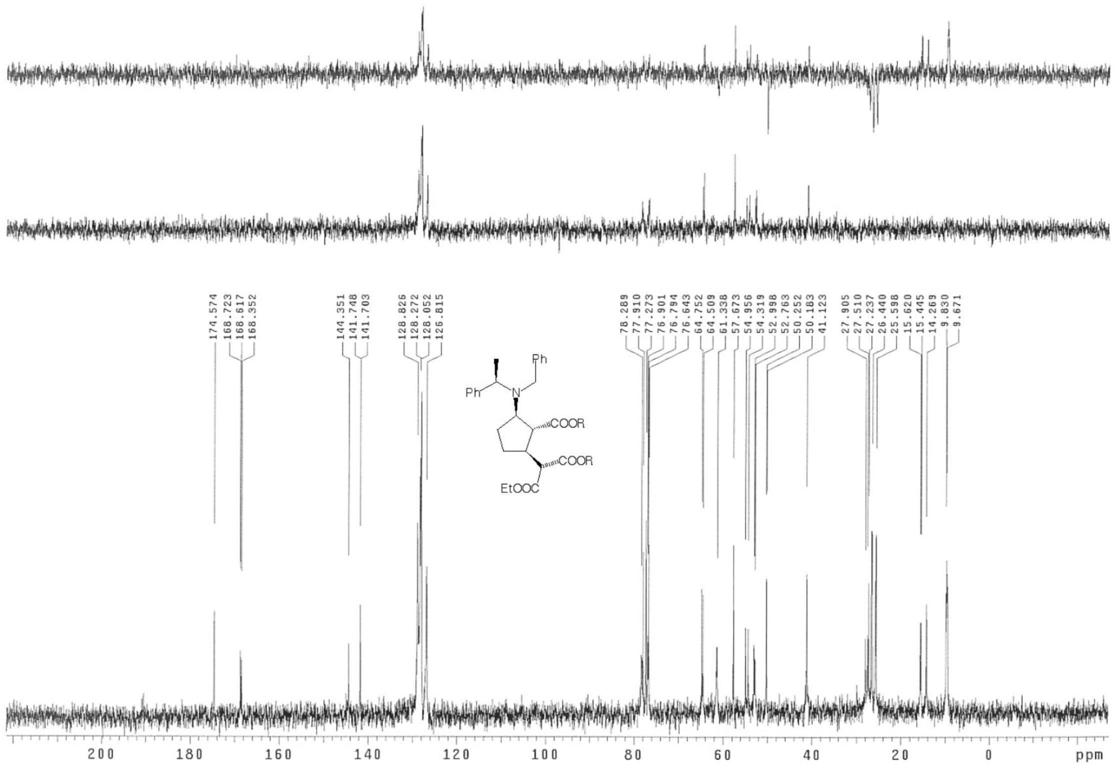
174.574 168.723 168.617 168.352
144.351 141.748 141.703
128.826 128.272 128.052 126.815
78.289 77.910 77.273 76.901 76.794 76.643 64.752 64.509 61.338 57.673 54.956 54.319 52.998 52.763 50.252 50.183 41.123
27.905 27.510 27.237 26.440 25.598 15.620 15.445 14.269 9.830 9.671
Ph
Ph
N
COOR
COOR
EtOOC
200 180 160 140 120 100 80 60 40 20 0 ppm

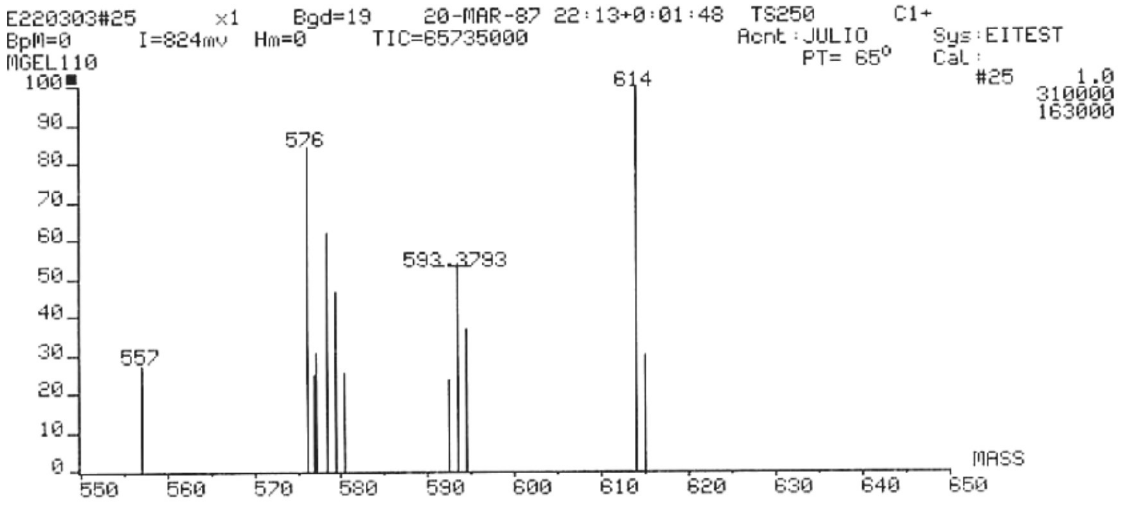
E220303#25 ×1 Bgd=19 20-MAR-87 22:13+0:01:48 TS250 C1+
BpM=0 I=824mv Hm=0 TIC=65735000 Acnt:JULIO Sys:EITEST
MGEL110 PT= 65° Cal:
#25 1.0
310000
163000
614
576
593.3793
557
MASS

18. IR, ^{1}H RMN and ^{13}C RMN for 20

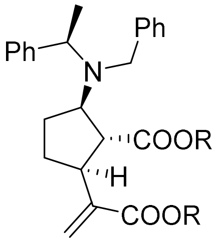

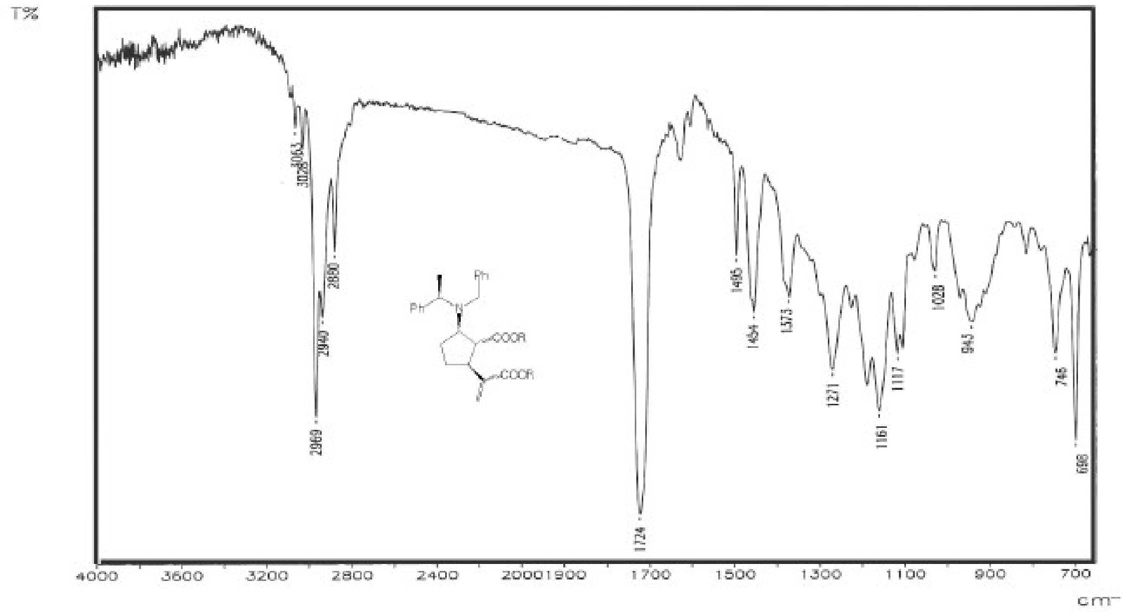

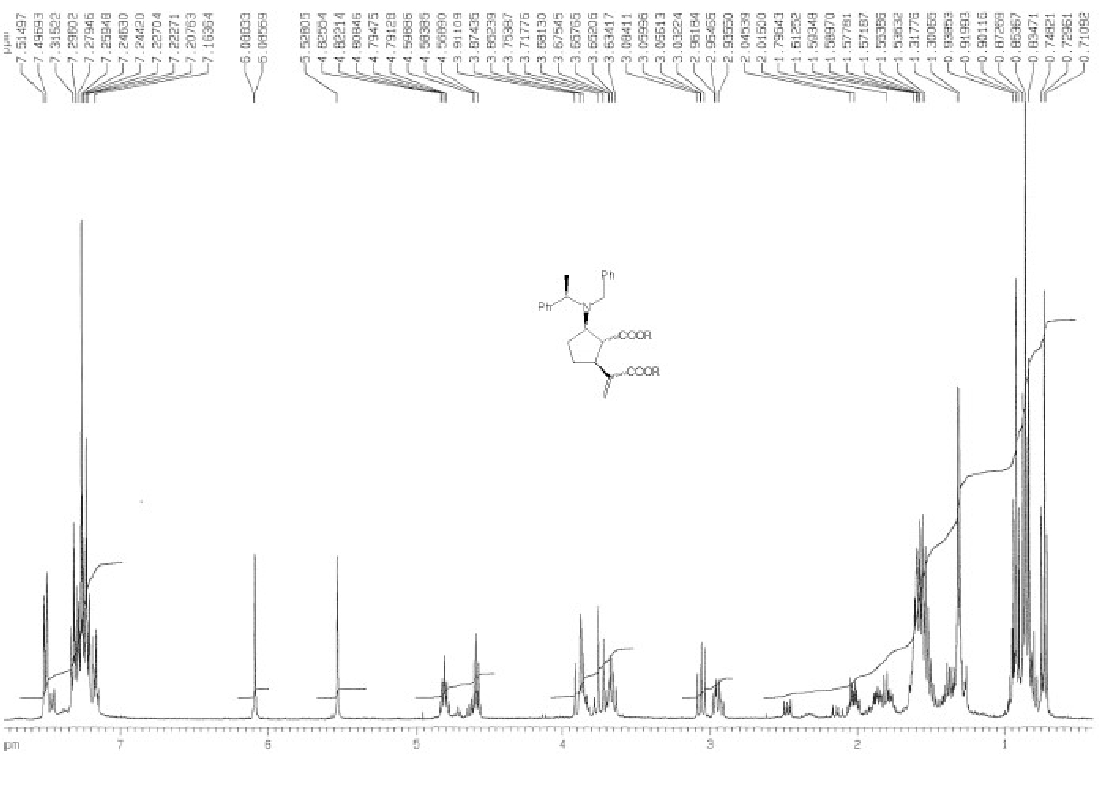

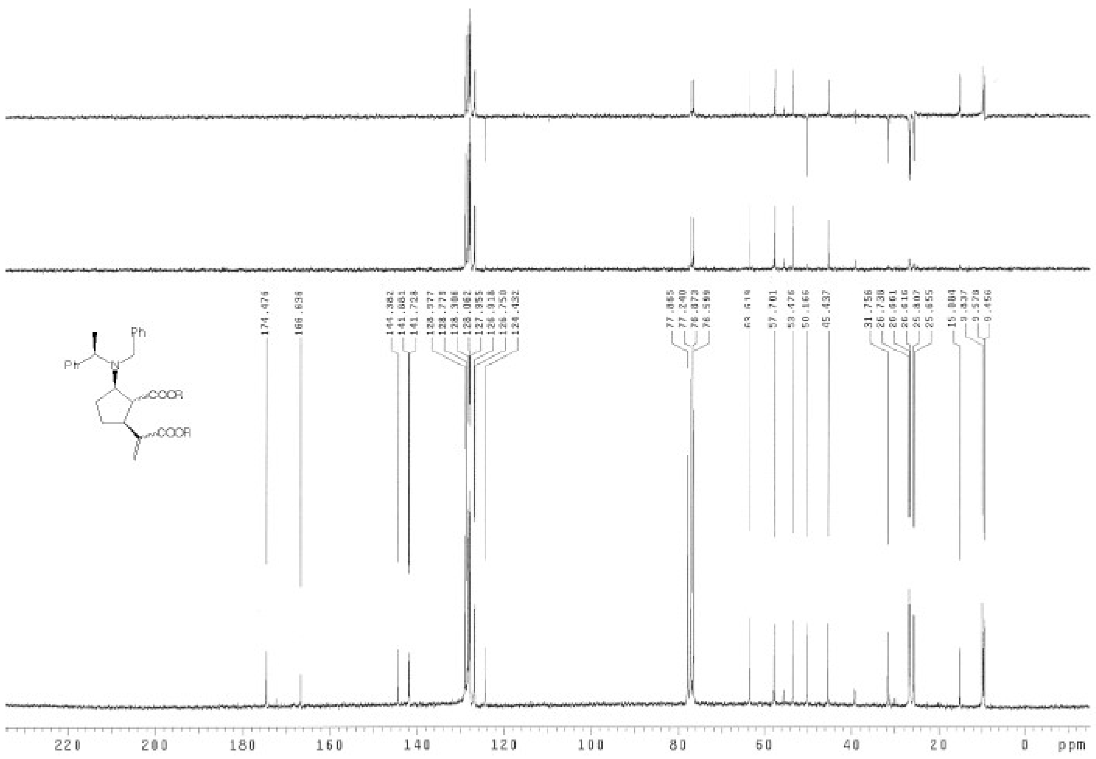

19. ^{1}H RMN, ^{13}C RMN, ROESY, COSY and HRMS for 21

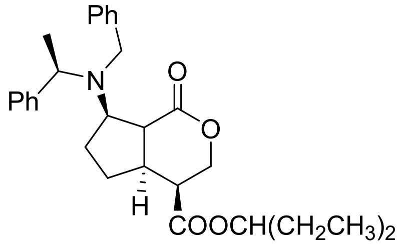

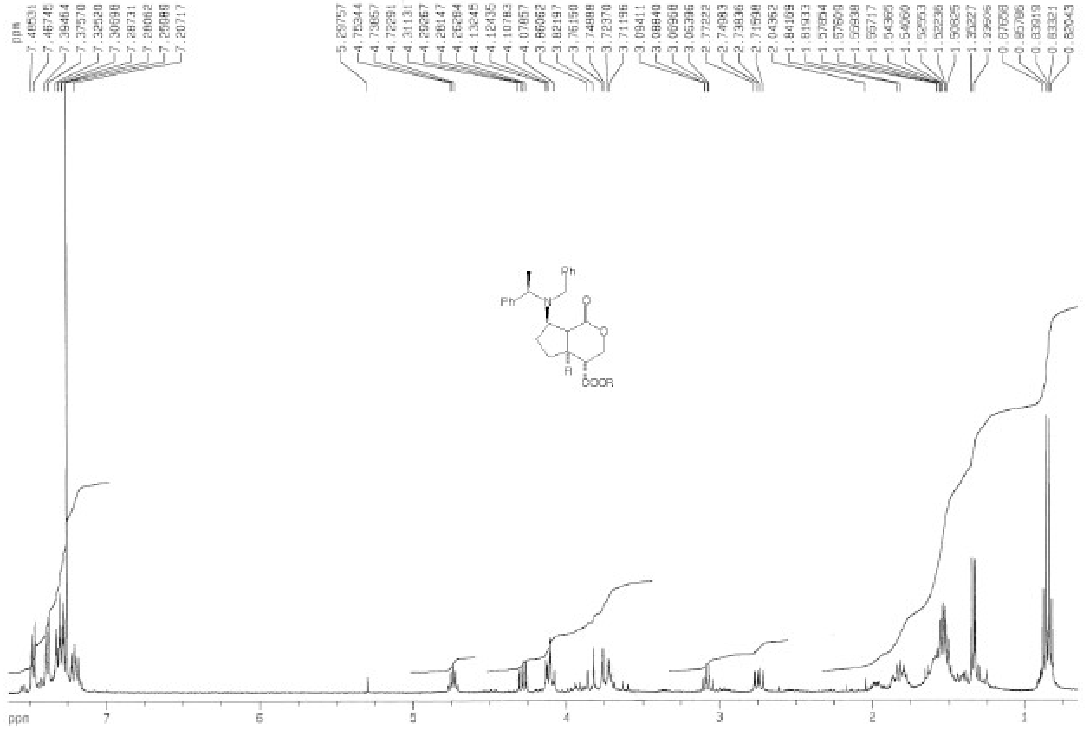

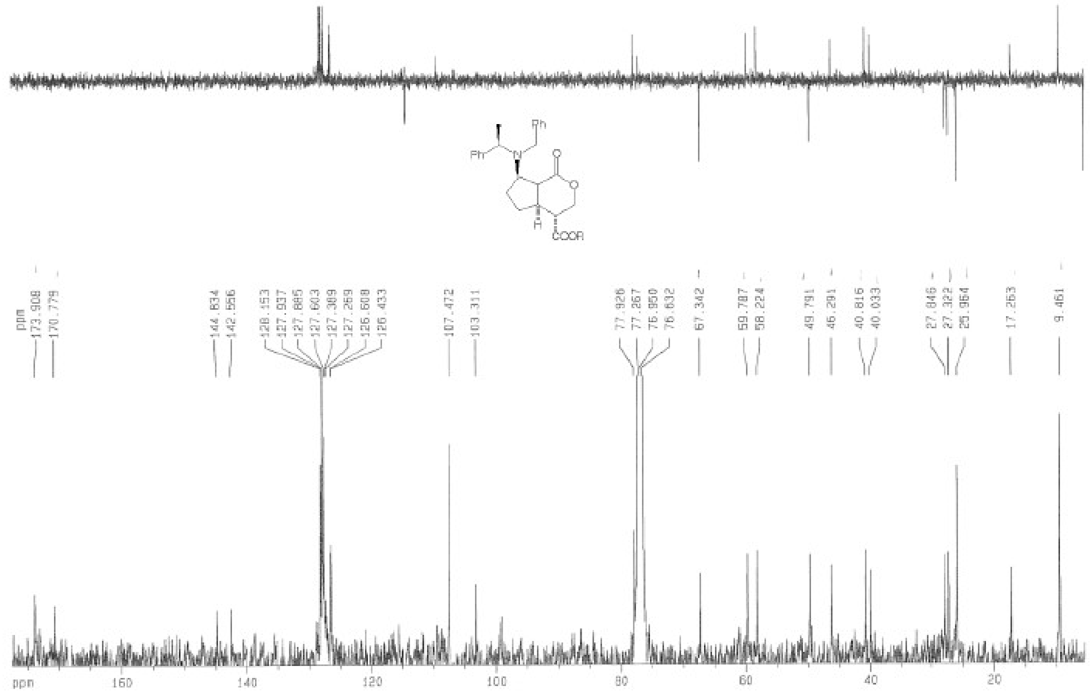

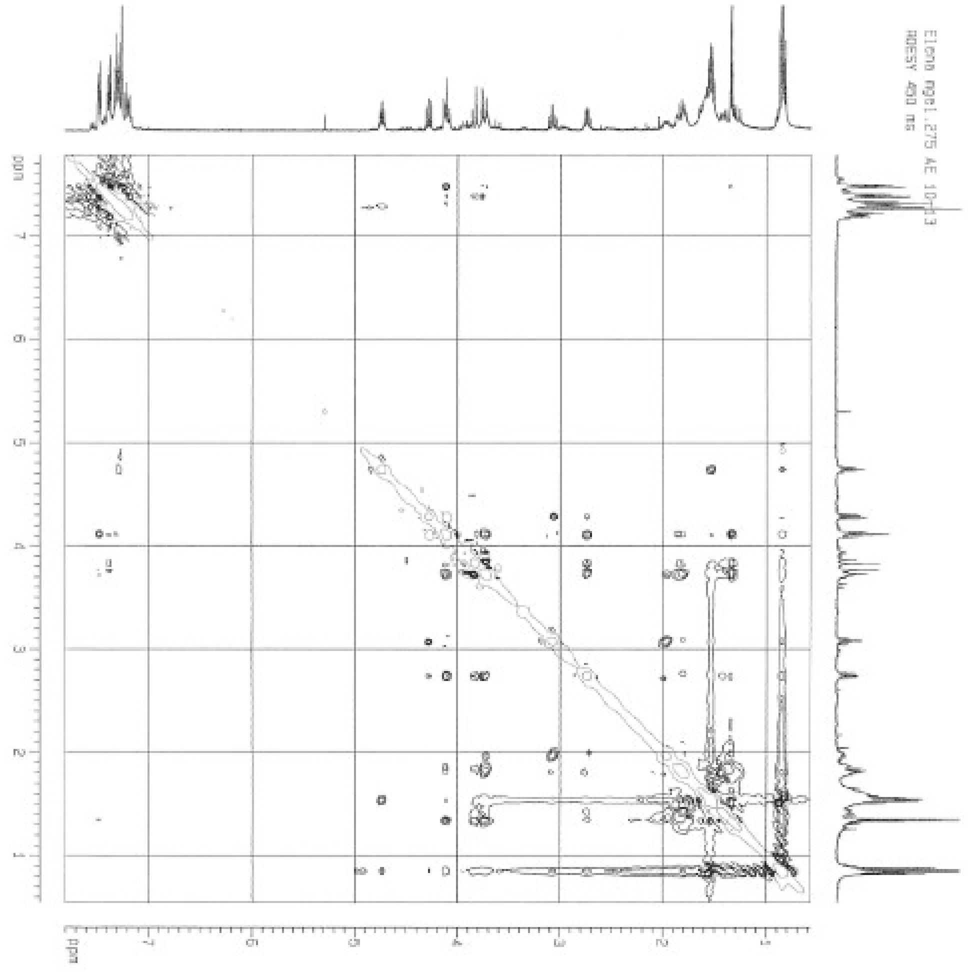
Elena agel.275 AE 10-13
ROESY 400 ms

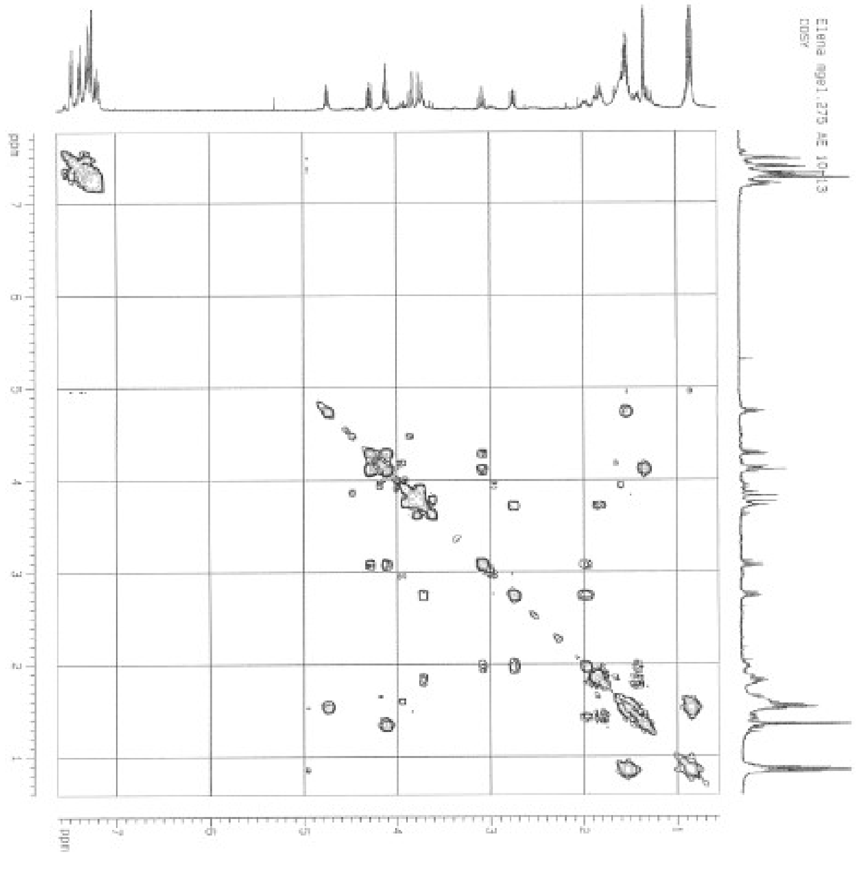
Elena agel.275 AE 10-13
COSY

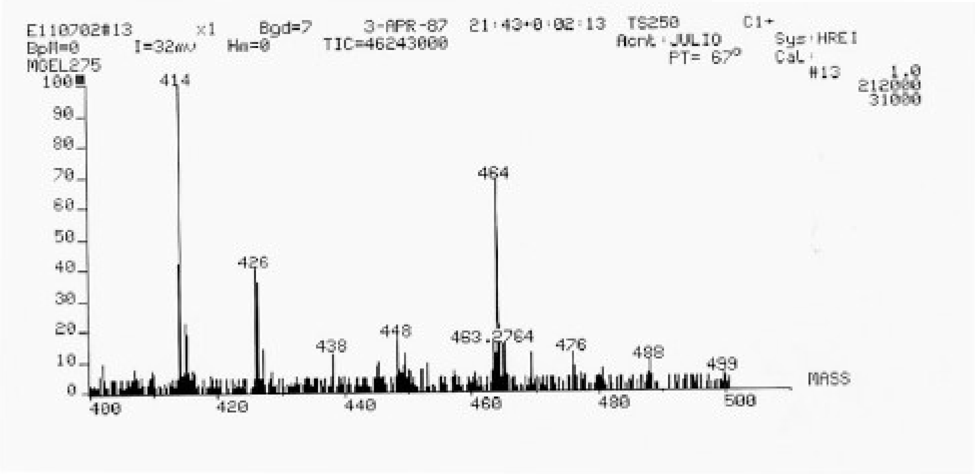
E110702#13 x1 Bgd=7 3-APR-87 21:43+0:02:13 TS250 CI+
BpM=0 I=32mv Hm=0 TIC=46243000
Acnt:JULIO Sys:HREI
PT= 67°
Cal:
MGEL275
#13 1.0
212000
31000
414
464
426
438
448
463.2764
476
488
499
MASS
0
10
20
30
40
50
60
70
80
90
100
400
420
440
460
480
500

20. IR, ^{1}H RMN, ^{13}C RMN, COSY and HRMS for 22

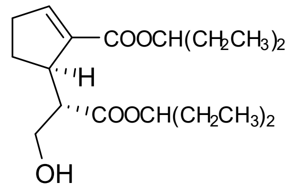

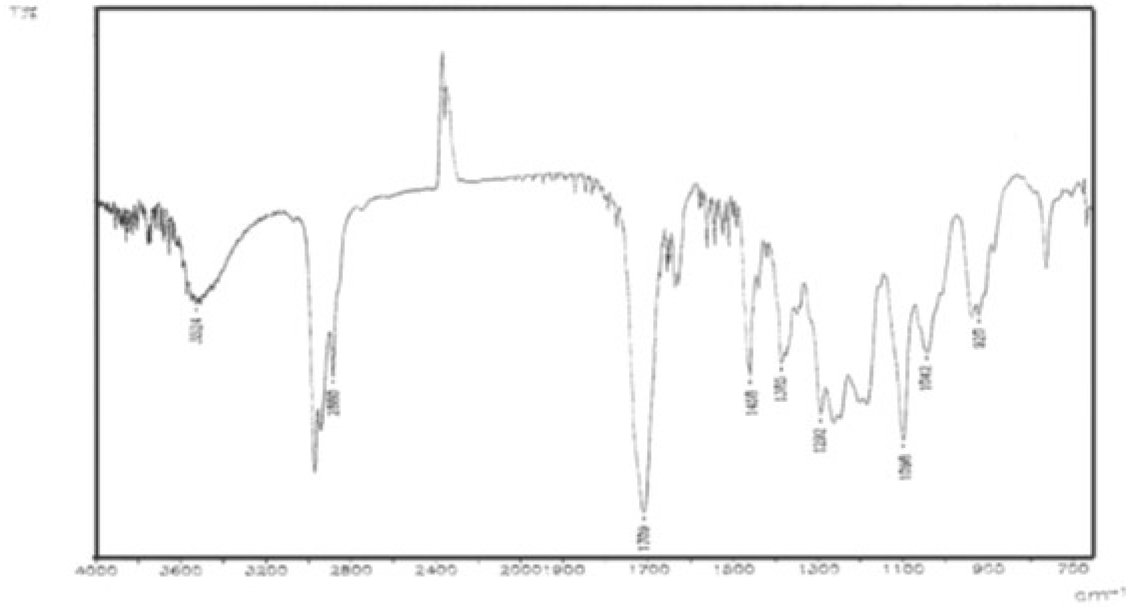

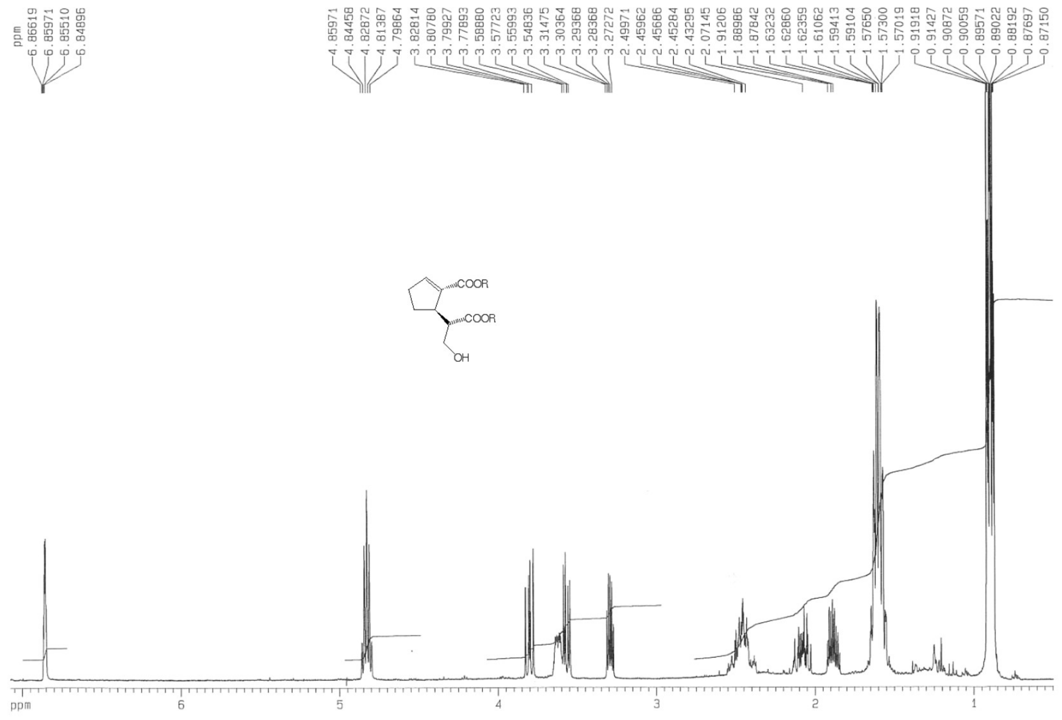

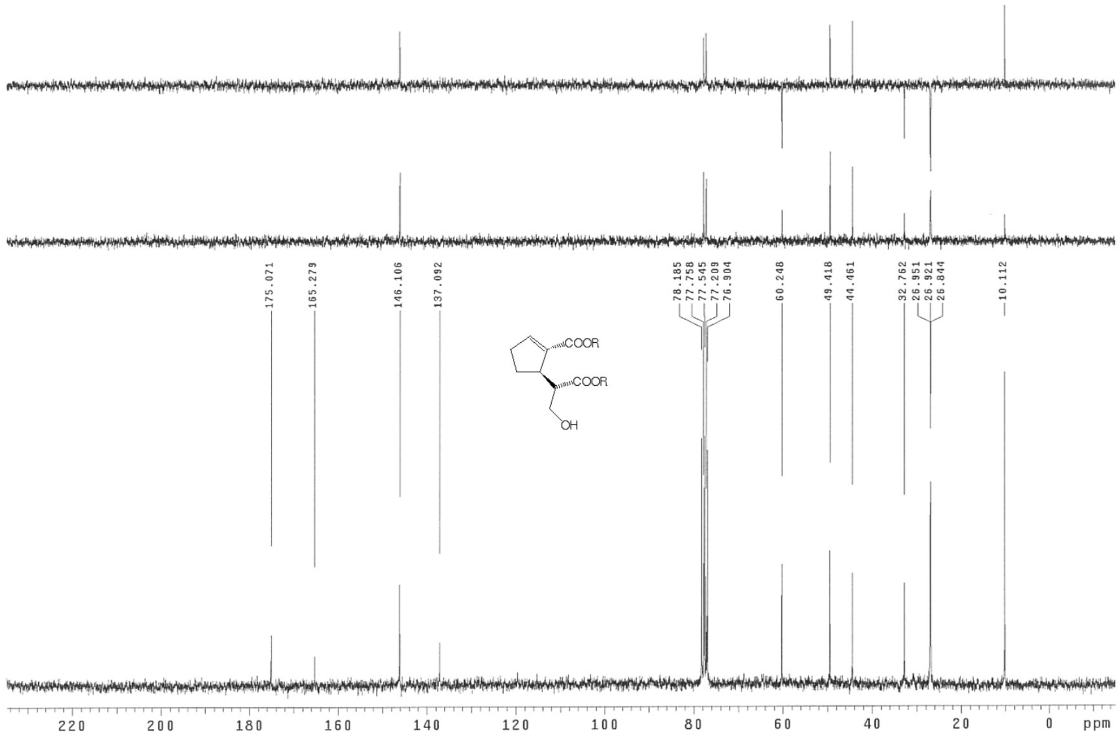
175.071
165.279
146.106
137.092
78.185
77.758
77.545
77.203
76.304
60.248
49.418
44.461
32.762
26.951
26.921
26.844
10.112
COOR
COOR
OH
220 200 180 160 140 120 100 80 60 40 20 0 ppm

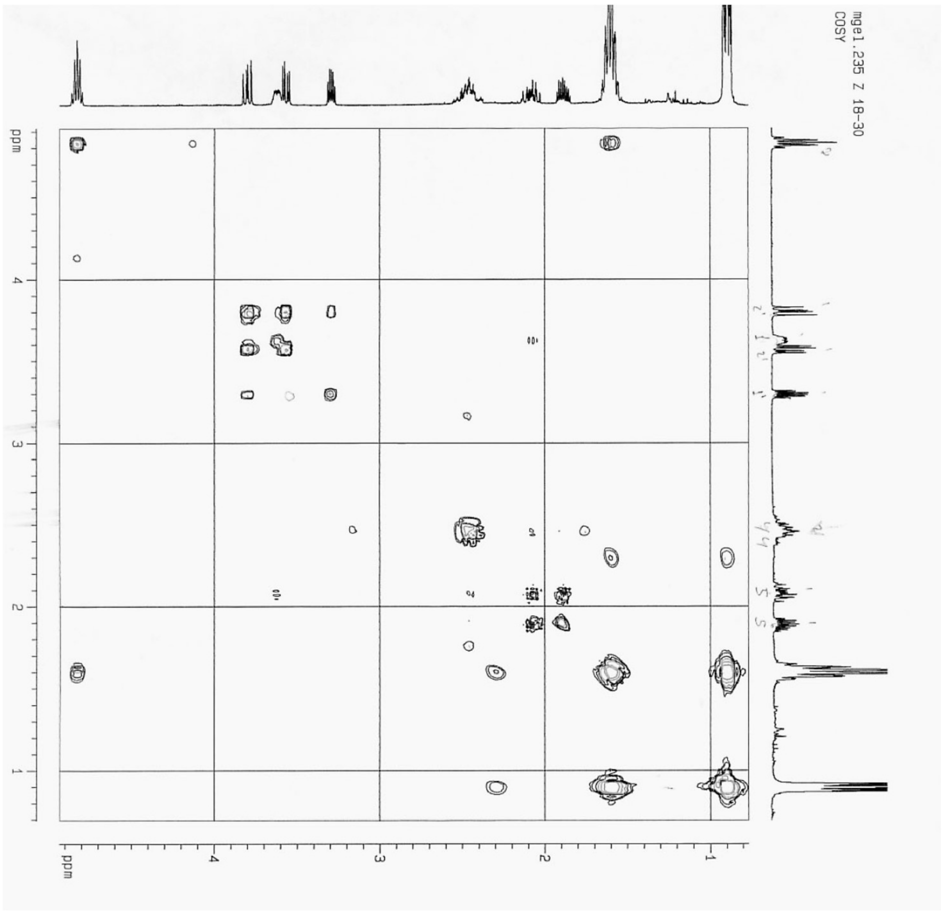
mge1.235 Z 18-30
COSY
ppm

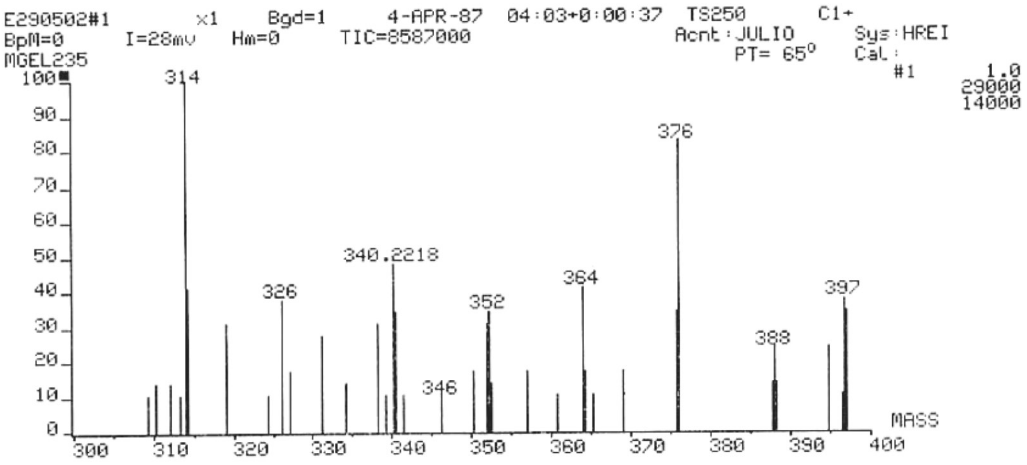
E290502#1 ×1 Bgd=1 4-APR-87 04:03+0:00:37 TS250 C1+
BpM=0 I=28mv Hm=0 TIC=8587000
Acnt:JULIO Sys:HREI
PT= 65° Cal:
MGEL235
#1 1.0
29000
14000
314
376
340.2218
326
352
364
397
388
346
MASS

21. IR, ^{1}H RMN, ^{13}C RMN and HRMS for 23

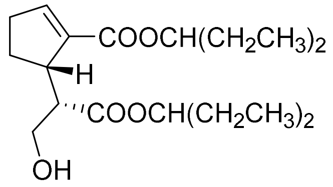

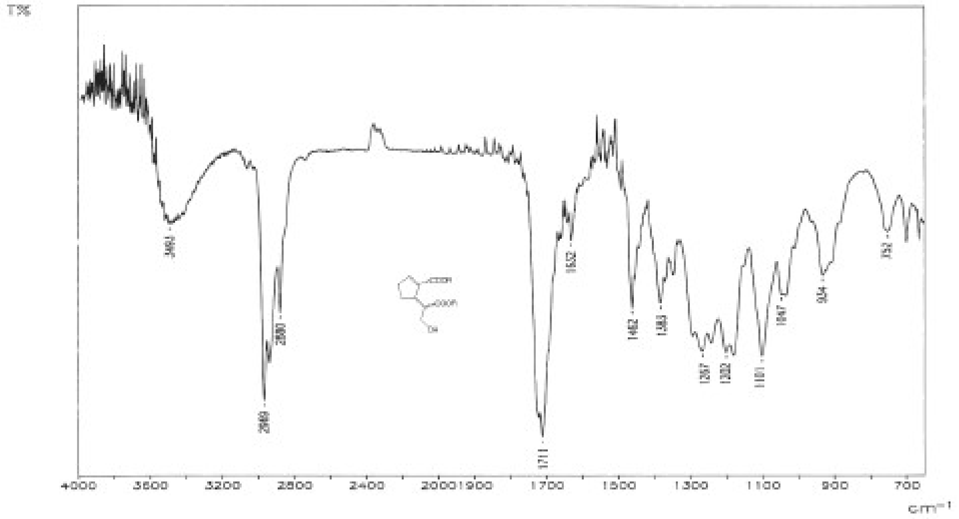

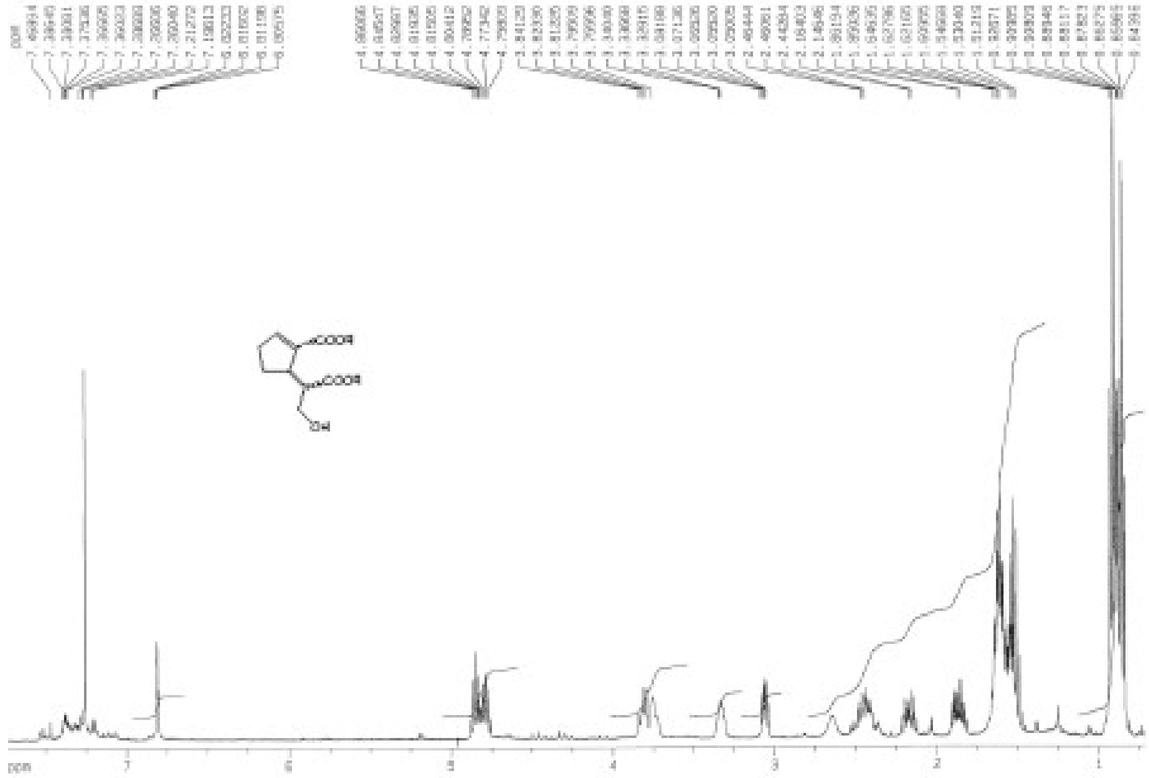

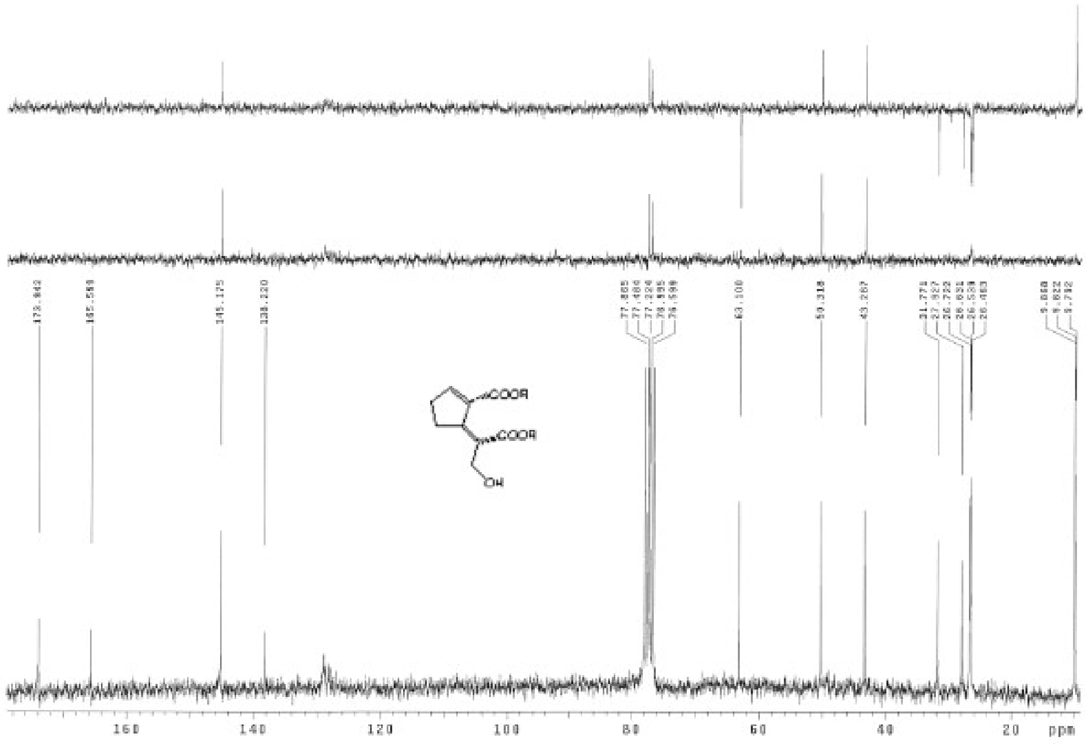

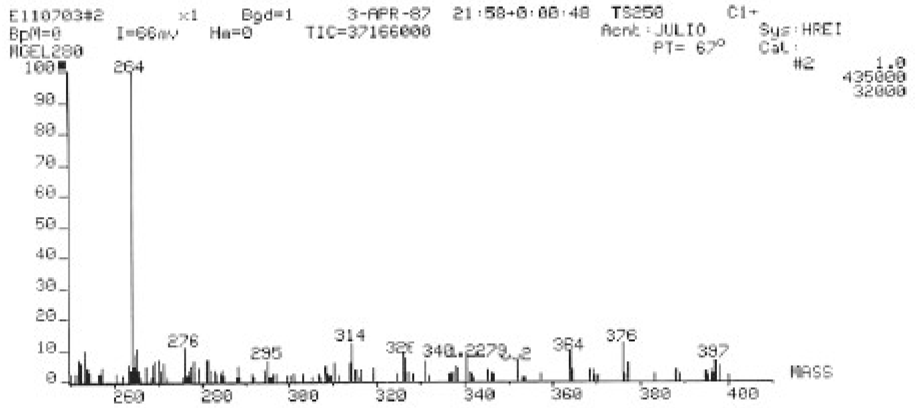

22. ¹H RMN, ¹³C RMN and COSY for 24

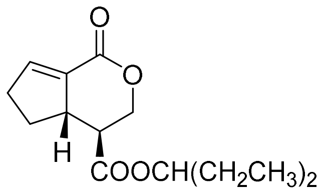

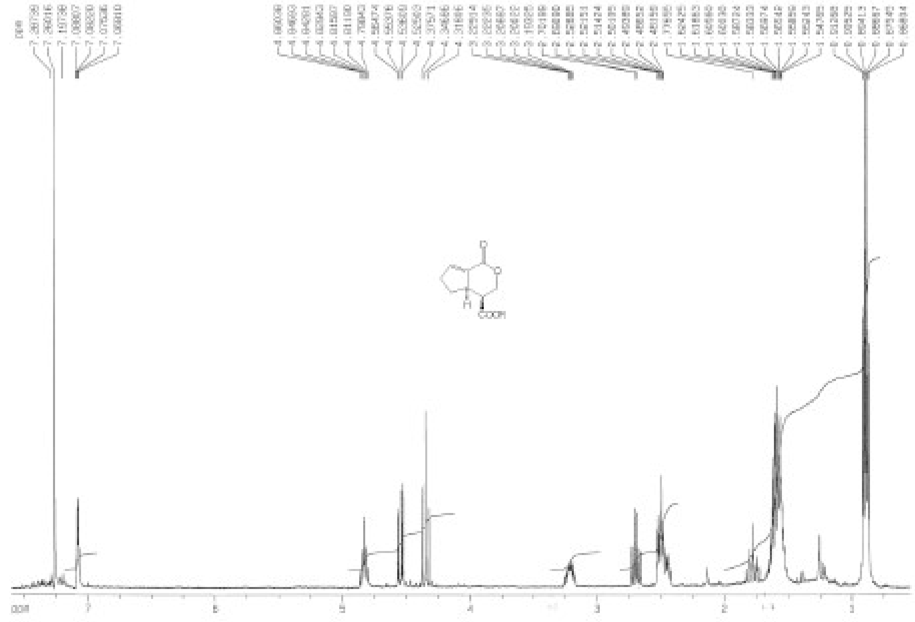

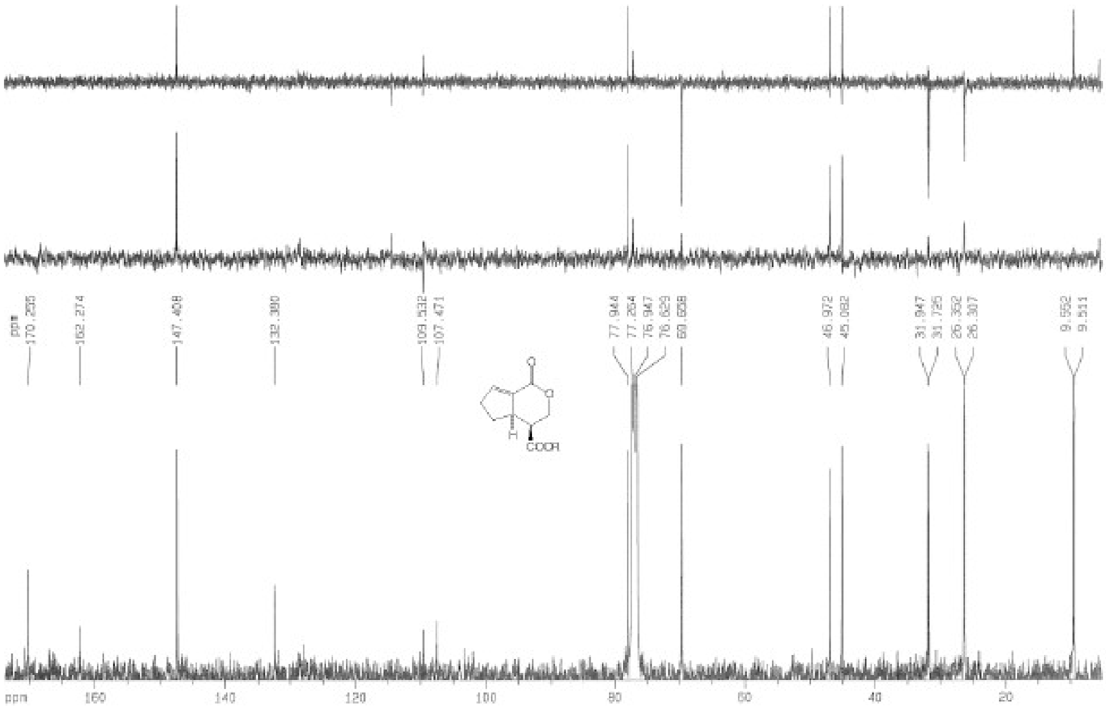

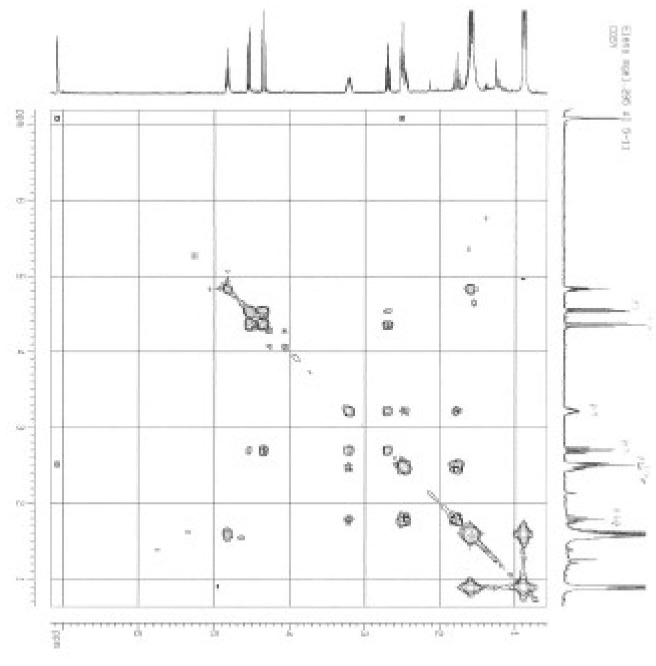

23. IR, ^{1}H RMN, ^{13}C RMN, COSY and HRMS for 25

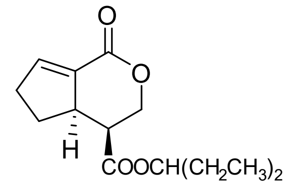

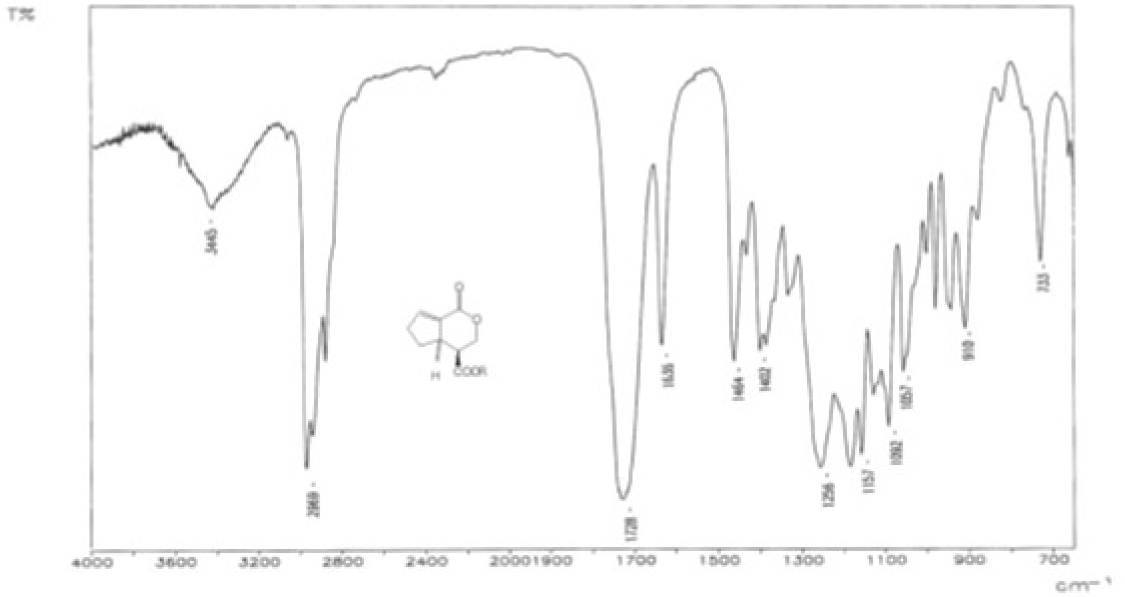

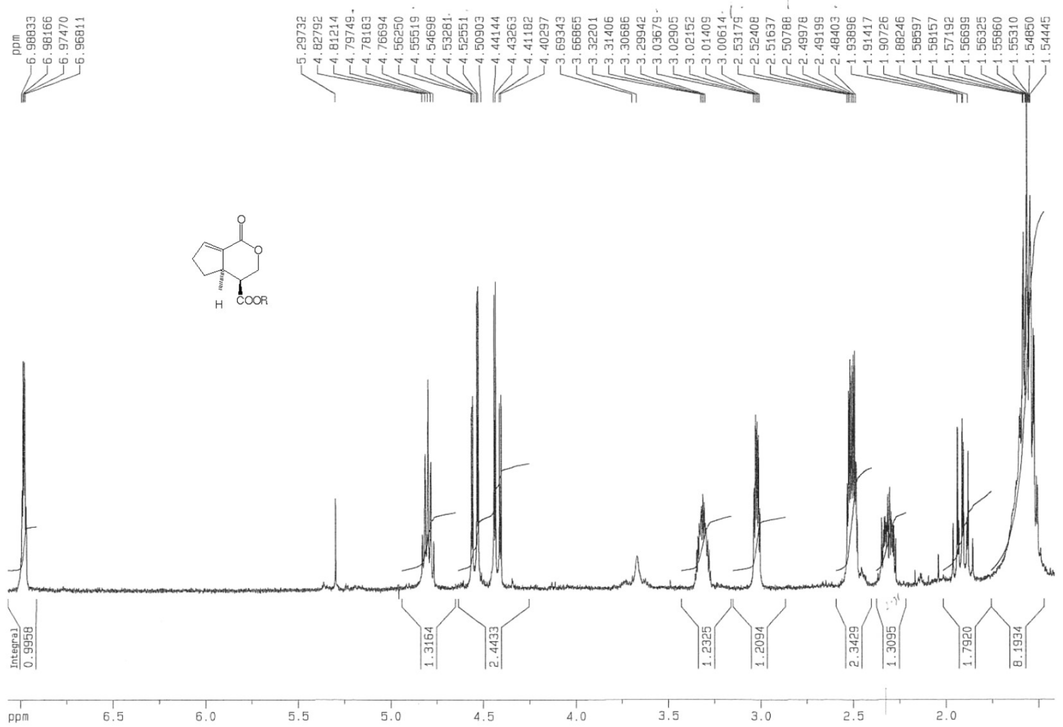

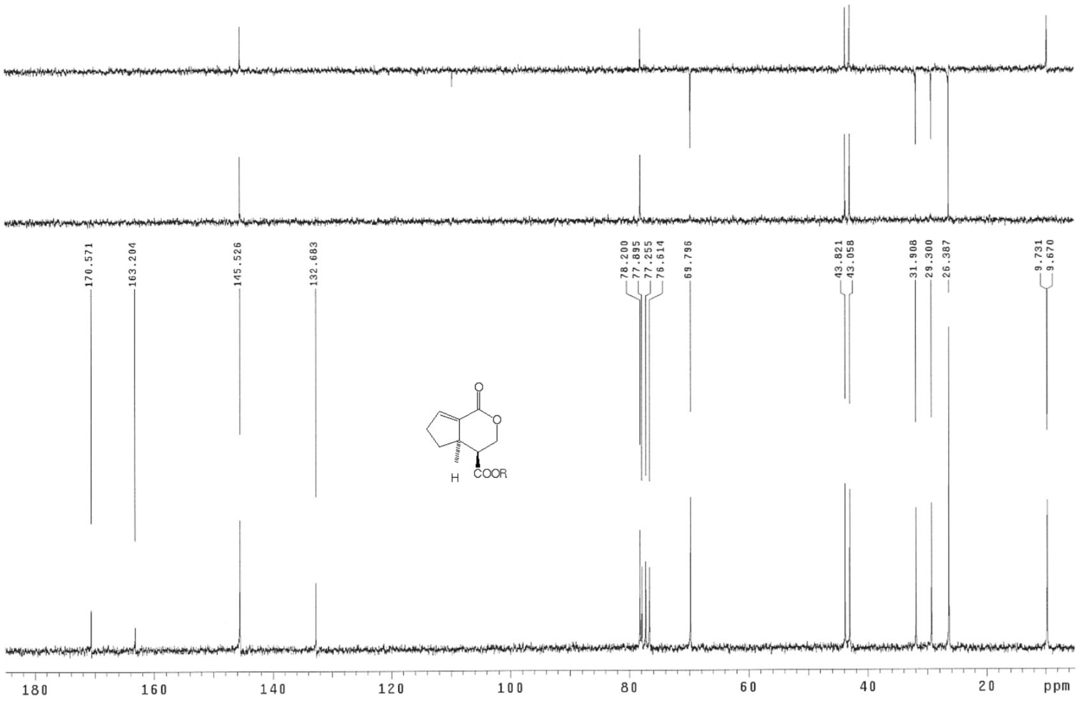
170.571
163.204
145.526
132.683
78.200
77.895
77.255
76.614
69.796
43.821
43.058
31.908
29.300
26.387
9.731
9.670
O
O
H
COOR
180
160
140
120
100
80
60
40
20
ppm

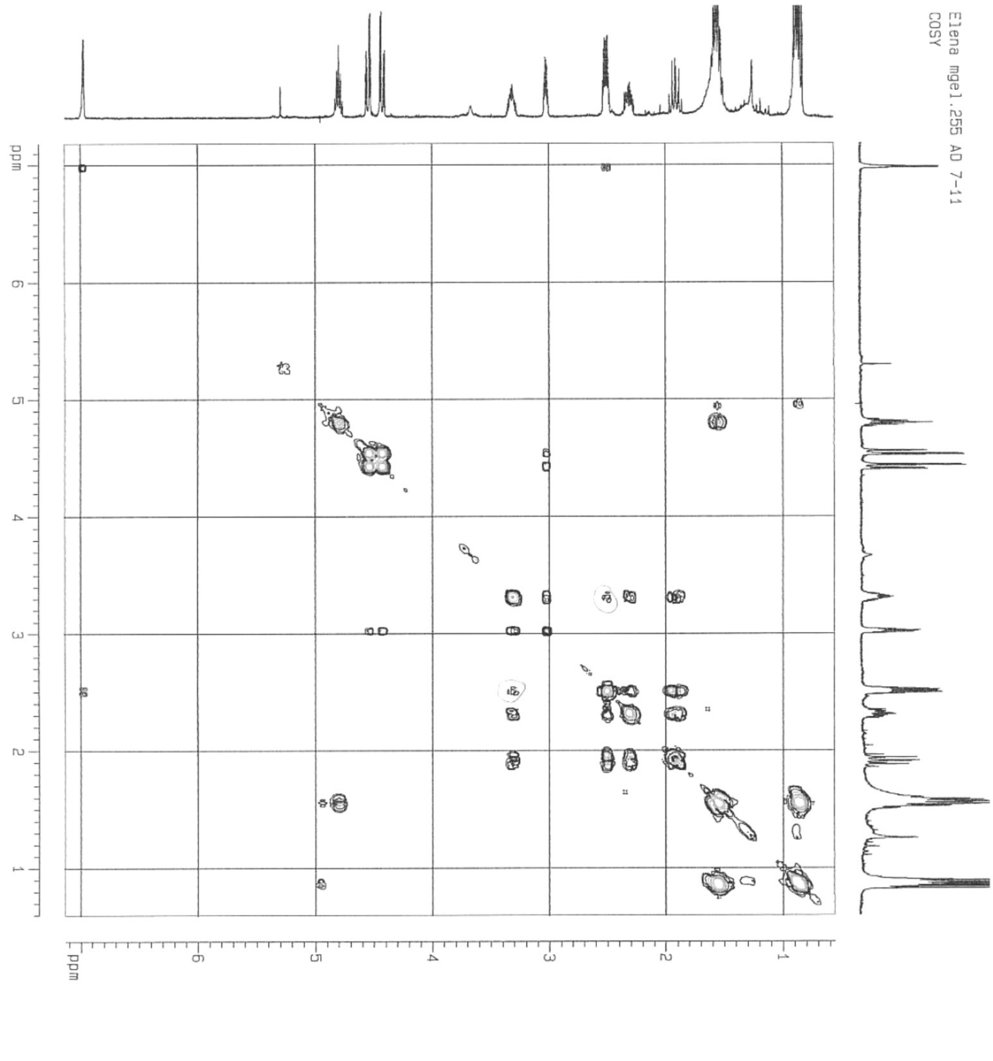
Elena mgel.255 AD 7-11
COSY
ppm
6
5
4
3
2
1

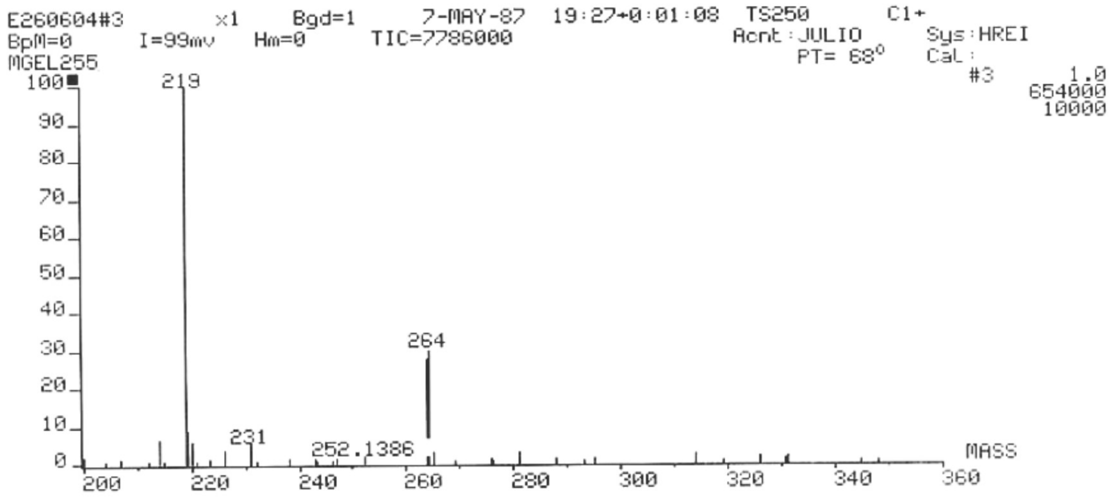
E260604#3 ×1 Bgd=1 7-MAY-87 19:27+0:01:08 TS250 C1+
BpM=0 I=99mv Hm=0 TIC=7786000 Acnt:JULIO Sys:HREI
MGEL255 PT= 68° Cal:
#3 1.0
654000
10000
219
264
231
252.1386
MASS

24. IR, ^{1}H RMN, ^{13}C RMN, COSY and HRMS for 26

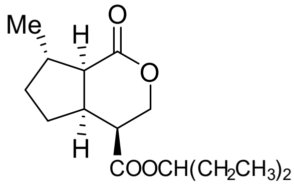

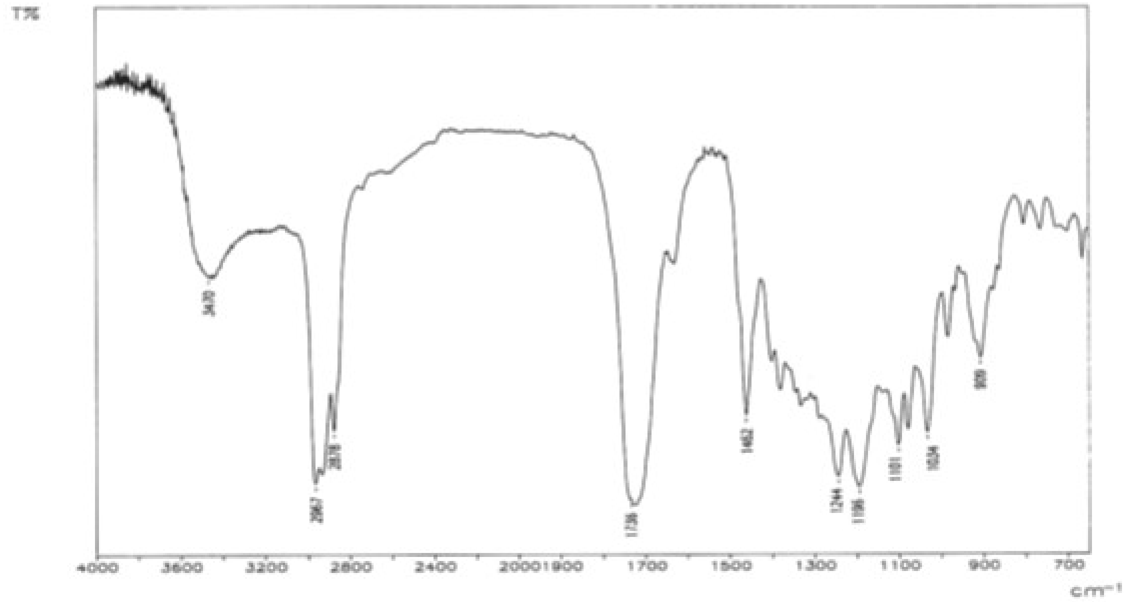

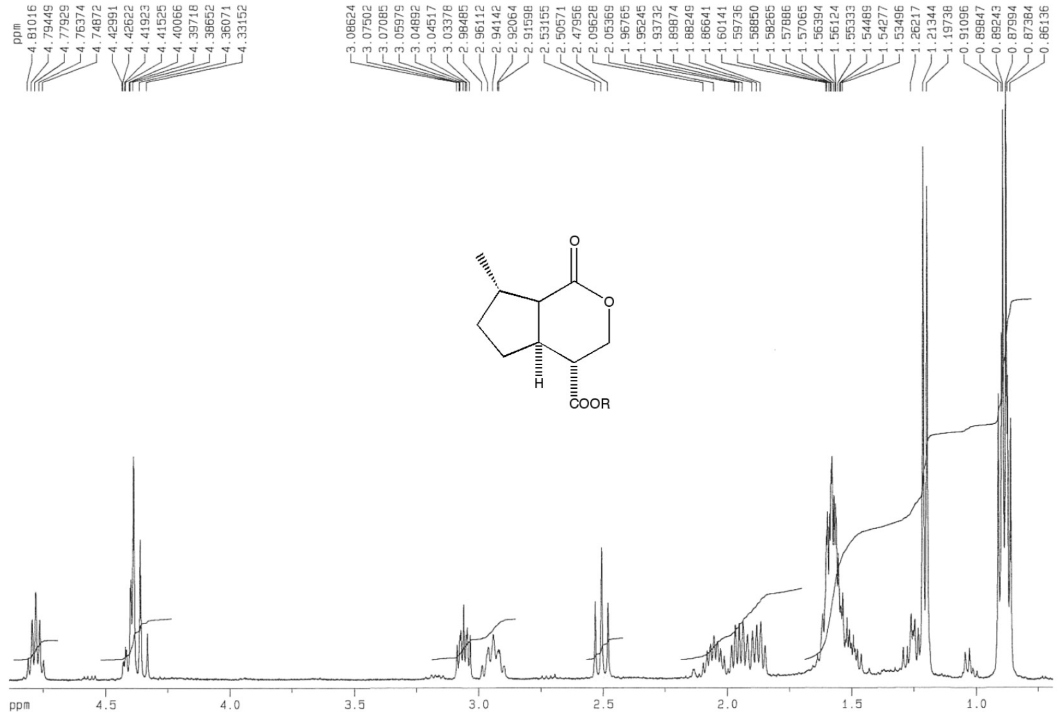

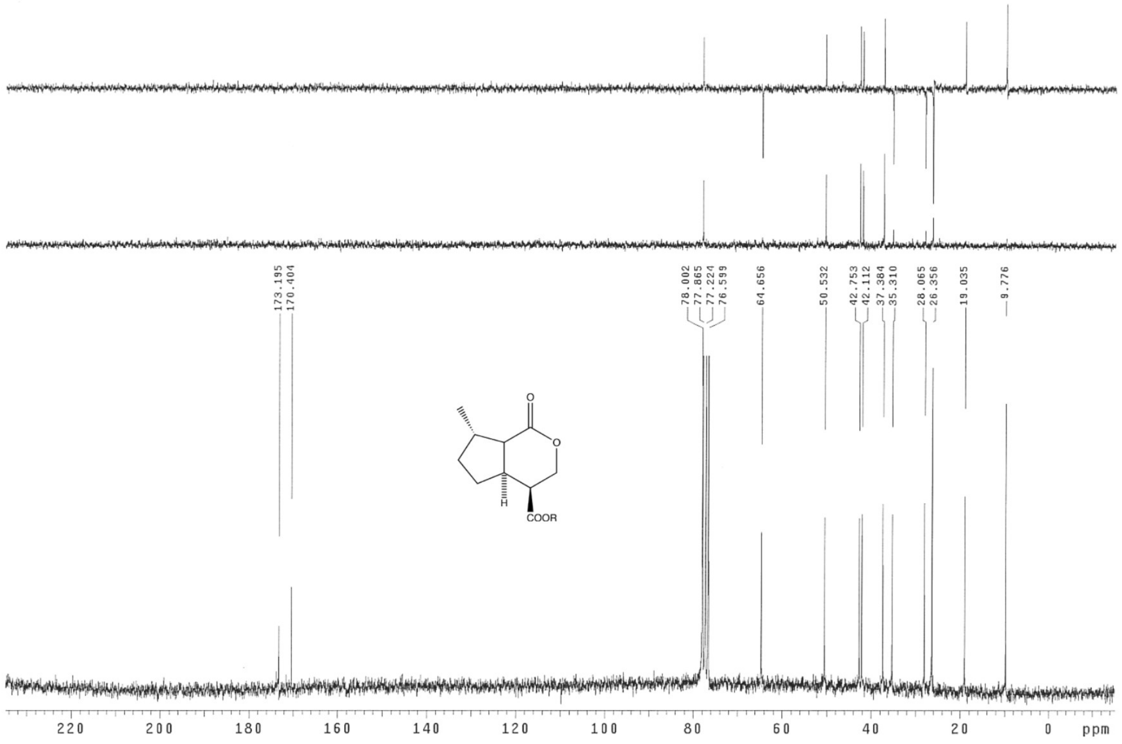
173.195
170.404
78.002
77.865
77.224
76.599
64.656
50.532
42.753
42.112
37.384
35.310
28.065
26.356
19.035
9.776
COOR
H
O
220 200 180 160 140 120 100 80 60 40 20 0 ppm

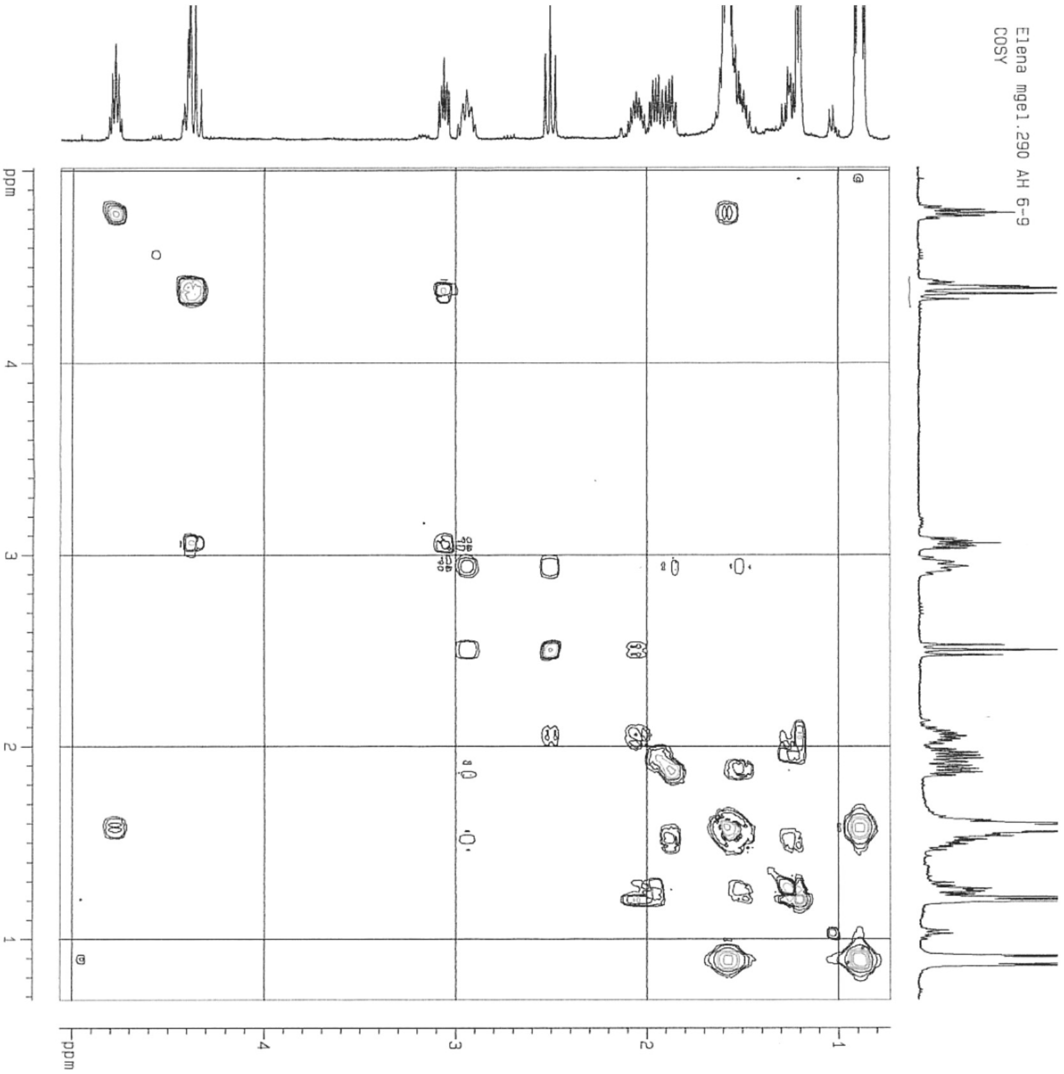
Elena mge1.290 AH 6-9
COSY
ppm

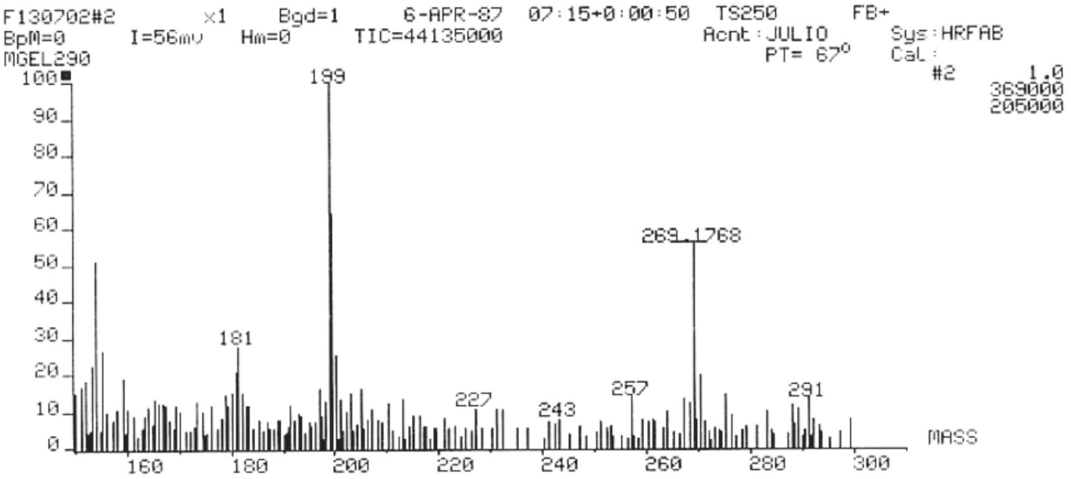
F130702#2 ×1 Bgd=1 6-APR-87 07:15+0:00:50 TS250 FB+
BpM=0 I=56mV Hm=0 TIC=44135000 Acnt:JULIO Sys:HRFAB
MGEL290 PT= 67° Cal:
#2 1.0
369000
205000
199
269.1768
181
227
243
257
291
MASS
160
180
200
220
240
260
280
300
